# A Bidentate Iodine(III)‐Based Halogen‐Bond Donor as a Powerful Organocatalyst**

**DOI:** 10.1002/anie.202013172

**Published:** 2021-01-15

**Authors:** Flemming Heinen, Dominik L. Reinhard, Elric Engelage, Stefan M. Huber

**Affiliations:** ^1^ Fakultät für Chemie und Biochemie Ruhr-Universität Bochum Universitätsstrasse 150 44801 Bochum Germany

**Keywords:** Diels–Alder cycloaddition, halogen bonding, hypervalent iodine, noncovalent interactions, organocatalysis

## Abstract

In contrast to iodine(I)‐based halogen bond donors, iodine(III)‐derived ones have only been used as Lewis acidic organocatalysts in a handful of examples, and in all cases they acted in a monodentate fashion. Herein, we report the first application of a bidentate bis(iodolium) salt as organocatalyst in a Michael and a nitro‐Michael addition reaction as well as in a Diels–Alder reaction that had not been activated by noncovalent organocatalysts before. In all cases, the performance of this bidentate XB donor distinctly surpassed the one of arguably the currently strongest iodine(I)‐based organocatalyst. Bidentate coordination to the substrate was corroborated by a structural analysis and by DFT calculations of the transition states. Overall, the catalytic activity of the bis(iodolium) system approaches that of strong Lewis acids like BF_3_.

## Graphical abstract

The first application of a bidentate iodine(III)‐based halogen bond donor as organocatalyst is presented. In three benchmark reactions, it markedly outperforms “classical” iodine(I)‐based noncovalent organocatalysts. The bidentate mode of activation as well as the crucial relevance of halogen bonding are confirmed by comparison experiments.

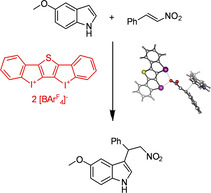

Halogen bonding (XB) describes the non‐covalent interaction between an electrophilic halogen substituent (called “XB donor”) and a Lewis base (called “XB acceptor”).^[1]^ It has found broad application^[2]^ in numerous fields like crystal engineering,^[3]^ molecular and anion recognition,[^1c^, ^4^] as well as peptide chemistry.^[5]^ In the last decade, halogen bonding has also been established in organocatalysis and has been applied in various organic reactions.^[6]^ So far, virtually all employed halogen bond donors were based on iodine(I) derivatives, with backbones ranging from polyfluorinated arenes to imidazolium or triazolium derivatives.^[7]^ As expected, cationic XB donors are more potent catalysts than neutral ones,^[8]^ and bidentate variants often severely outperform their monodentate analogs. Thus, the most potent currently available XB catalysts—next to elemental iodine^[9]^—typically rely on two cationic iodine(I)‐based XB‐donating moieties (e.g. iodobenzimidazolium groups;^[7d]^ Figure 1, left).

**Figure 1:**
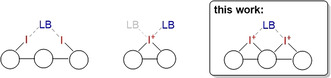
Bidentate iodine(I)‐based XB (left), monodentate iodine(III)‐based XB (middle), and bidentate iodine(III)‐based XB (right).

Iodine(III)‐derived compounds, on the other hand, are very versatile reagents in various organic transformations,^[10]^ especially for oxidations of functional groups^[11]^ and for transition‐metal‐catalyzed^[12]^ or direct arylations.^[13]^ Diaryliodonium salts typically feature a T‐shaped structure around the central iodine atom in which the anion is coordinated by “secondary bonding”.[^11a^, ^14^] This interaction can be described as XB,^[15]^ and the corresponding Lewis acidity of iodine(III) derivatives is of growing interest in recent years, for example, in theoretical studies.^[16]^ In pioneering experimental work, Legault et al. quantified the Lewis acidity of several iodonium salts using various titration techniques.^[17]^ After Han and Liu had reported the use of diaryliodonium salts as catalysts in a Mannich reaction,^[18]^ our group demonstrated that the catalytic activity of cyclic iodolium salts in a halide abstraction reaction and a Diels–Alder cycloaddition is indeed very likely due to XB.^[19]^ Further halide abstraction reactions were subsequently published by Aoshima,^[20]^ Nachtsheim,^[21]^ and our group (activating a metal−halogen bond^[21]^).

In several of these reactions,[^19^, ^22^] the iodolium catalysts showed comparable activity to “classical” bidentate iodine(I) XB donors. This strong performance is particularly noteworthy since the iodine(III) derivatives act as monodentate XB donors (Figure 1, middle), even though they in principle feature two electrophilic axes.[^15^, ^16^, ^23^, ^24^] All chemical intuition suggests, however, that bidentate iodolium variants should be markedly more Lewis acidic and should likely surpass the currently best catalysts in activity (Figure 1, right). Herein, we present the first application of such a bidentate iodine(III)‐based XB donor as organocatalyst.

As suitable core structure, thiophene‐linked bis(iodolium) triflate **1** (Scheme 1) was selected, which had previously been published by Yoshikai.^[25]^ It features a very rigid binding pocket, and is topologically related to a dithienothiophene‐derived chalcogen bonding organocatalyst introduced by Matile.^[26]^

**Scheme 1:**
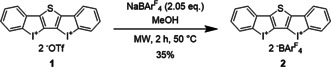
Synthesis of XB donor **2** via salt metathesis.

Single crystals of compound **1** were obtained by slow evaporation of acetonitrile (Figure 2).

**Figure 2:**
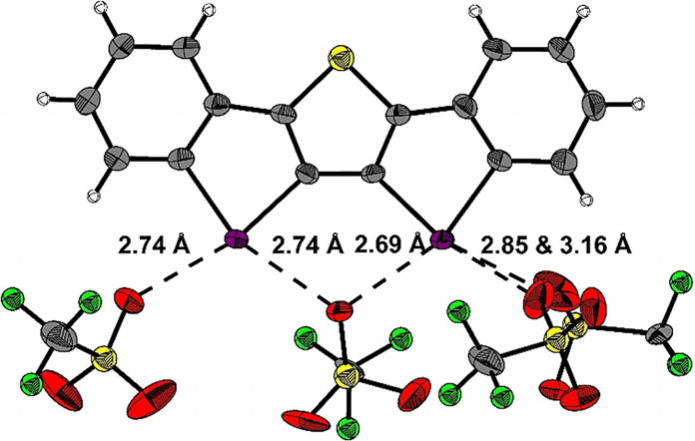
Excerpt of the X‐ray structural analysis of XB donor **1**.^[40]^ The triflate anions are disordered. Ellipsoids at 50 % probability. Gray C, white H, green F, purple I, red O, yellow S.

The structure clearly shows bidentate XB between the iodine centers and one oxygen atom of a counterion (which lies in the plane formed by the I‐C‐C‐I unit). Typical for XB, both I⋅⋅⋅O distances (2.69–2.73 Å) are markedly shorter than the sum of the van der Waals radii (3.50 Å)^[27]^ and the corresponding C‐I⋅⋅⋅O angles (159–160°) are almost linear. The remaining electrophilic axes on the iodines are also coordinated by triflates, with one strongly bound anion (Figure 2, left side) and two moderately bound ones (Figure 3, right side).^[28]^

**Figure 3:**
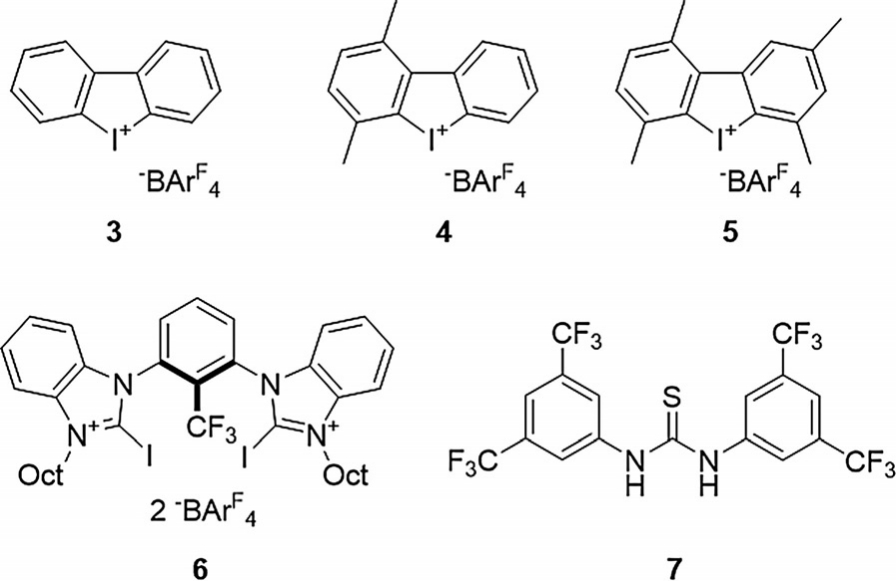
Lewis acidic organocatalysts employed as reference compounds in this study.

In the past, triflate counterions often hindered the activity of XB donors by outcompeting neutral Lewis bases.[^7c^, ^7f^, ^19^] To overcome this blockage and to drastically increase the solubility of **1**, non‐coordinating tetrakis[3,5‐bis(trifluoromethyl)phenyl]borate (BAr^F^_4_) counterions were introduced via our recently published method,^[22]^ which provided XB donor **2** as a dietherate complex with 35 % yield (Scheme 1).

With this promising XB donor in hand, our goal was to determine its activity in a series of increasingly challenging transformations and to compare its results to several reference compounds (Figure 3): monodentate variant **3** as well as its derivatives with one (**4**) or both (**5**) electrophilic axes blocked,^[19]^ our currently strongest XB donor **6**,^[7d]^ and a representative hydrogen bonding (HB) organocatalyst **7**.^[29]^

As a first benchmark reaction we focused on the Michael addition reaction between 1‐methylindole (**8**) and *trans*‐β‐crotonophenone (**9**) (Scheme 2), which is known to be less reactive towards hidden Brønsted acids.^[30]^ It can, however, be catalyzed through XB with molecular iodine^[30]^ and cationic iodine(I) donors.[^7e^, ^7g^, ^31^] In contrast to earlier studies, we started our initial experiments with a 10 mol % catalyst loading and already observed a strong activity of XB donor **2**, yielding 74 % of product **10** after 1 hour and full conversion after 9 hours (Figure 4). Reducing the catalyst loading to only 1 mol % still provides a satisfying conversion of 62 % after 12 hours. Comparison experiments with monodentate iodolium compounds **3**, **4**, and **5** did not result in any product formation even when 20 mol % of catalyst were used. This confirms that the enhanced activity of **2** is not merely the result of the presence of two iodine centers, but very likely the consequence of bidentate binding.

**Figure 4:**
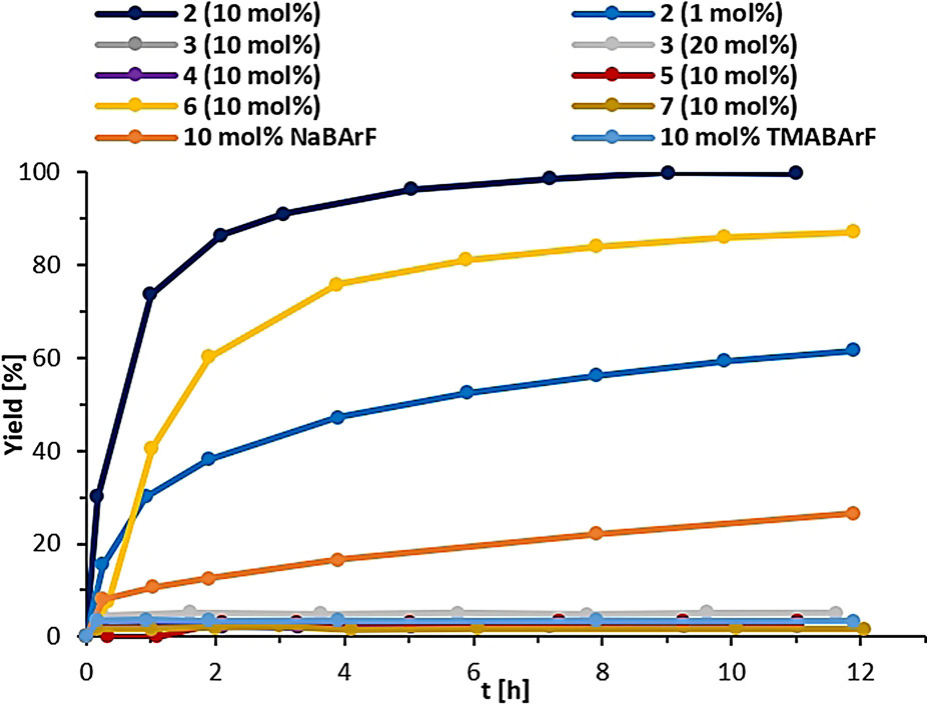
Yield‐vs.‐time profile of the Michael addition between **8** and **9** in the presence of different halogen bond donors.

**Scheme 2:**
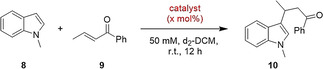
Michael addition of 1‐methylindole (**8**) to *trans*‐β‐crotonophenone (**10**) as benchmark reaction.

Sodium traces and BAr^F^_4_ as the active species can be ruled out by the fact that NaBAr^F^_4_ gave only 26 % conversion and that tetramethyl ammonium (TMA) BAr^F^_4_ was inactive. HB donor **7** was also inactive.^[32]^ In a direct comparison with our so far strongest XB donor **6**, only 41 % product was formed after 1 h and full conversion was not achieved within 12 h (Figure 4).

In parallel, DFT calculations (M06‐2X‐D3^[33]^ def2‐TZVP(D)^[34]^) were employed to obtain the transition state of the reaction involving catalyst **2**. Its structure clearly confirms bidentate XB between the carbonyl group and the two iodines, with C‐I⋅⋅⋅O distances of 2.56 Å and angles of 159° (Figure 5). The corresponding Gibbs free energy of activation is 13 kcal mol^−1^ (compared to 22 kcal mol^−1^ for the transition state involving catalyst **6**, see SI).

**Figure 5:**
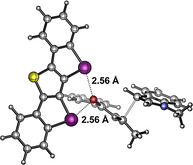
Transition state of the Michael addition reaction involving XB donor **2**, as obtained by DFT calculations. Graphic generated with CYLview.^[35]^ Gray C, purple I, blue N, red O, yellow S.

After this successful carbonyl activation, we were next interested in the nitro‐Michael reaction between 5‐methoxyindole (**11**) and nitrostyrene (**12**), which we had used before as benchmark (Scheme 3).[^7g^, ^36^]

**Scheme 3:**
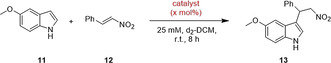
Nitro‐Michael addition reaction between 5‐methoxyindole (**11**) and nitrostyrene (**12**) in the presence of different XB donors.

Even under decidedly more challenging conditions than in our earlier study (lower overall concentration, 1:1 ratio of starting materials, lower catalyst loading of 5 mol %),^[7g]^ 83 % product formation was already observed after only 1 h and the reaction was completed after 3 h when XB donor **2** was used (Figure 6). Again, just 1 mol % of XB donor **2** still yielded 55 % of product **13** after 8 hours. In ^1^H NMR titration experiments, binding constants for the adduct of catalyst **2** and substrate **12** of *K*=29 m ^−1^ as well as for the adduct of the same catalyst and product **13** of *K*=18 m ^−1^ were revealed.^[37]^

**Figure 6:**
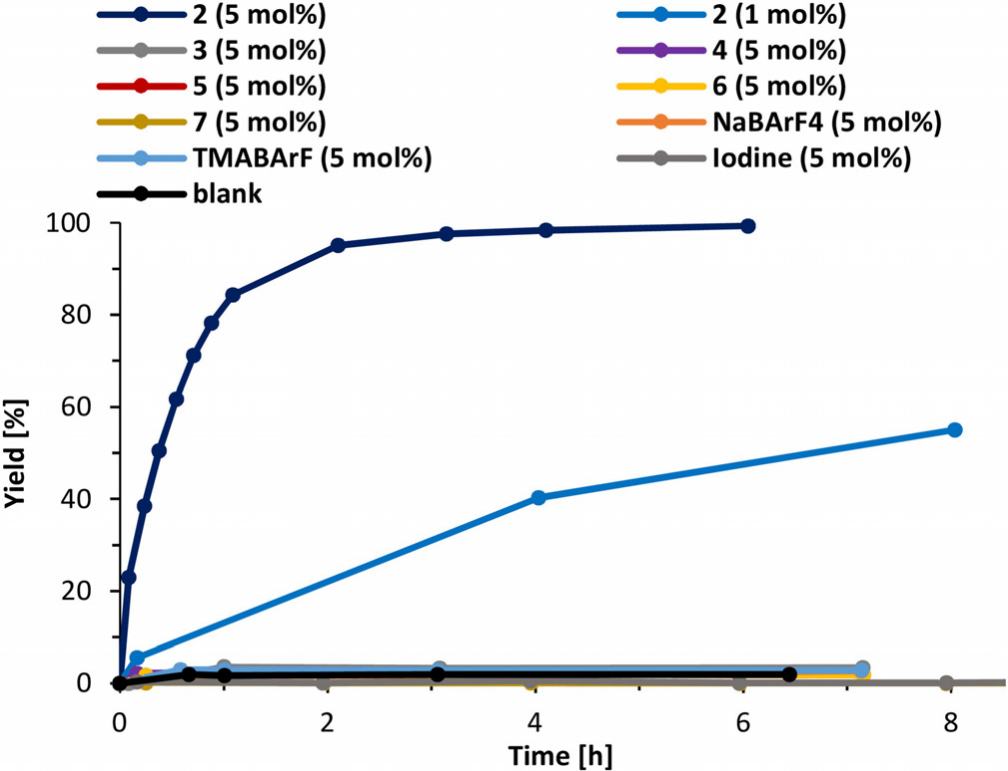
Yield‐vs.‐time profile for the nitro‐Michael addition between **11** and **12** with various catalysts.

Monodentate iodolium salts **3**, **4**, and **5** are inactive, as is the bidentate XB donor **6** (which led to 40 % product formation after almost 50 h with a catalyst loading of 20 mol % in our earlier study).^[7g]^ This stark difference in activity vividly illustrates how much more powerful the bidentate iodine(III)‐based XB donor is compared to a similarly preorganized iodine(I) variant. Hidden sodium catalysis and any counterion effect can once again be excluded, as NaBAr^F^_4_ and TMA BAr^F^_4_ are inactive as well. The same is true for HB organocatalyst **7** and for molecular iodine, a strong XB donor.^[9]^

Just like for the Michael reaction discussed above, DFT calculations on the likely transition state also yielded a structural model featuring bidentate coordination of the catalyst to one oxygen of the nitro group (Figure 7). The C‐I⋅⋅⋅O distances are 2.62 and 2.69 Å, with XB angles of 158–160°. The associated Gibbs free energy of activation is 17 kcal mol^−1^ (while, interestingly, these orientating calculations yield a barrier of only 13 kcal mol^−1^ for the transition state involving catalyst **6**).

**Figure 7:**
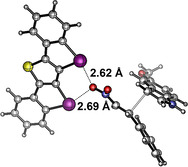
Transition state of the nitro‐Michael addition reaction involving XB donor **2**, as obtained by DFT calculations. Graphic generated with CYLview.^[32]^ Gray C, purple I, blue N, red O, yellow S.

Finally, we focused on a Diels–Alder reaction to further illustrate the catalytic activity of catalyst **2**, once again opting for a challenging case. Thus, in contrast to our earlier benchmark reaction between methyl vinyl ketone (**15**) and 10 equivalents of cyclopentadiene,[^7e^, ^7g^, ^19^] we now employed just one equivalent of less reactive cyclohexadiene (**14**) as diene (Scheme 4).

**Scheme 4:**
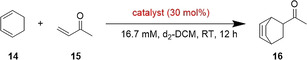
Diels–Alder reaction between 1,3‐cyclohexadiene (**14**) and methyl vinyl ketone (**15**) with various XB donors.

With 30 mol % of catalyst **2**, 73 % of product **16** was formed within 12 h (Figure 8). The use of similar amounts of monodentate iodolium salts **3**, **4**, and **5** as well as of bidentate iodine(I) donor **6** or of molecular iodine led to no reaction (Figure 8).^[38]^

**Figure 8:**
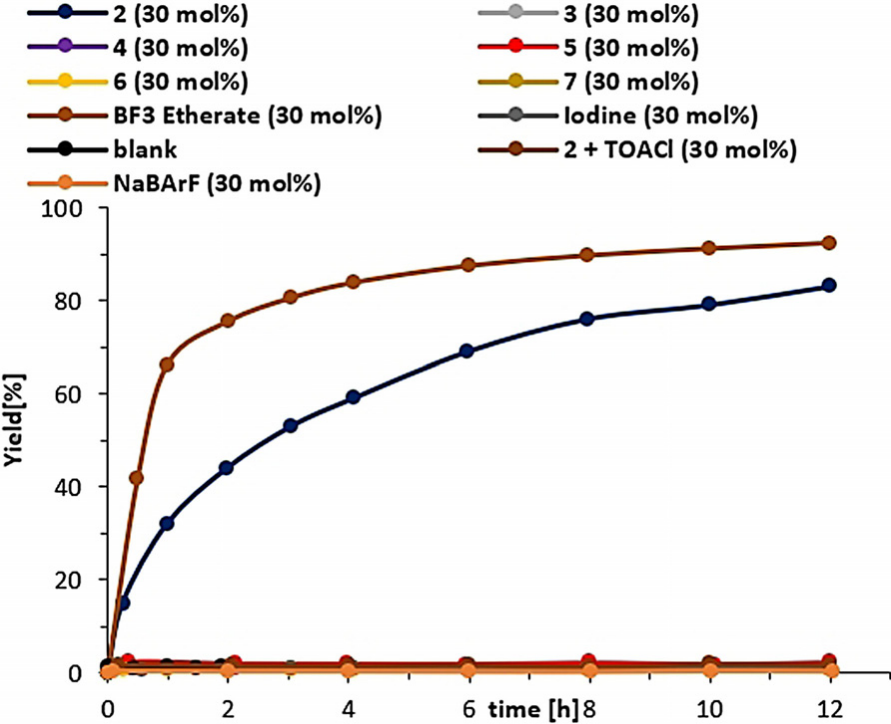
Yield‐vs.‐time profile for the Diels–Alder reaction between **14** and **15**.

Acid traces were also excluded as potentially catalytically active species: first, slow decomposition of the catalysts was ruled out by a repeated‐addition experiment, in which portions of starting materials **14** and **15** were added to the reaction mixture again after 18 h. The resulting similar reaction profile^[39]^ indicates that no catalytically active species was generated over time. Second, we observed that addition of HOTf to 1,3‐cyclohexadiene led to quick decomposition. Furthermore, the activity of catalyst **2** can be completely suppressed by pre‐mixing it with tetrabutyl ammonium chloride (TBACl), presumably by the chloride blocking the binding site of the catalyst.^[19]^ In addition, hidden Na^+^ catalysis was again ruled out due to the inactivity of NaBAr^F^_4_.

Subsequently, other Lewis acids were also employed for comparison. Catalysis of this reaction has not been reported with HB donors before, and indeed Schreiner's thiourea **7** as (neutral) organocatalyst induced no reaction. With the classical Lewis acid BF_3_−etherate, a faster reaction compared to **2** was found (92 % yield of product), while others such as AlCl_3_ and Zn(OTf)_2_ failed under these conditions due to their low solubility in DCM. Even though the performance of XB donor **2** is a bit lower than the one of BF_3_−etherate, this roughly comparable activity still represents, to the best of our knowledge, the first case in which a synthetic XB donor reaches the strength of such Lewis acids.

In conclusion, the first application of a bidentate iodine(III)‐based XB donor in organocatalysis was presented. In three benchmark reactions featuring either the activation of a carbonyl or a nitro group, this catalyst decidedly outperformed monodentate variants as well as our formerly strongest iodine(I)‐based organocatalyst **6**, with it being twice the only catalyzing system. This highly preordered bis(iodolium) derivative is thus approaching the potency of Lewis acids like BF_3_. A bidentate mode of activation was clearly indicated by comparison experiments, a solid‐state structure, and DFT calculations. We anticipate that this class of XB donors will find frequent use in organocatalysis, and further studies in this regard are underway.

## Conflict of interest

The authors declare no conflict of interest.

## Supporting information

As a service to our authors and readers, this journal provides supporting information supplied by the authors. Such materials are peer reviewed and may be re‐organized for online delivery, but are not copy‐edited or typeset. Technical support issues arising from supporting information (other than missing files) should be addressed to the authors.

Supplementary
