# Supplementary material for: A Bidentate Iodine(III)‐Based Halogen‐Bond Donor as a Powerful Organocatalyst
Source: Angew Chem Int Ed Engl. 2021 Jan 15;60(10):5069–73. doi: 10.1002/anie.202013172 (PMC7986438; doi:10.1002/anie.202013172)
Supplement: Supplementary file 1 — Supplementary [file ANIE-60-5069-s001.pdf]

Angewandte Chemie
Eine Zeitschrift der Gesellschaft Deutscher Chemiker

Supporting Information

A Bidentate Iodine(III)-Based Halogen-Bond Donor as a Powerful Organocatalyst**

*Flemming Heinen, Dominik L. Reinhard, Elric Engelage, and Stefan M. Huber**

anie_202013172_sm_miscellaneous_information.pdf

Contents

1. Experimental Section **3**
1.1 General Remarks 3
1.2 Analysis Methods 3
1.3 Synthesis 5
1.4 ^{1}H-NMR reaction setup 7
1.5 ^{1}H-NMR titration experiments 14

2. NMR-Spectra **16**
2.1 ^{1}H-, ^{13}C- and ^{19}F-NMR Spectra of **2** 16
2.2 Periodic ^{1}H-NMR spectra of Michael-Addition 17
2.3 Periodic ^{1}H-NMR spectra of the nitro Michael Addition 22
2.4 Periodic ^{1}H-NMR spectra of the Diels-Alder reaction 27

3. DFT Calculations **33**
3.1 Method 33
3.2 Data 33

4. XRD Data **71**
4.1 Crystal Growth 72

5. Literature **76**

1. Experimental Section

1.1 General Remarks

Unless otherwise noted, all solvents were obtained in technical grade quality and purified by distillation prior to use. Thin-layer chromatography was performed by using *Merck TLC aluminium sheets* (silical gel 60, F254). Column chromatography was performed with silica gel (grain size 0.04-0.063 cm, *Macherey-Nagel Si60*) at atmospheric pressure (1-1.5 atm, using a hand pump in some cases). The corresponding solvents that were used as eluents as well as the R_f values are listed at the corresponding experiment. Detection of the substances was achieved by fluorescence under UV light (wavelength λ = 254 nm).

1.1.1 Solvents

Dry DCM, ether and THF were received from a *MBRAUN MB SPS-800*. Solvents were distilled and dried over 4 Å molecular sieve. Further dry solvents were dried over flame dried 4 Å molecular sieves. The residual water content was determined with a Karl Fischer *Titroline 7500KF trace* from *SI Analytics* with *Honeywell (Fluka) Hydranal Coulomat-AD* solution.

1.1.2 Chemicals

Chemicals were obtained from *ABCR, Alfa Aesar, Carbolution Chemicals, Merck, ChemPur, Fluorochem, Sigma Aldrich* and were used without further purification (unless mentioned otherwise).

1.2 Analysis Methods

1.2.1 NMR Spectroscopy

^{1}H-NMR spectra and ^{13}C-NMR spectra were recorded on a *Bruker DPX-250 NMR* or *Aviii 300* spectrometer at 300 K. ^{19}F-NMR spectra were recorded on a *Bruker DPX-250 NMR* at 300 K. Peaks were referenced to residual ^{1}H signals and ^{13}C signals from deuterated solvents and are reported in parts per million (ppm). NMR-spectra were analyzed with *MestReNova 9.0*. [S1] For ^{1}H-NMR spectroscopically data, ^{13}C-NMR spectroscopically data and ^{19}F-NMR spectroscopically data, multiplicity (s = singlet, d = doublet, dd = doublet of doublet, ddd = doublet of doublet of doublet, dddd = doublet of doublet of doublet of doublet, t = triplet, td = triplet of doublet, m = multiplet), the relative integral and the coupling constant (*J* in *Hz*) are indicated if possible.

1.2.2 ATR-IR Measurements

IR spectra were recorded on a *Shimadzu IR Affinity – 1S* spectrometer with a *Specac-Quest* ATR and are reported in ν = cm^{-1} and are indicated with w (weak), m (middle), s (strong) or vs (very strong).

1.2.3 **EI and ESI Measurements**

Mass spectra were recorded on either a *Bruker Daltonics Esquire 6000* instrument (ESI) a *Jeol AccuTOF (EI-MS)*, or an *Agilent 7820GC/5977B-EIMSD*, equipped with a 30 m *HP5MS* column using Helium as carrier gas.

1.2.4 **Elemental Analysis**

CHNS Elemental Analysis was performed on a *vario Micro cube* from *Elementar Analysentechnik*.

1.2.5 **XRD Measurements**

XRD measurements were performed on a single crystal-X-ray-diffractometer Rigaku XtaLAB-Mini, equipped with a 600 W Mo micro-fine focus glass sealed tube, graphite monochromator (Mo Kα) and CCD detector. Crystals were mounted in Hampton CrypLoops using Parabar/Paratone or GE/Bayer silicone grease and cooled to 170K in a stream of cold air using an Oxford Desktop Cooler (DTC). Data was recorded and reduced using the CrysalisPro Software. Structures were solved using WinGX in combination with ShelXT and refined with shelXle and ShelXL. Tables for the publication were generated using a modified version of Ciftab. Pictures of the structures were generated with Diamond 4 [S2]

1.2.6 **Balance for Stock Solutions**

Starting materials for stock solutions were weighted on a *Mettler Toledo XSR 105 Dual Range* balance.

1.3 Synthesis

Compounds **3**,[S3] **4**,[S3] **5**[S3], **6**[S4] and **7**[S5] were synthesized according to published literature procedures.

1.3.1 Synthesis of 2,2´-(thiophene-2,5-diyl)dianiline

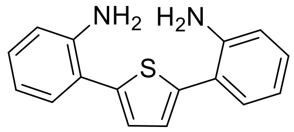

$C_{16}H_{14}N_2S$

266.09 g/mol

The title compound was synthesized via a Suzuki-Miyaura cross coupling reaction. 1.13 ml 2,5-Dibromothiophene (10 mmol), 5 g of 2-(4,4,5,5-Tetramethyl-1,3,2-dioxaborolan-2-yl)aniline (22.8 mmol, 2.28 eq.), 4.1 g K_2CO_3 (30 mmol, 3 eq.) and 0.578 g $Pd(PPh_3)_4$ (0,5 mmol, 0.05 eq.) were dissolved in 60 ml degassed 1,4-dioxane and stirred at 100 °C until full conversion was indicated by GC-MS (~72 h). The crude reaction mixture was allowed to cool to room temperature and was plugged through a celite plug with EtOAc as solvent. The solvent was removed and the crude product mixture purified via column chromatography (Pentane/EtOAc 4:1, R_f = 0.15) giving 2.06 g (7.73 mmol, 77%) of the title compound. The analytical data was consistent with literature protocols.[S6]

1.3.2 Synthesis of 2,2-bis(2-iodophenyl)thiophene

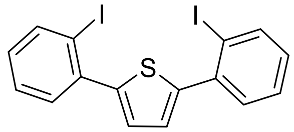

$C_{16}H_{10}I_2S$

487,86 g/mol

The title compound was synthesized according to literature protocols.[S6]

1.3.3 Synthesis of triflate salt of bidentate catalyst (1)

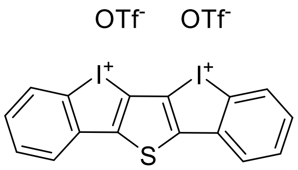

$C_{18}H_8F_6I_2O_6S_3$

784.24 g/mol

The title compound was synthesized according to literature protocols.[S6]

1.3.4 Synthesis of bis[tetrakis(3,5-bis(trifluormethl)phenyl)borate] salt of bidentate catalyst(2)

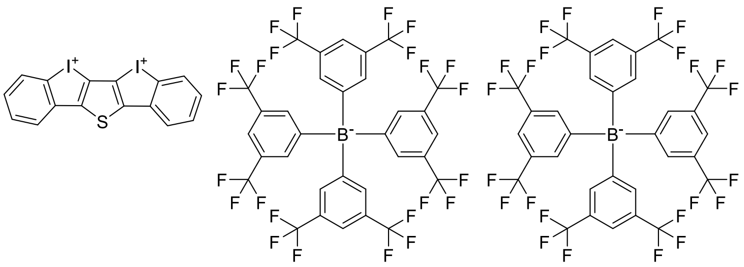

$C_{80}H_{32}B_2F_{48}I_2S$
2211.97 g/mol

The title compound was synthesized via a modified protocol published by our group recently.[S7] The corresponding triflate salt (500 mg, 0.638 mmol) and 1.16 g (1.31 mmol, 2.05 eq.) sodium tetrakis[3,5-bis(trifluormethyl)phenyl]borate ($NaBAr^F_4$) were placed in an open 20 ml microwave vessel, equipped with a stirring bar and subsequently 15 ml MeOH were added. The reaction vessel was placed in a *CEM Discover SP* microwave using the dynamic mode and was heated at 50 °C for 2 hours. The solvent was removed after cooling to room temperature. 15 ml DCM was added and the mixture stirred for 30 minutes. The formed solid was filtered off and pentane added upon which the product forms as an oil on the bottom of the flask. The solvent was decanted off, the residue redissolved in diethyl ether, and pentane was added. After decanting the solvent off, the residual oil was dried under HV yielding 530 mg (0.224 mmol, 35%) of the product as a diether complex.

^{1}H NMR (300 MHz, DMSO-*d*$_6$) δ: 8.33 (dd, *J* = 8.2, 2.0 Hz, 4H), 7.89 (t, *J* = 7.4 Hz, 2H), 7.77 – 7.65 (m, 10H), 7.65 – 7.57 (m, 16H), 3.35 (q, 8H), 1.07 (t, *J* = 7.0 Hz, 12H).
^{13}C NMR (75 MHz, DMSO-*d*$_6$) δ: 162.07 – 159.60 (m), 137.69, 135.87, 134.03, 131.68, 131.25, 130.82, 129.41, 128.66, 128.29, 125.80, 122.18, 118.58, 117.66, 64.89, 15.13.
^{19}F NMR (235 MHz, DMSO-*d*$_6$) δ: -61.70.

ATR-IR [$\tilde{\nu}$ = cm^{-1}]: 1610.56 (w), 1446.61 (w), 1352.10 (m), 1271.09 (s), 1111.00 (s), 975.98 (w), 885.33 (m) 837.11 (m), 756.10 (w), 744.52 (w), 711.73 (m), 680.87 (m), 669.30 (m), 580.57 (w), 449.41 (w).

ESI-MS:
m/z (+) = calc. 485.84 [M^+], found 485.64 [M^+]
m/z (-) = calc. 863.07 [BAr^F_4]$^-$, found 862.68 [BAr^F_4]$^-$
CHNS (%) = calc (+H_2O): C: 44.43, H: 2.29, S: 1.35; found: C: 42.27, H: 1.97, S: 1.37

1.4 ^{1}H-NMR reaction setup

Note: In contrast to the reactions featured in this paper, halide abstraction reactions would like require the use of stoichiometric amounts of the XB donor, as the latter is usually inhibited by the liberated halide.[S3]

1.4.1 Michael-type addition reaction

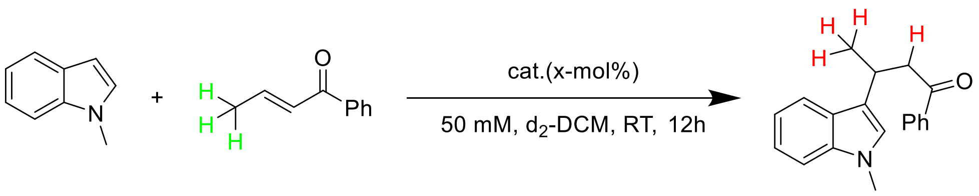

8 9 10

Scheme S1: Halogen bond catalyzed Michael addition reaction between 1-methylindole **8** and β-*trans*-crotonophenone **9**.

^{1}H-NMR catalysis experiments were performed analogously to published procedures.[S8] To a new NMR tube, freshly prepared stock solutions of the respective catalyst (200 µl, 0.1 eq. 15 mM), 1-methylindole **8** (200 µl, 1 eq., 150 mM) and β-*trans*-crotonophenone **9** (200 µl, 1 eq., 150 mM) in deuterated methylene chloride were added, sealed and the NMR tube shaken. Afterwards, periodically ^{1}H-NMR experiments (see Figure 1) were performed with a total duration of 12 hours at room temperature.

Experiments with 1 mol-%% catalyst: 20 µl (15 mM) catalyst stock solution + additional 180µl CD_2Cl_2.

Experiments with 20 mol-%: 200 µl (30 mM) of the respective catalyst stock solution.

As an internal standard 0.125 eq. of tetraethyl silane (TES) was added to the β-*trans*-crotonophenone **9** stock solution to check if decomposition occurred as well as to act as integration standard for the determination of the yield of compound **10**. The quartet of the TES signal was set to 1 and the integration of the characteristic signal at 3.28 - 3.16 directly gave the yield of compound **10**. Alternatively, the consumption of educt **9** (signal at 1.99, Figure S1 green) can be integrated vs. the formation of **10** (signal at 1.42, figure 2 red).

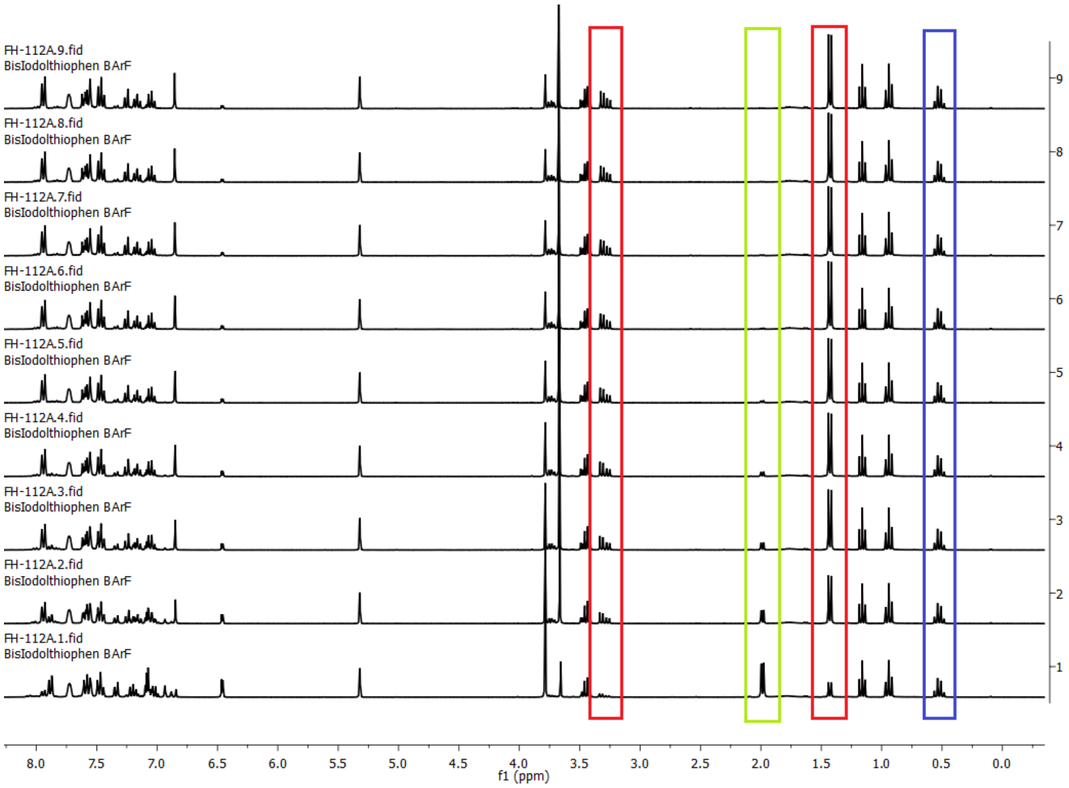

Figure S1: ^{1}H-NMR spectrum of the reaction between 1-methylindol **8** and b-*trans*-crotonophenone **9** in a 12 hour period. Marked in green: consumption of **9** (CH_3). In red: formation of product **10**; left: α-CH_2, right: CH_3. In blue: TES signal whose integral is set to 1.

1.4.2 Nitro Michael-type addition reaction

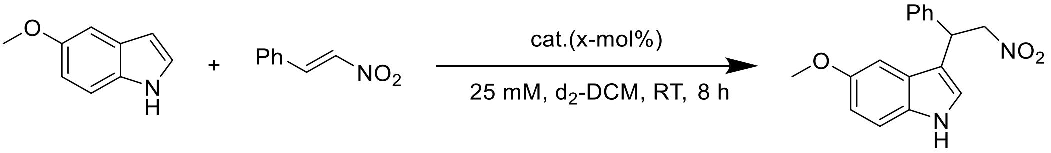

Scheme S2: Halogen bond catalyzed nitro Michael-type addition reaction of 5-methoxyindole **11** and nitrostyrene **12**

^{1}H-NMR catalysis experiments have been carried out modified to literature protocols.[S9] To a new NMR tube, freshly prepared stock solutions of the respective catalyst (50 µl, 0.05 eq., 15 mM), 5-methoxyindole **11** (200 µl, 1 eq., 75 mM) and nitrostyrene **12** (200 µl, 1 eq., 75 mM) in deuterated methylene chloride and d_2-DCM added to a total volume of 600 µl added, sealed and the NMR tube shaken. Afterwards, periodically ^{1}H-NMR experiments (Figure S2) were performed with a total duration of 7-8 hours at room temperature.

As internal standard 0.25 eq. TES were added to the nitrostyrene **12** stock solution to check if any decomposition occurred as well as to act as integration standard for the determination of the yield of compound **13.** The triplet of the TES (0.85 ppm) was set as 1 and the integration of the characteristic methoxy singlet at 3.65 ppm gave the yield of **13**. The yield was double checked by integration of the methoxy singlet at 3.73 ppm of **11.**

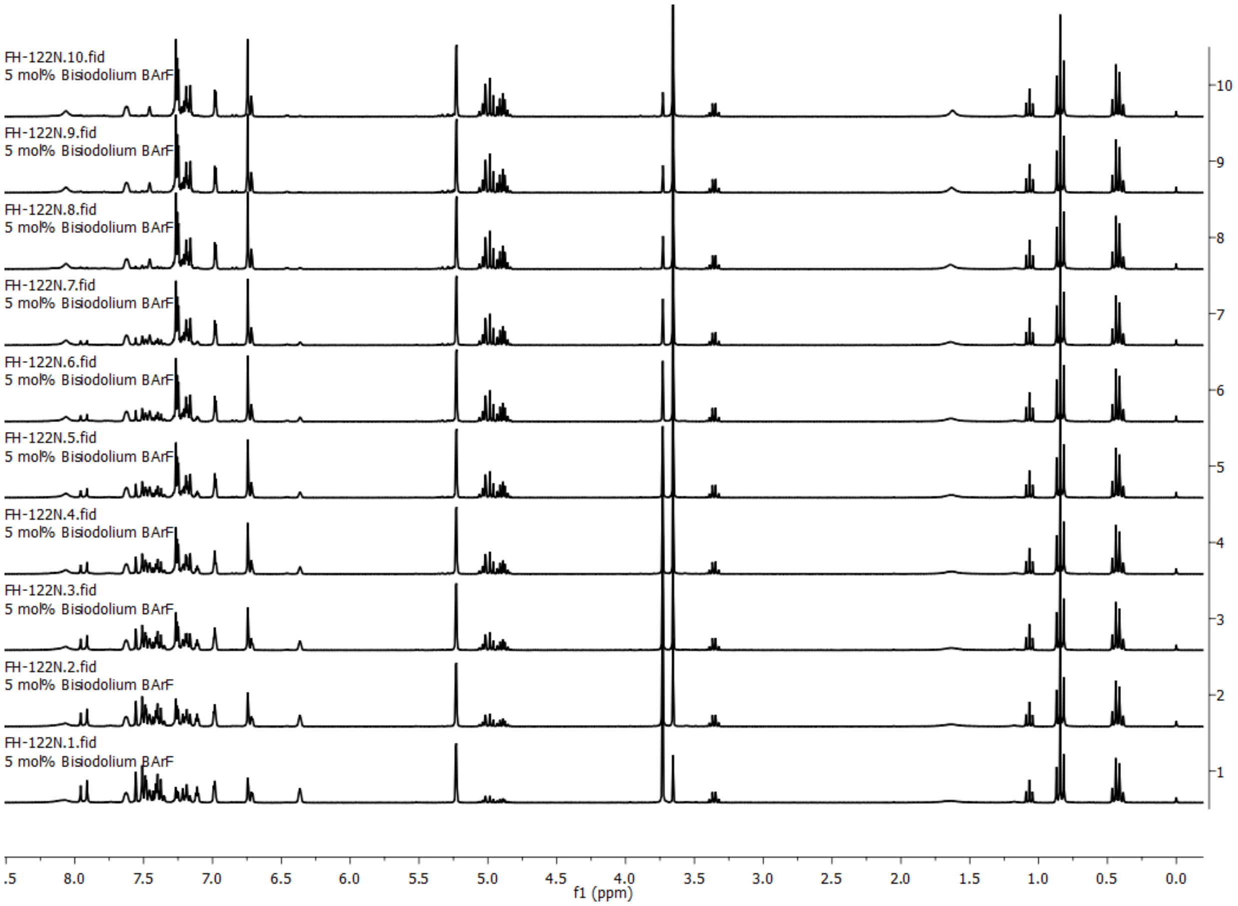

Figure S2: Periodic ^{1}H-NMR experiment spectrum of the nitro-Michael-type addition reaction between 5-methoxyindole **11** and nitrostyrene **12** with 5 mol-% **2** in a 6 hour time period.

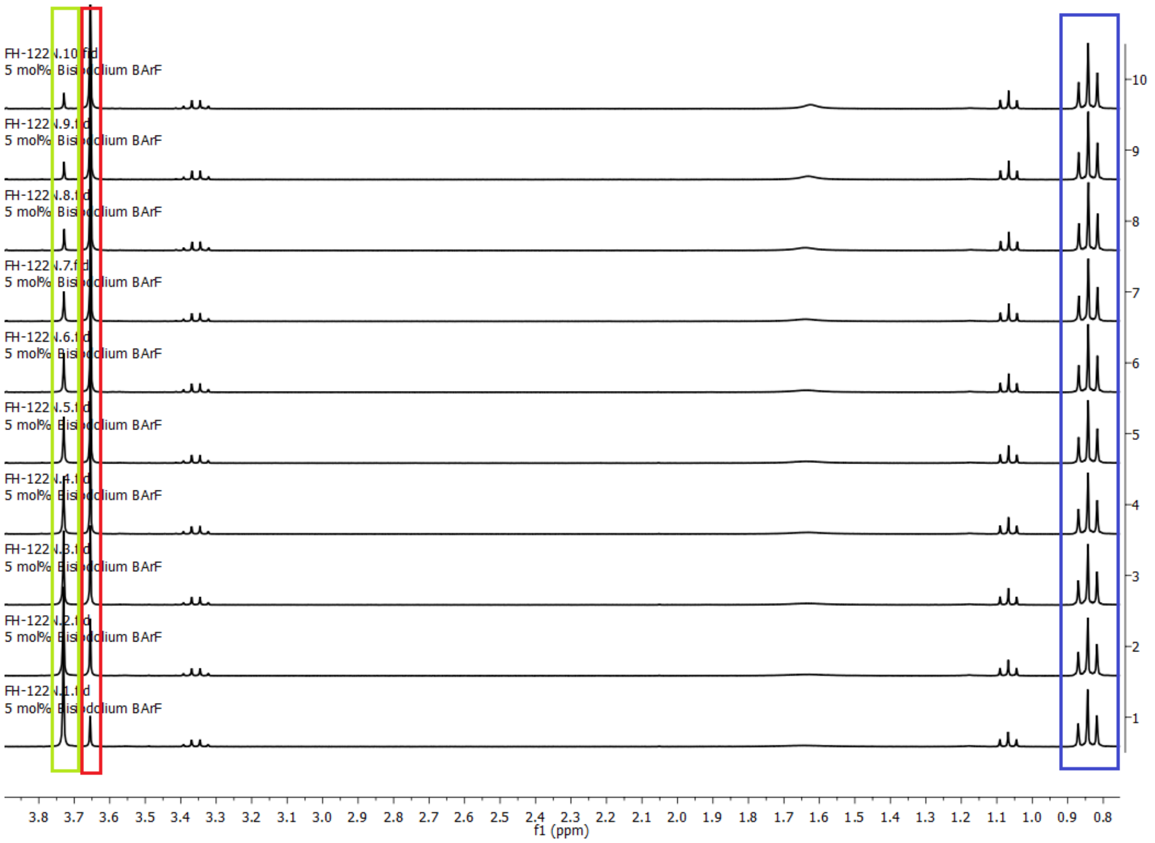

Figure S3: Zoom into the for the yield determination relevant ^{1}H-NMR of the Nitro Michael-Addition reaction between 5-methoxyindole **11** (methoxy-CH_3 in green) and nitrostyrene **12**. The methoxy CH_3 signals of product **13** is indicated in red and the corresponding TES in blue.

1.4.3 **Diels-Alder cycloaddition reaction**

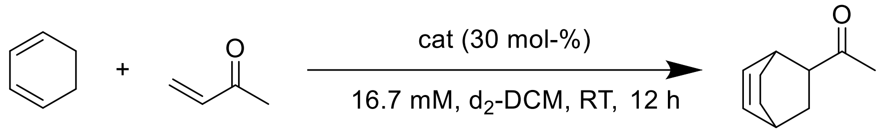

14 **15** **16**

To a new NMR tube, freshly prepared stock solutions of the corresponding catalysts (200 µl, 0.2 eq., 15 mM), 1,3-cyclohexadiene **14** (200 µl, 1 eq., 50 mM) and methyl vinyl ketone **15** (200 µl, 1 eq., 50 mM) in deuterated dichloromethane were added, sealed and shaken. Afterwards periodic ^{1}H-NMR experiments were performed with a total duration of 12 hours at room temperature.

Experiments with premixed catalyst and TOACl were performed in doubled concentration (30 mM each) and 100 µl stock solution each.

As internal standard 0.25 eq. TES were added to the MVK **15** stock solution to check if any decomposition occurred as well as to act as integration standard for the determination of the yield of compound **16**. The triplet of the TES (0.85 ppm) was set as 3 and corrected if necessary. Integration of the characteristic signals at 2.52 ppm, 2.69 ppm and 2.80ppm from **16** (Figure S4 and Figure S5 in detail, all are marked signal are equal) directly gave the yield.

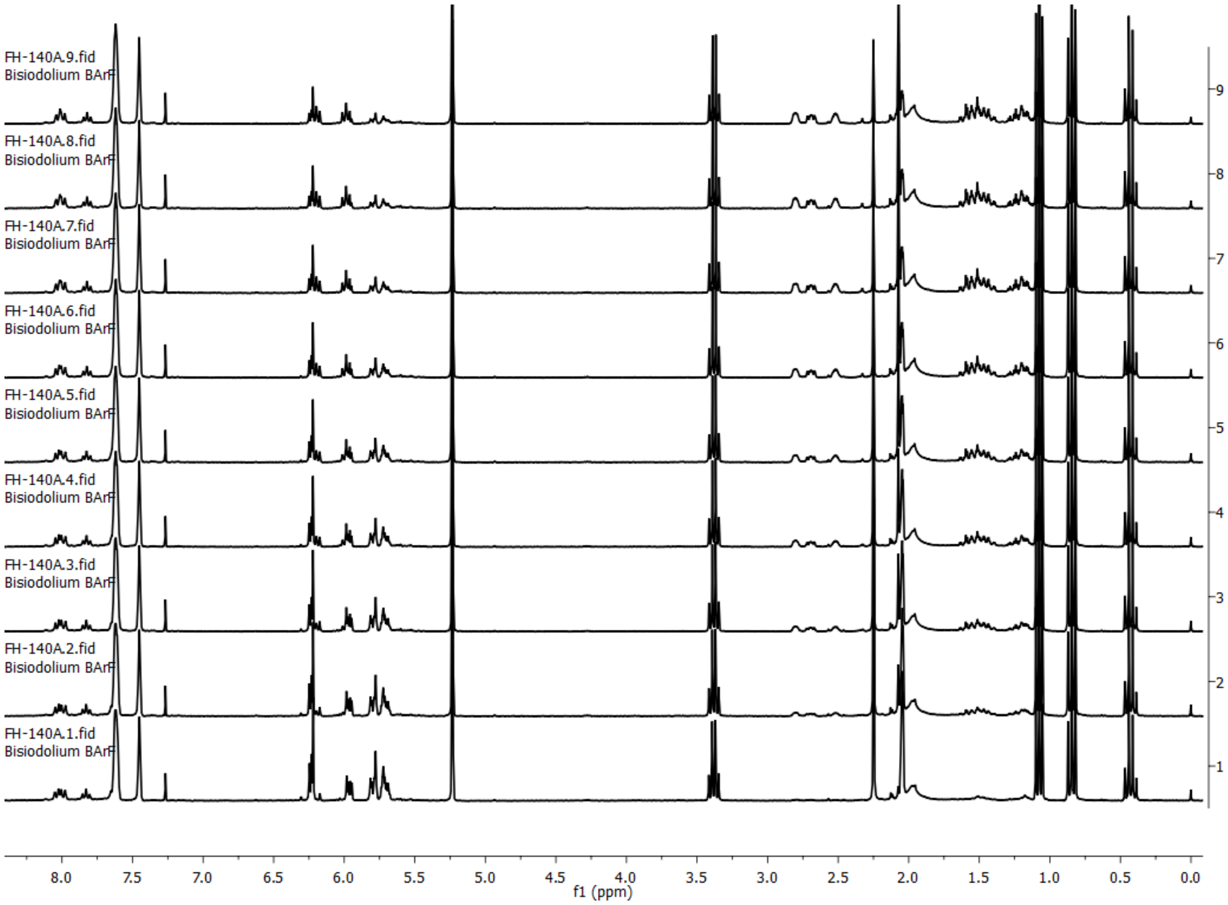

Figure S4: Periodic ^{1}H-NMR experiments of the Diels-Alder cycloaddition reaction between 1,3-cyclohexadiene **14** and methyl vinyl ketone **15** with 30 mol-% **2** in a total duration of 12 hours at room temperature.

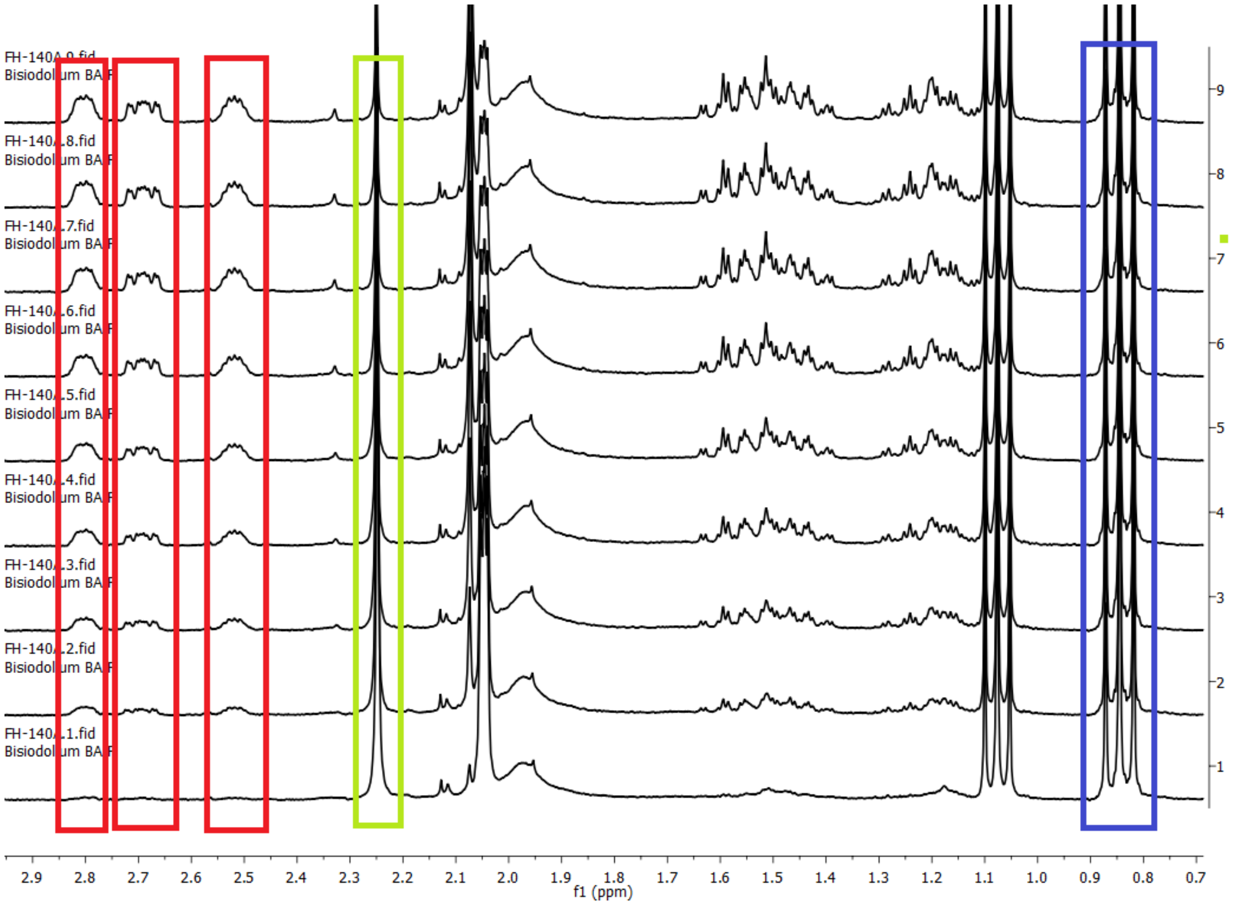

Figure 5: Zoom into the relevant area for the determination of yields during the ^{1}H-NMR periodic experiments in the Diels-Alder cycloaddition between 1,3-cyclohexadiene **14** and methyl vinyl ketone **15**. In red: equally integratable protons of product **16**. In green: Consumption of -CH_3 singlet of **15**. In Blue: triplet TES as internal standard.

1.4.4 Stability Test of Catalyst 2

The reaction was set up as described above. After 18 hour and 92% yield, a second dose of both starting material solutions **14** and **15** were added and the reaction was monitored for further 30 hours.

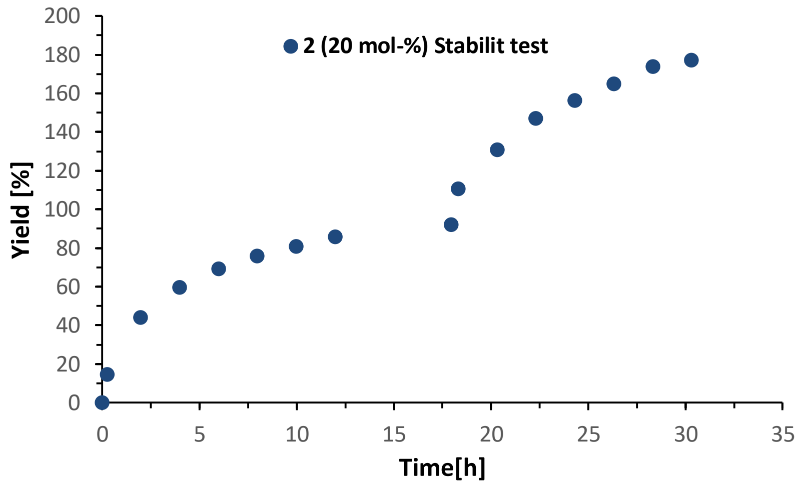

Figure S6: Reaction profile of the stability test of catalyst **2** in the Diels-Alder reaction between 1,3-cyclohexadiene **14** and **15.** After 18 hours a second dose of both starting material stock solution were added.

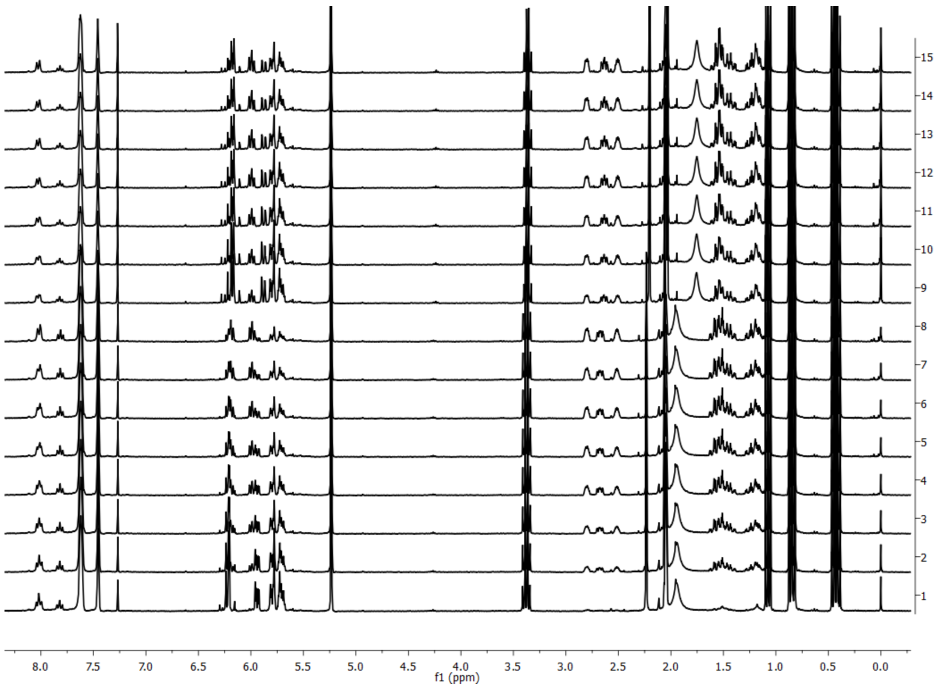

Figure S7: Periodic ^{1}H-NMR spectra of the Diels-Alder reaction between **14** and **15** with 30 mol-% of catalyst **2** in the stability test.

1.5 ^{1}H-NMR titration experiments

For pipetting, *Hamilton*®-syringes were used. All experiments were conducted at ambient temperature and in *Norell*® 502 NMR-Tubes. 30 µmol of the corresponding iodolium compound (host) were dissolved in 1 ml of CD_2Cl_2. Stock solutions of the guest were prepared as 0.15 M solutions in CD_2Cl_2. The NMR-tube was charged with 100 µl of the corresponding host solution and 500 µl CD_2Cl_2 was added. For each measurement point a certain amount of guest solution was added. The NMR-spectra were measured with an *Aviii 300* spectrometer at 298 K. Each ^{1}H-NMR was measured with 32 scans and referenced to the deuterated solvent. The host/guest ratio was checked in each spectrum by integration of the signals and was corrected if necessary.

For the determination of the binding constants the shifts of the protons of the host were observed relative to the deuterated solvent. The collected data (measured shifts in Δppm vs guest equivalents) was fitted via the website *supramolecular.org*, fitting the shift of the nuclei *ortho* to the iodine centers.

For the calculations of the binding constants (K in M^{-1}) a 1:1 stoichiometry was assumed.

Each experiment was repeated at least once.

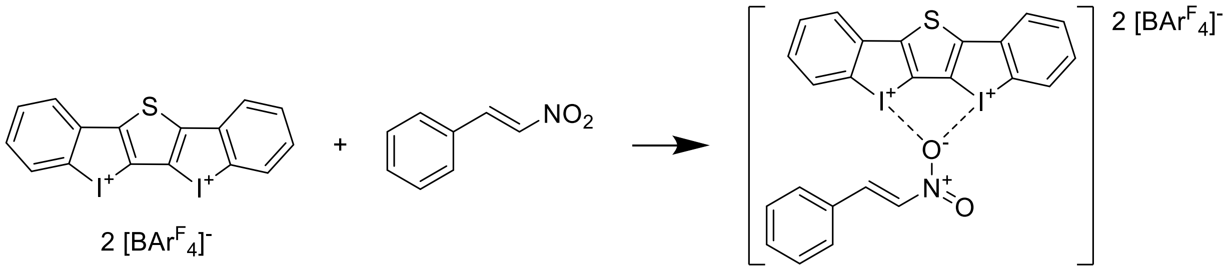

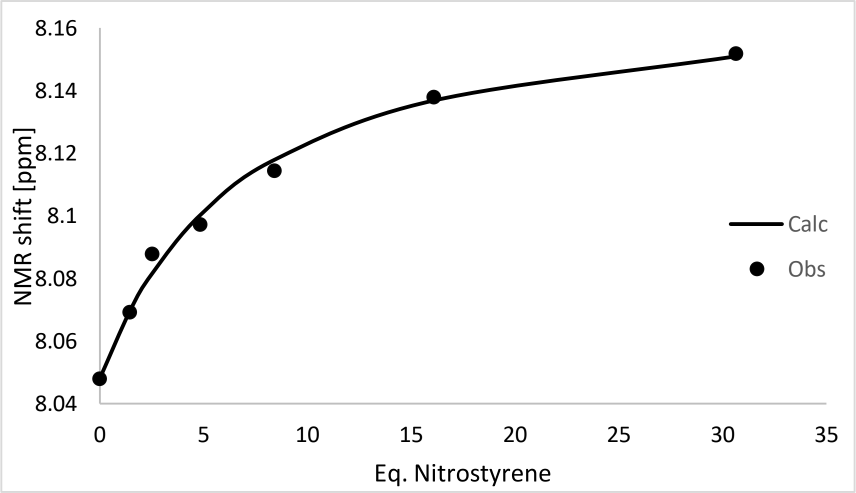

Figure S8: ^{1}H-NMR Titration between **2** and nitrostyrene **12**. K = 29 M^{-1}

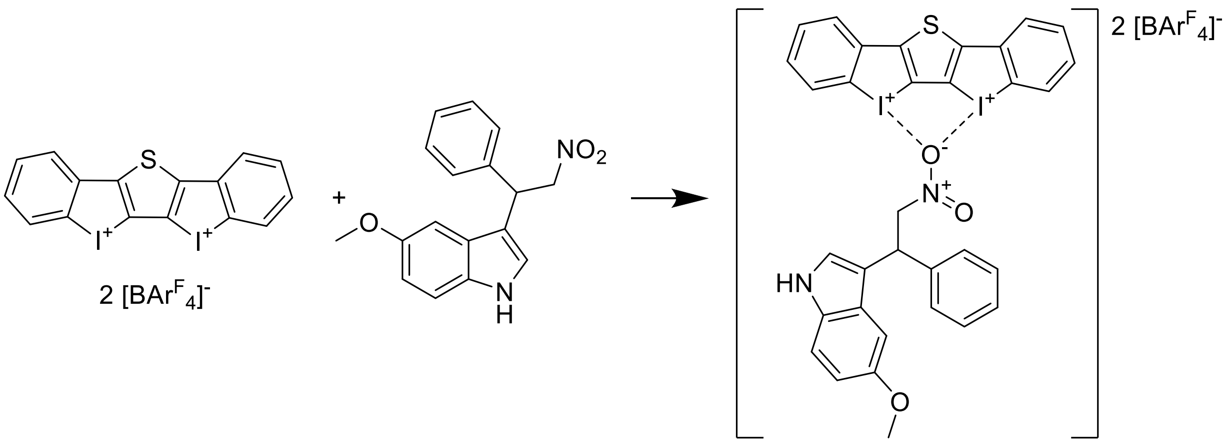

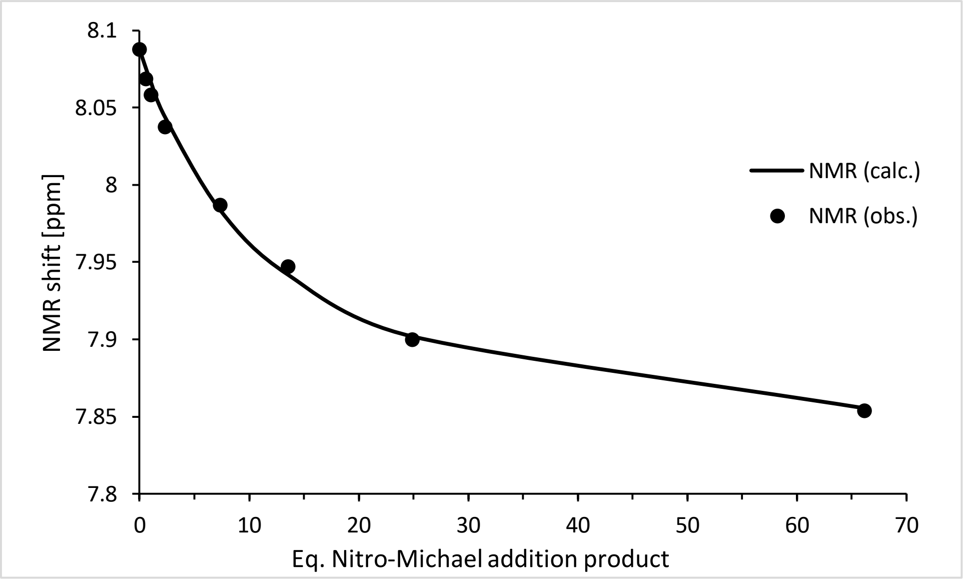

Figure S9: ^{1}H-NMR Titration between **2** and Nitro-Michael addition product **13**. K = 18 M^{-1}

2. NMR-Spectra

2.1 ^{1}H-, ^{13}C- and ^{19}F-NMR Spectra of 2

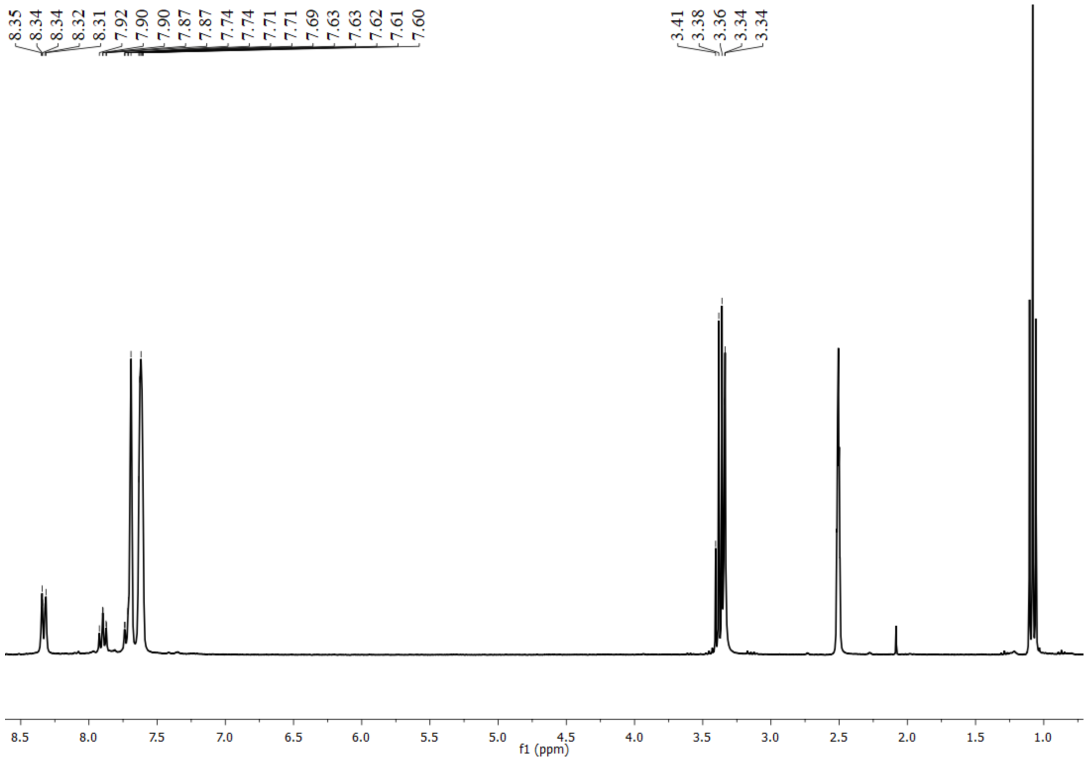

Figure S10: 300 MHz ^{1}H-NMR spectrum of **2** in d_6-DMSO.

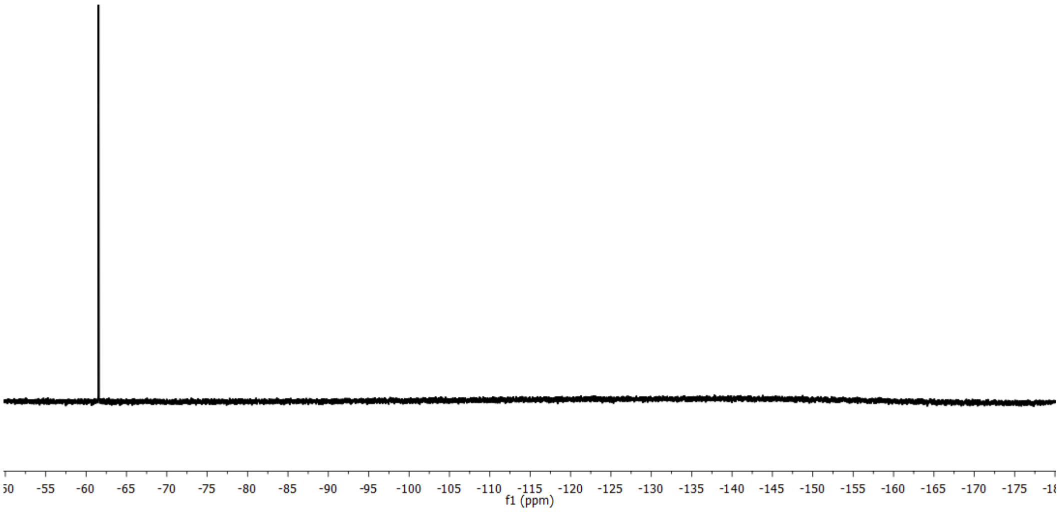

Figure S11: 235 MHz ^{19}F- NMR spectrum of **2** in d_6-DMSO.

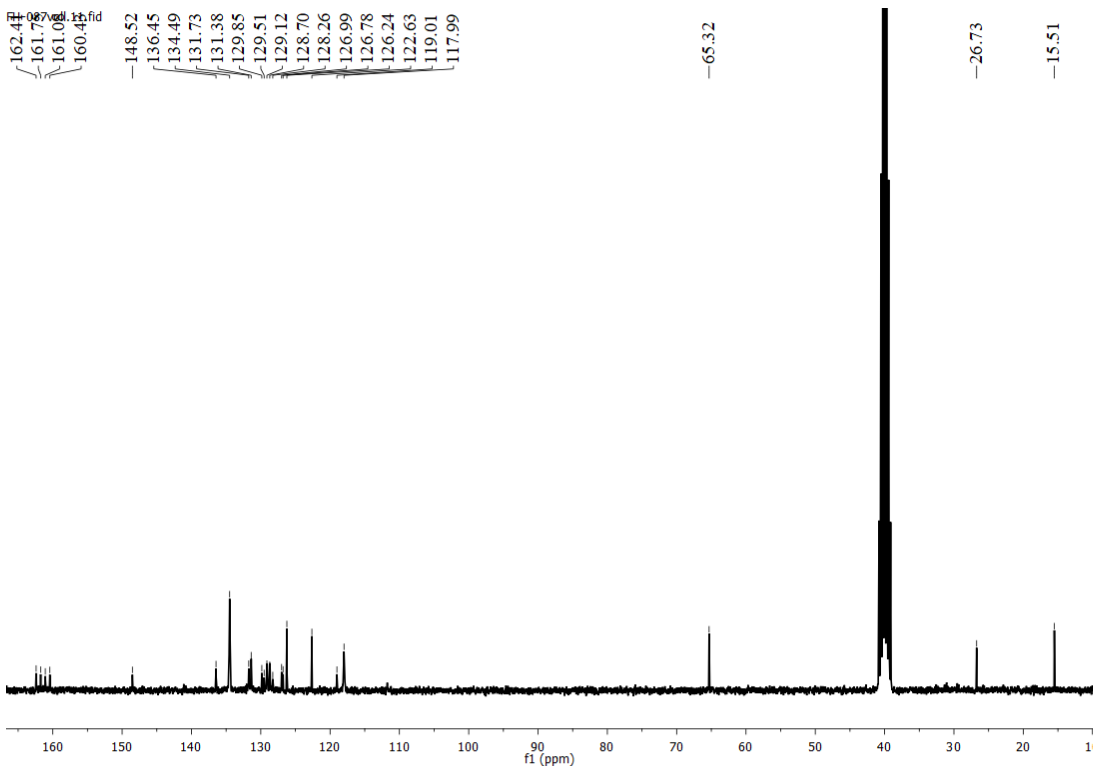

Figure S12: 75 MHz ^{13}C-NMR spectrum of **2** in d_6-DMSO.

2.2 Periodic ^{1}H-NMR spectra of Michael-Addition

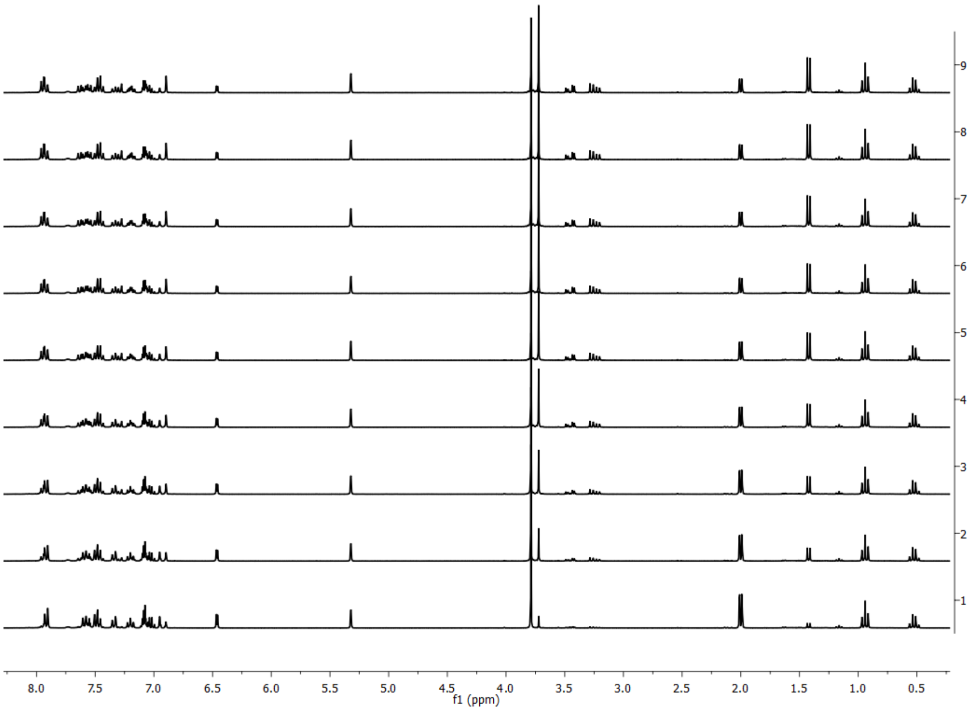

Figure S13: Periodic ^{1}H-NMR of the Michael addition reaction between **8** and **9** with 1 mol-% **2**.

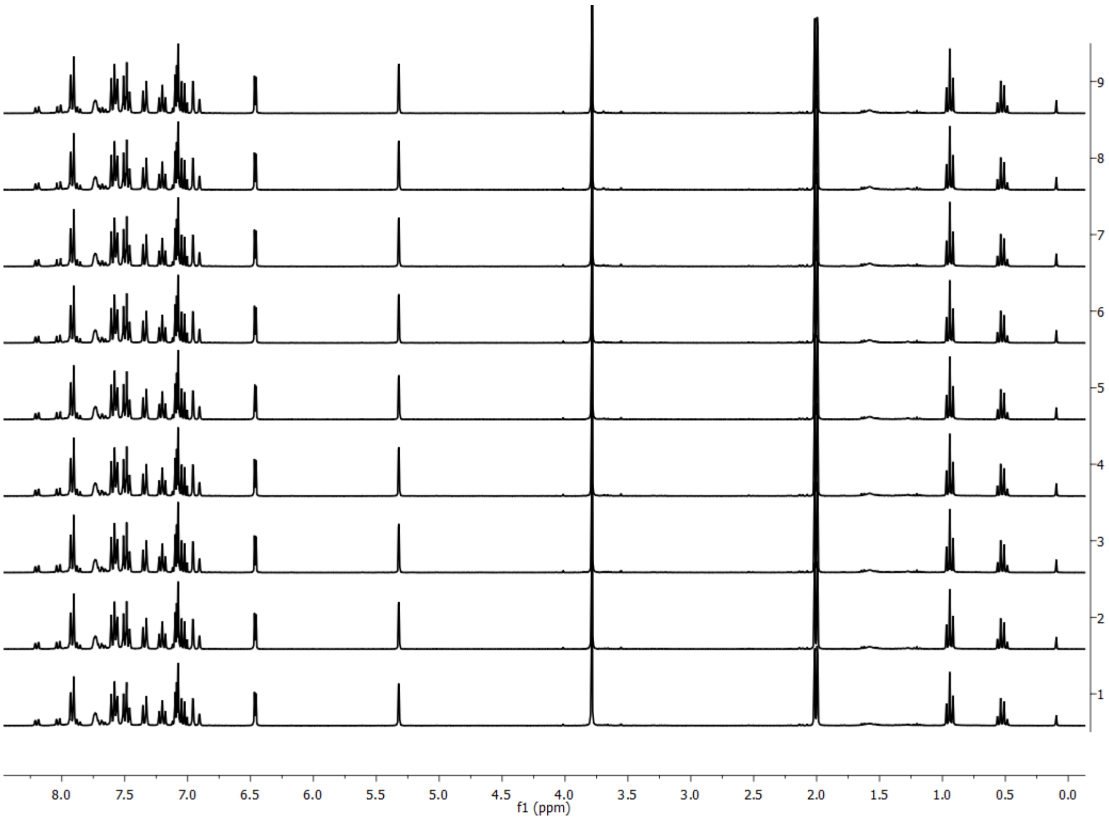

Figure S14: Periodic ^{1}H-NMR of the Michael addition reaction between **8** and **9** with 10 mol-% **3**.

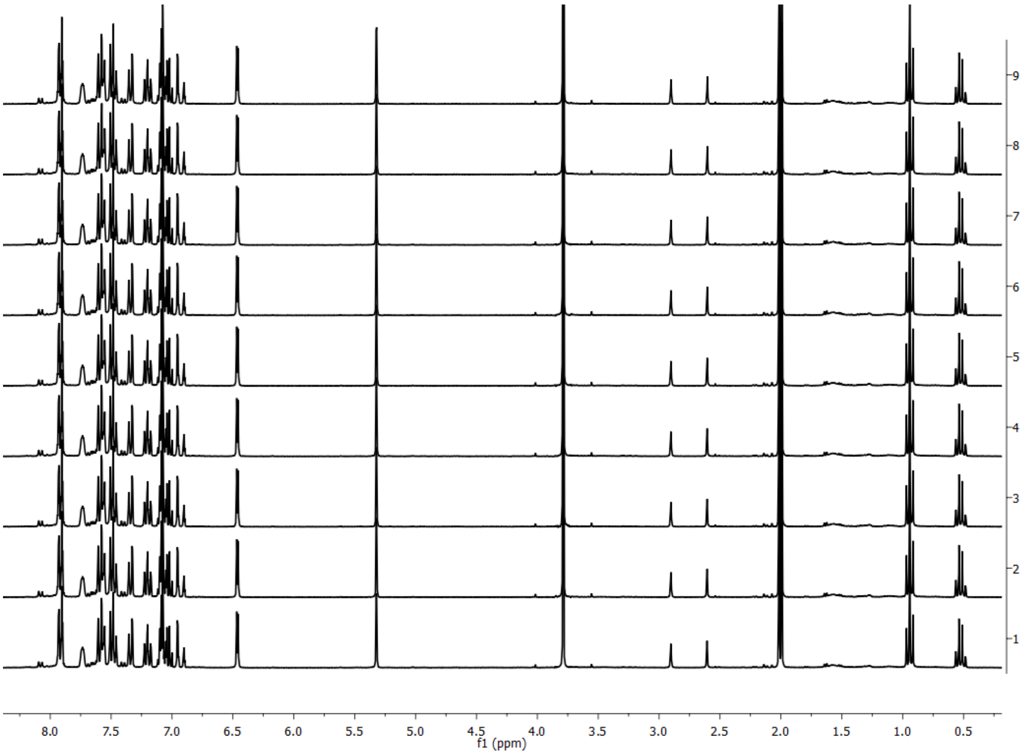

Figure S15: Periodic ^{1}H-NMR of the Michael addition reaction between **8** and **9** with 10 mol-% **4**.

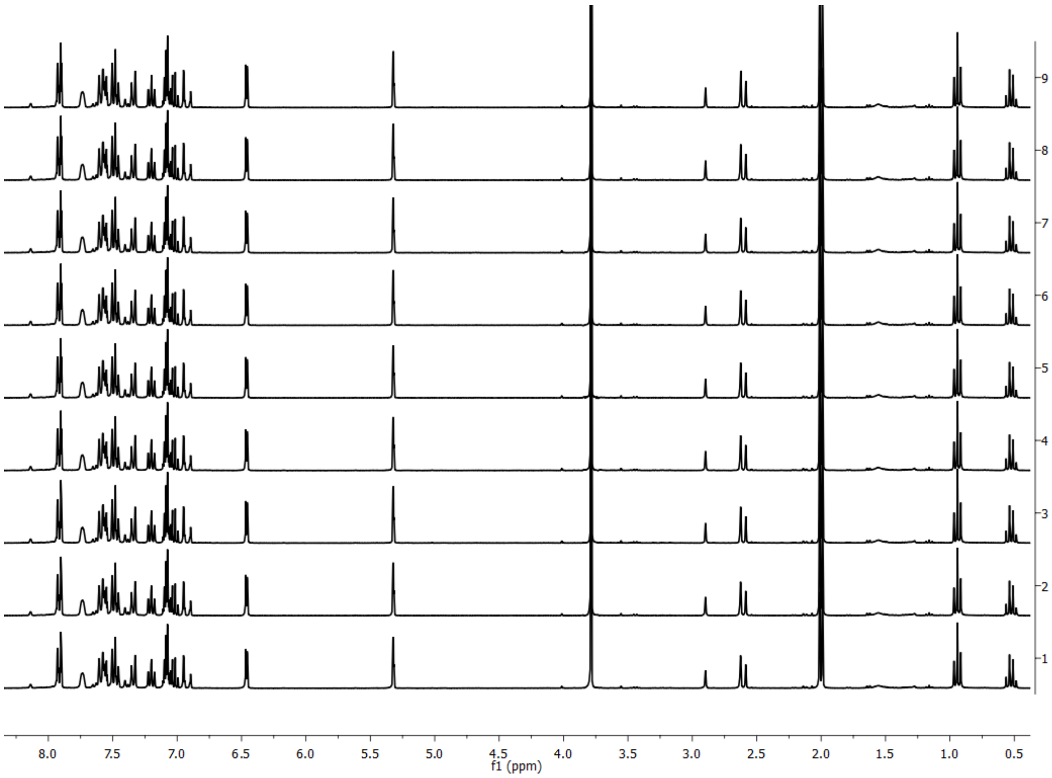

Figure S16: Periodic ^{1}H-NMR of the Michael addition reaction between **8** and **9** with 10 mol-% **5**.

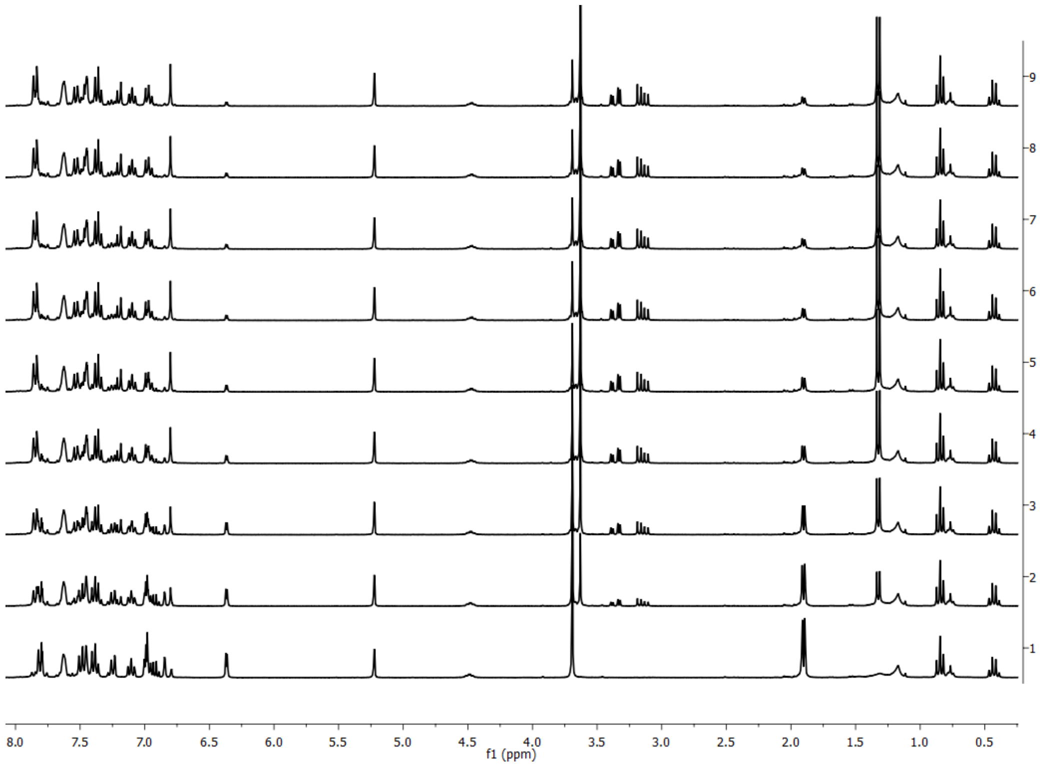

Figure S17: Periodic ^{1}H-NMR of the Michael addition reaction between **8** and **9** with 10 mol-% **6**.

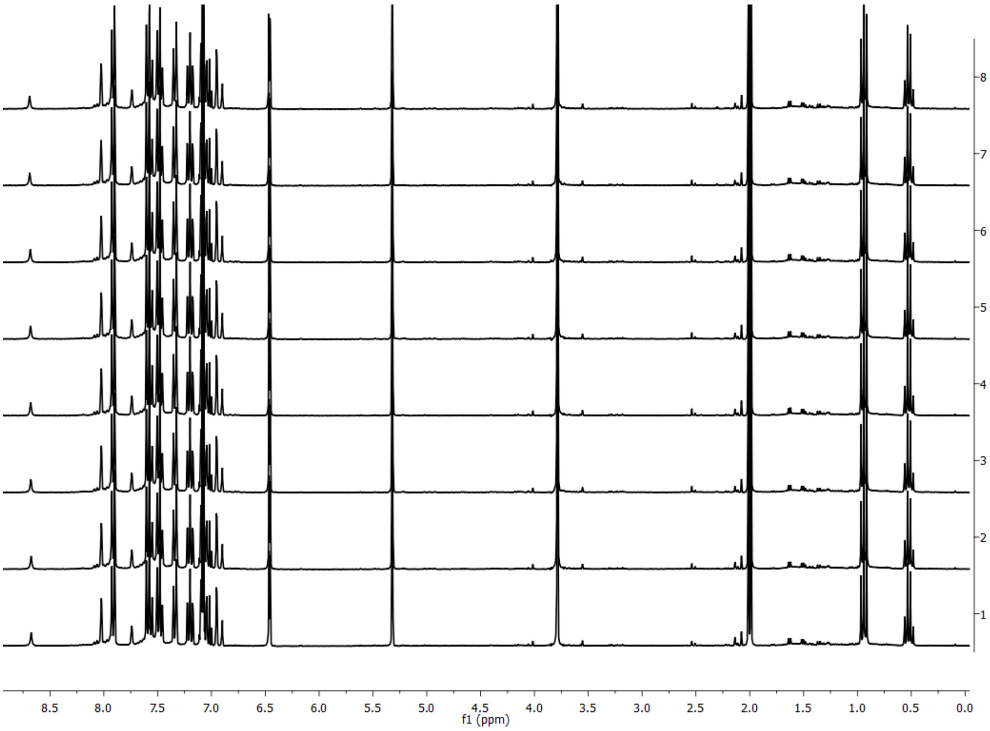

Figure S18: Periodic ^{1}H-NMR of the Michael addition reaction between **8** and **9** with 10 mol-% **7**.

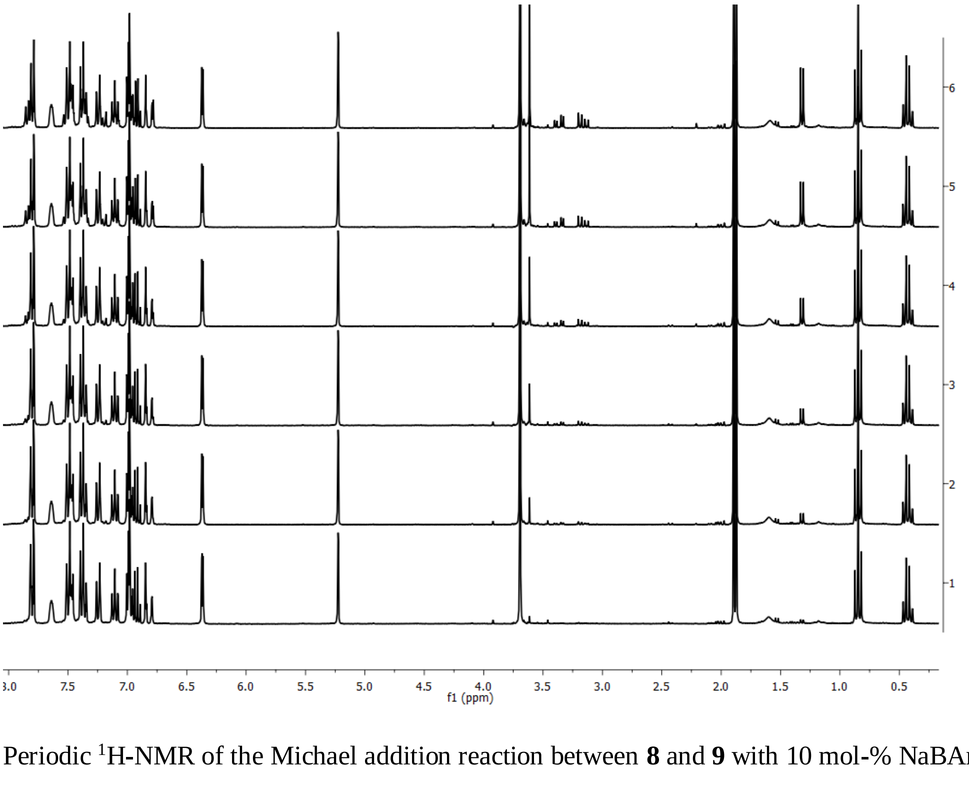

Figure S19: Periodic ^{1}H-NMR of the Michael addition reaction between **8** and **9** with 10 mol-% $NaBAr^{F}_{4}$.

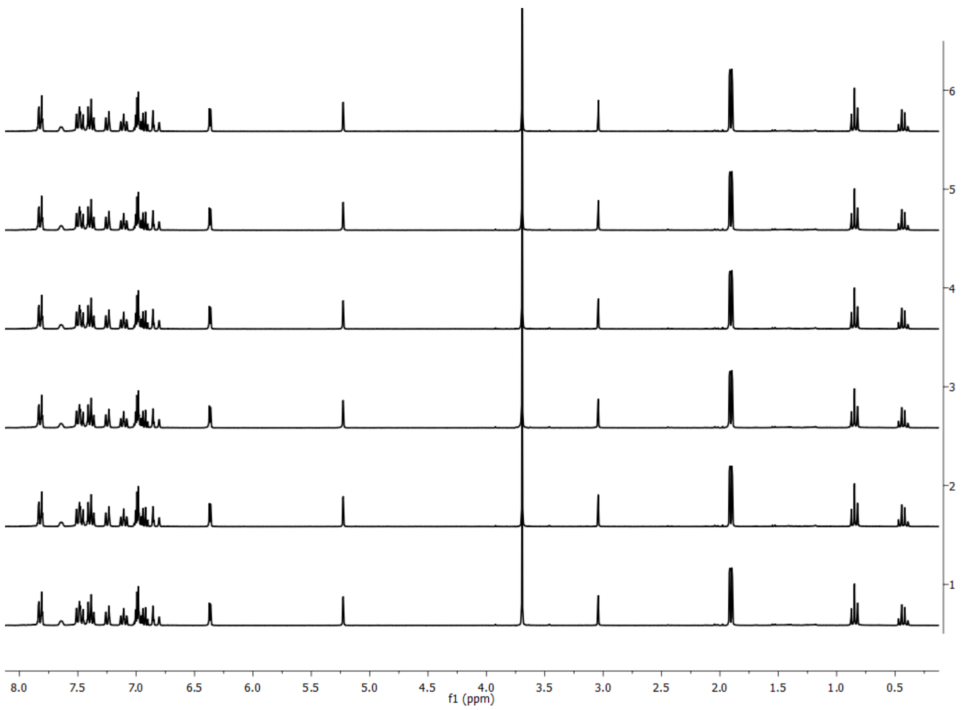

Figure S20: Periodic ^{1}H-NMR of the Michael addition reaction between **8** and **9** with 10 mol-% TMABAr$^F{}_4$.

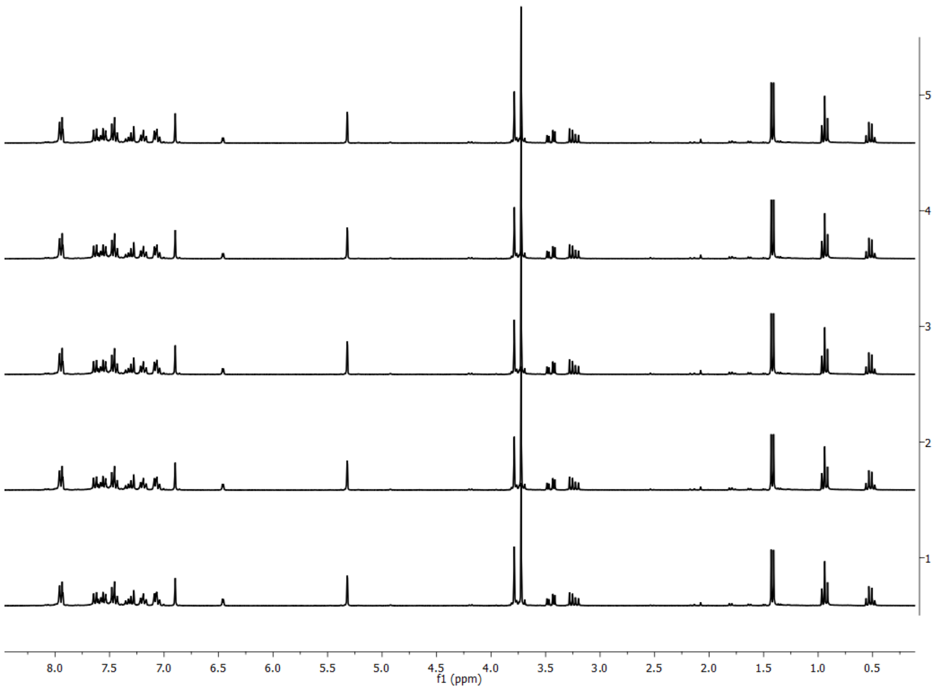

Figure S21: Periodic ^{1}H-NMR of the Michael addition reaction between **8** and **9** with 10 mol-% Iodine.

2.3 Periodic ¹H-NMR spectra of the nitro Michael Addition

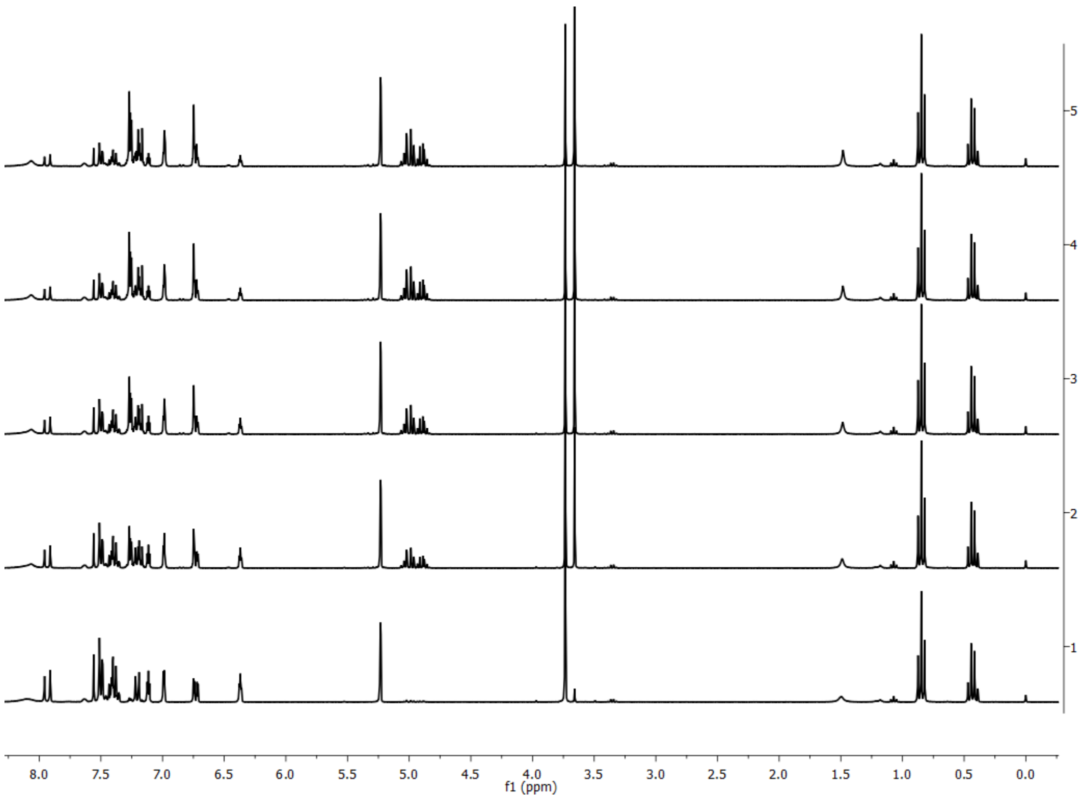

Figure S22: Periodic ¹H-NMR of the nitro Michael addition reaction between **11** and **12** with 1 mol-% **2**.

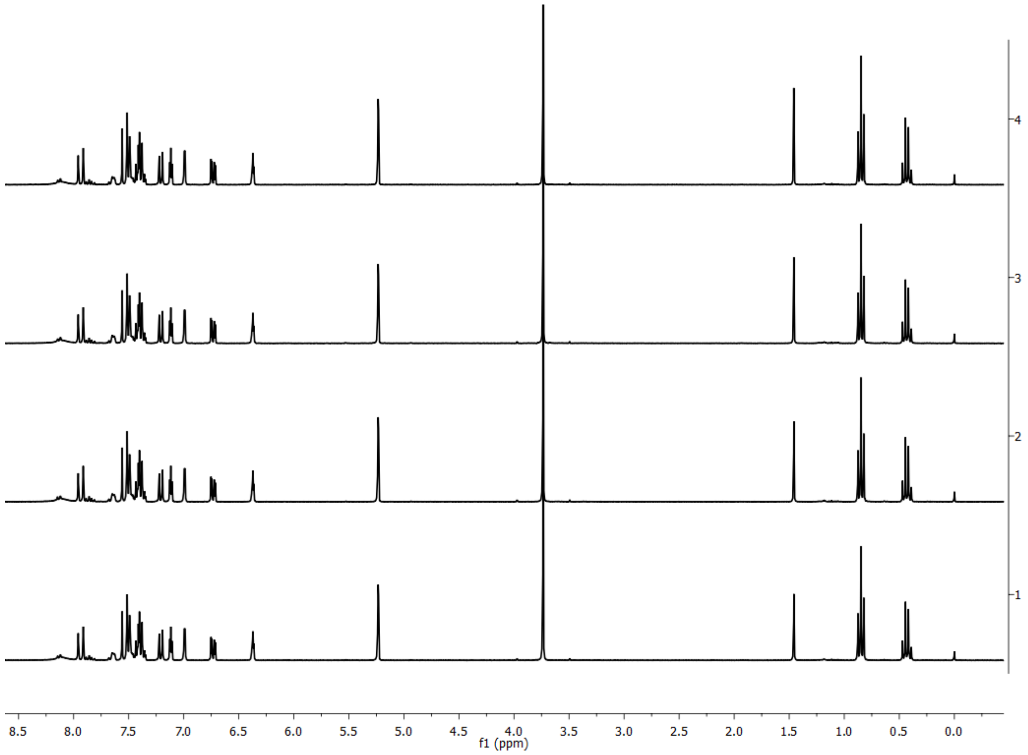

Figure S23: Periodic ¹H-NMR of the nitro Michael addition reaction between **11** and **12** with 10 mol-% **3**.

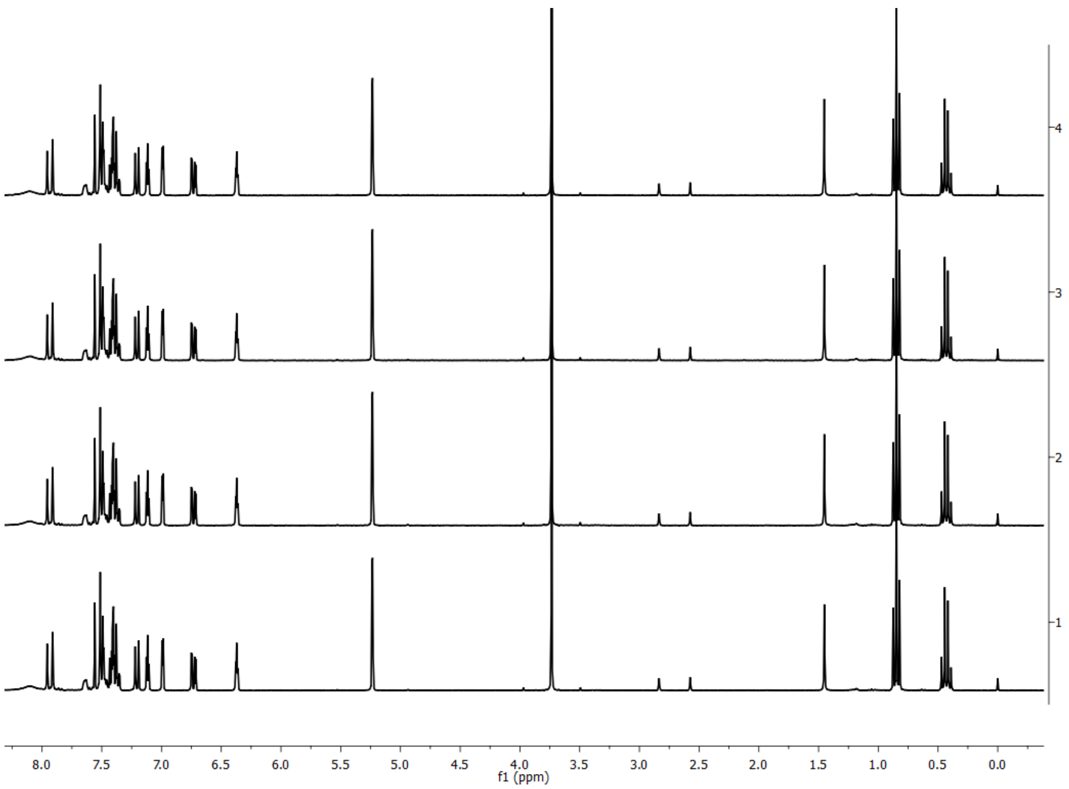

Figure S24: Periodic ^{1}H-NMR of the nitro Michael addition reaction between **11** and **12** with 10 mol-% **4**.

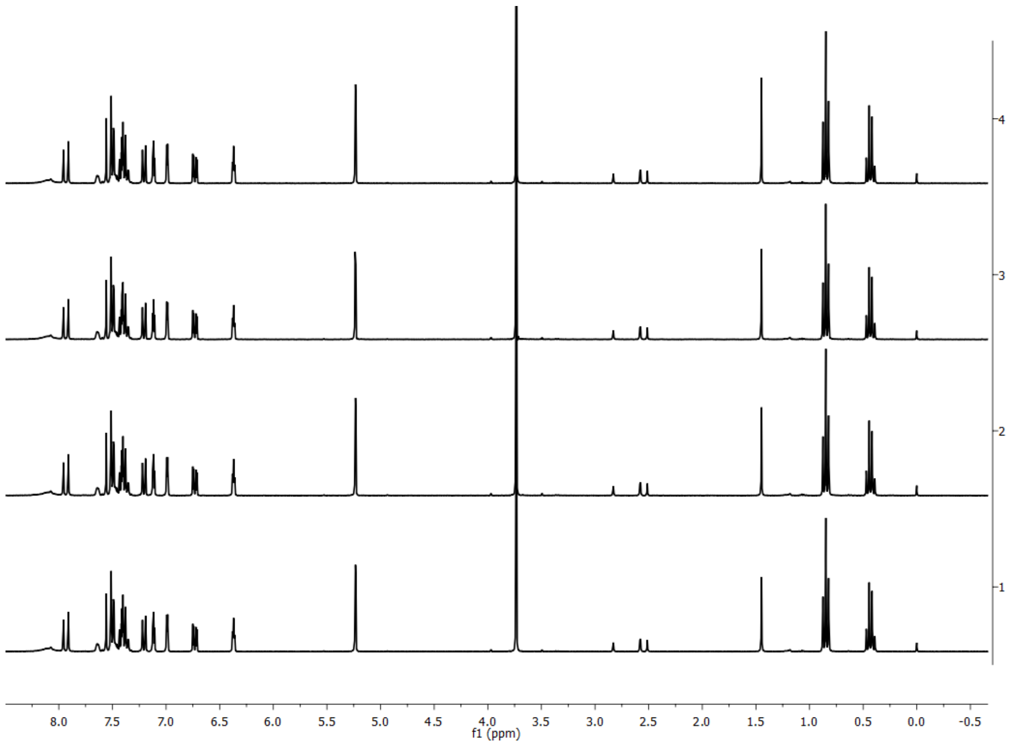

Figure S25: Periodic ^{1}H-NMR of the nitro Michael addition reaction between **11** and **12** with 10 mol-% **5**.

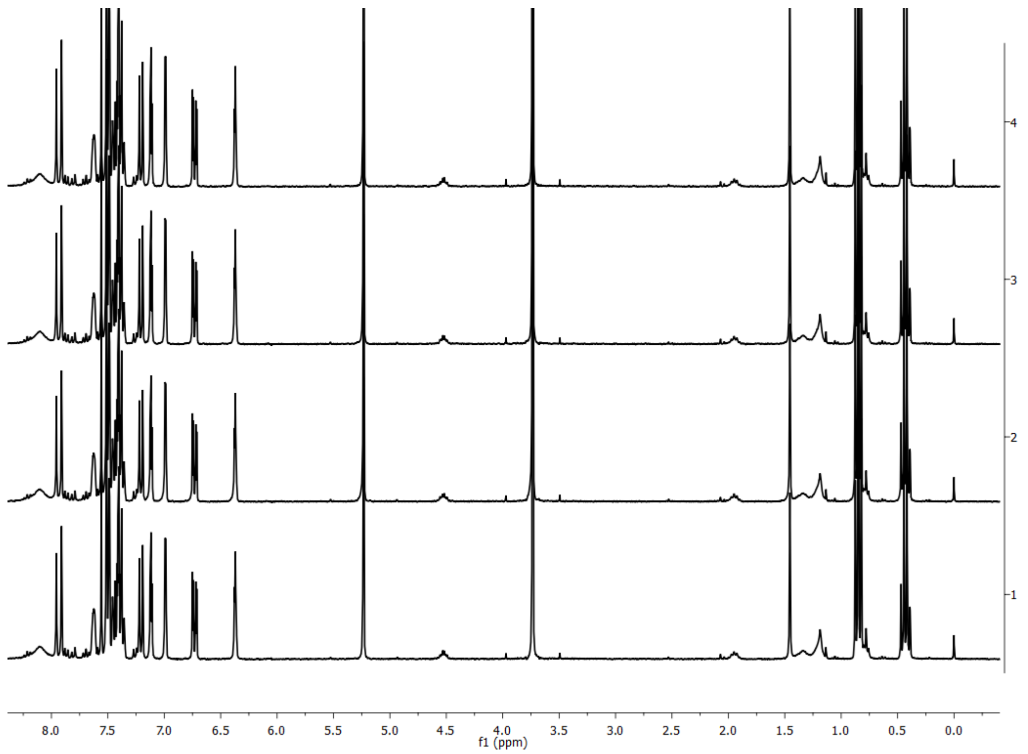

Figure S26: Periodic ^{1}H-NMR of the nitro Michael addition reaction between **11** and **12** with 10 mol-% **6**.

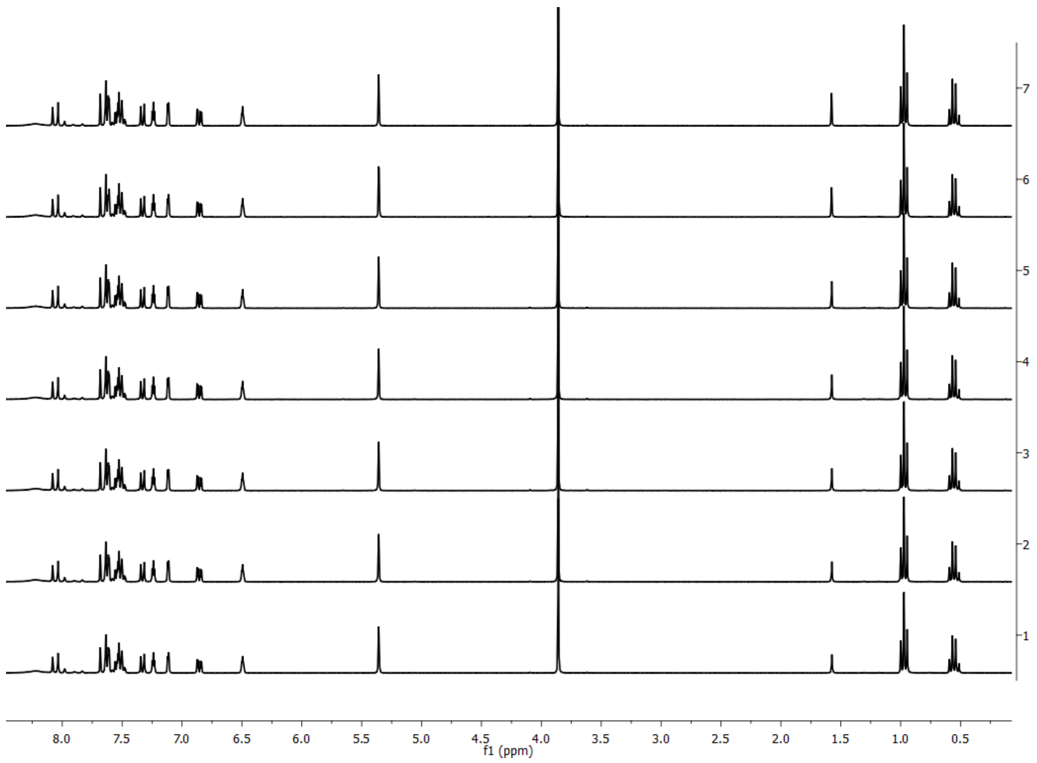

Figure S27: Periodic ^{1}H-NMR of the nitro Michael addition reaction between **11** and **12** with 10 mol-% **7**.

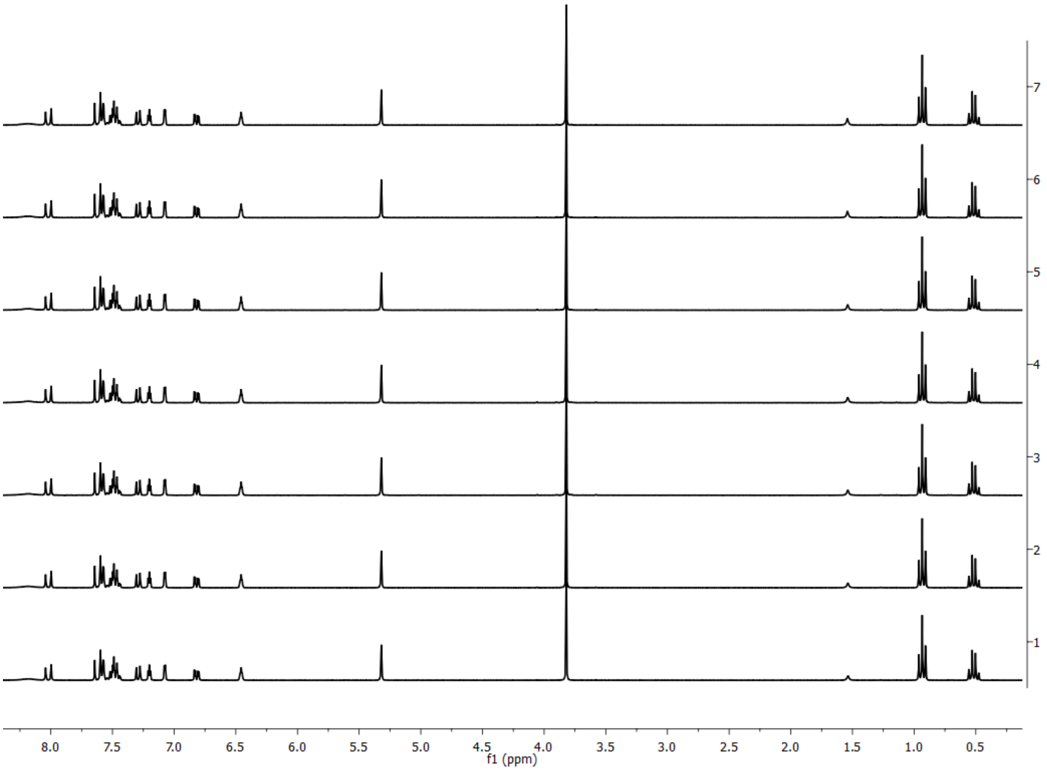

Figure S28: Periodic ^{1}H-NMR of the nitro Michael addition reaction between **11** and **12** with 10 mol-% iodine.

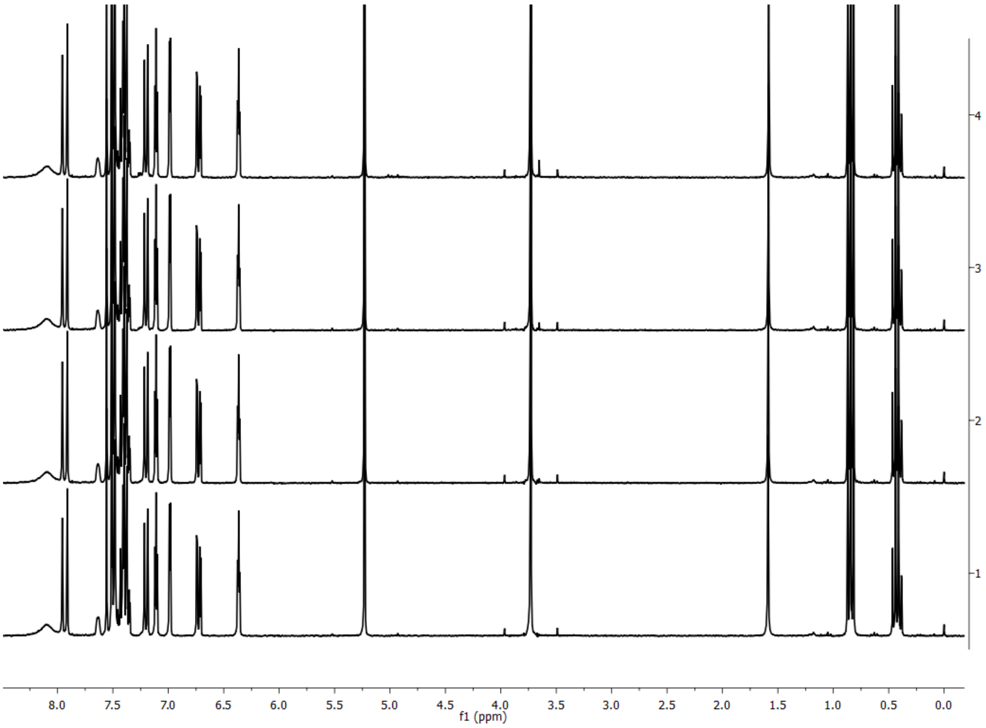

Figure S29: Periodic ^{1}H-NMR of the nitro Michael addition reaction between **11** and **12** with 10 mol-% $NaBAr^{F}_{4}$.

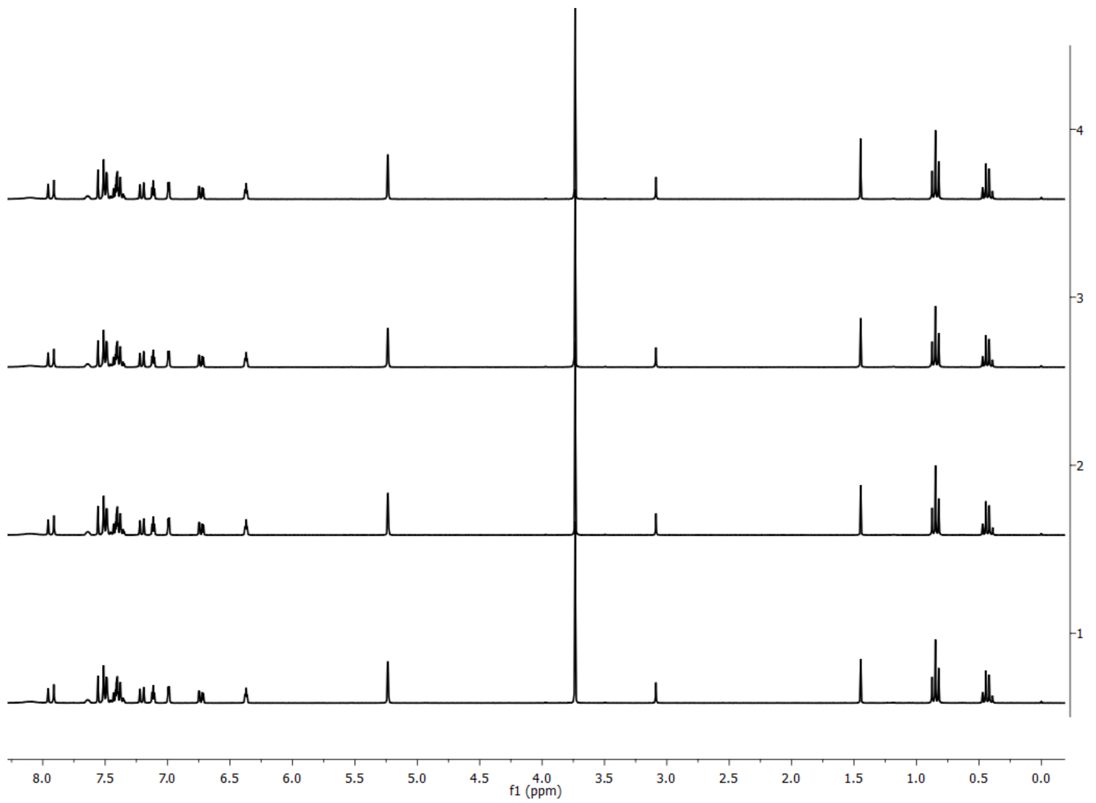

Figure S30: Periodic ^{1}H-NMR of the nitro Michael addition reaction between **11** and **12** with 10 mol-% TMABAr$^{F}_{4}$.

2.4 Periodic ¹H-NMR spectra of the Diels-Alder reaction

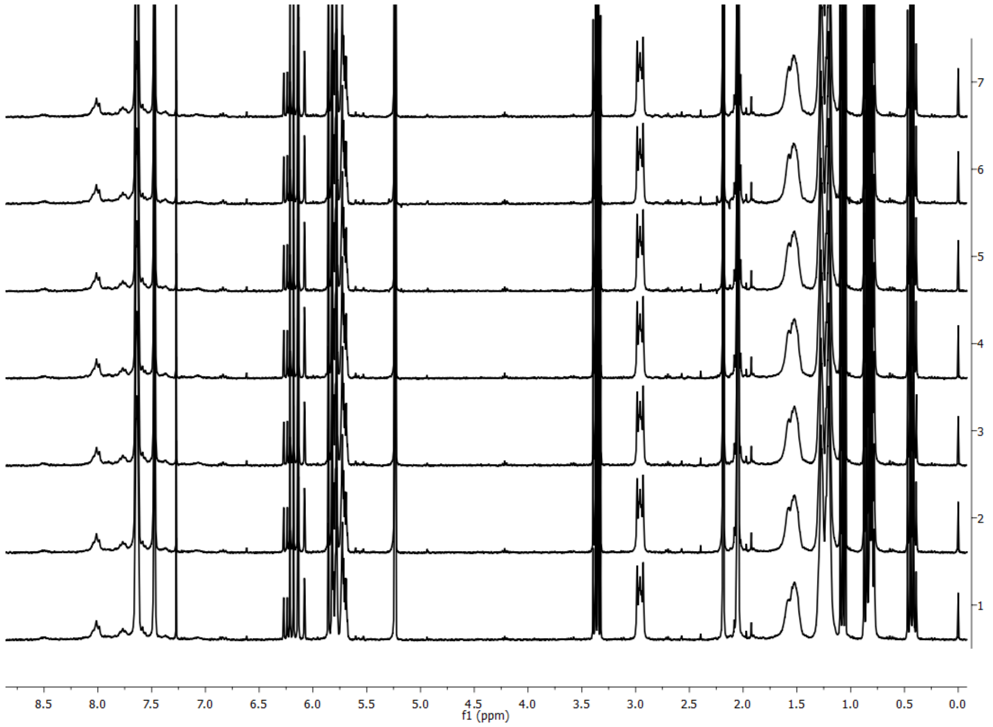

Figure S31: Periodic ¹H-NMR of the Diels-Alder reaction between **14** and **15** with 30 mol-% **2** + 30 mol% TOACl.

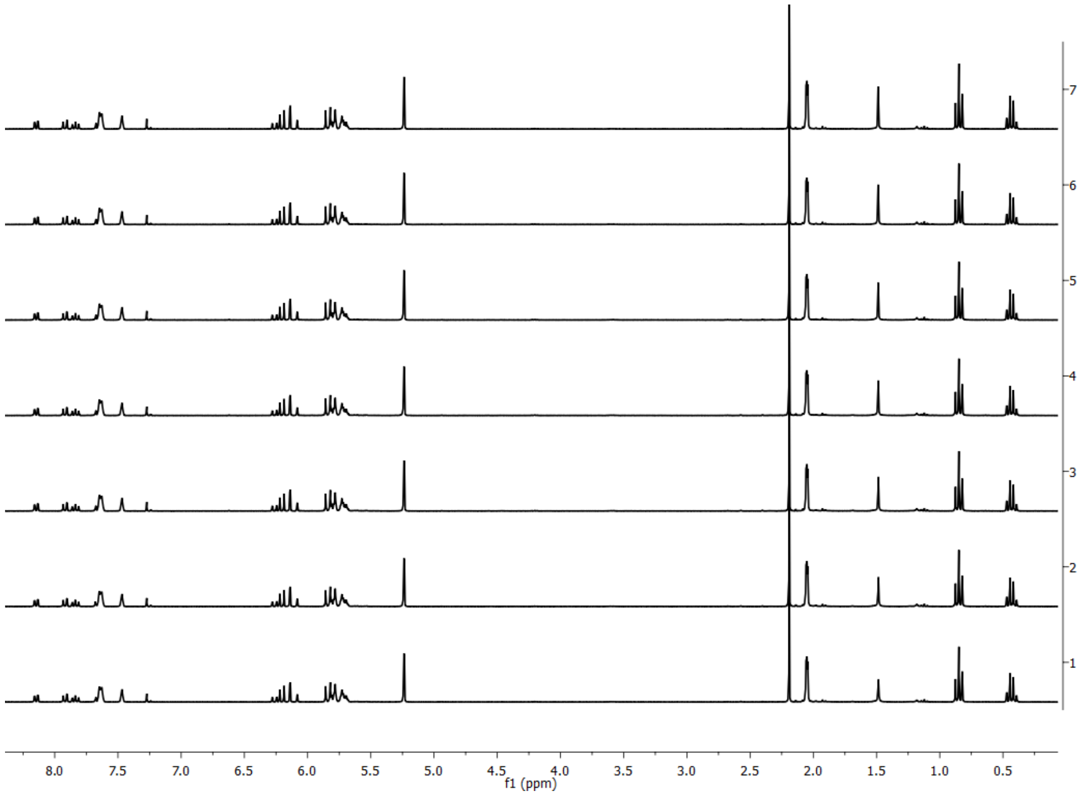

Figure S32: Periodic ¹H-NMR of the Diels-Alder reaction between **14** and **15** with 30 mol-% **3**.

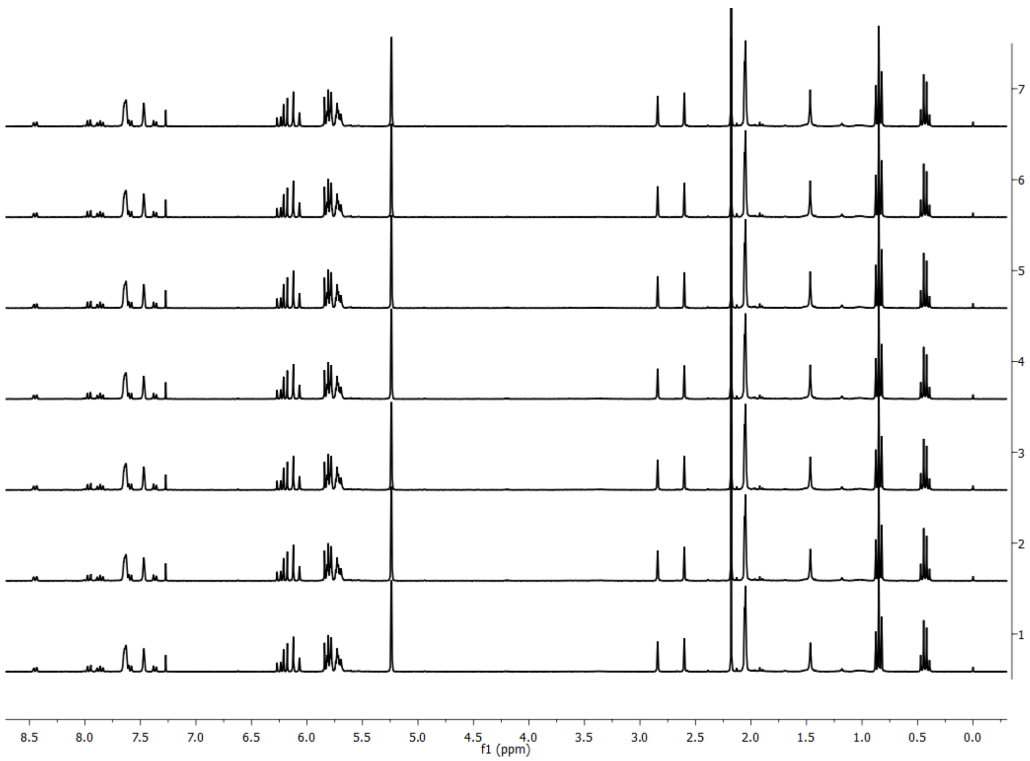

Figure S33: Periodic ^{1}H-NMR of the Diels-Alder reaction between **14** and **15** with 30 mol-% **4**.

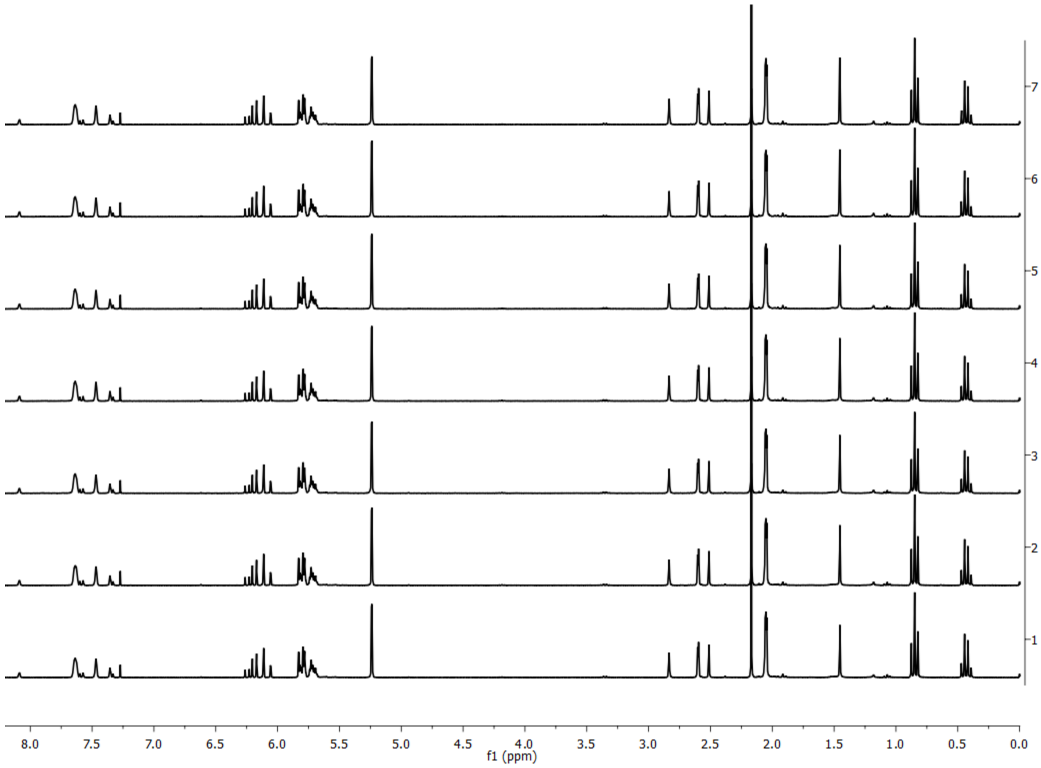

Figure S34: Periodic ^{1}H-NMR of the Diels-Alder reaction between **14** and **15** with 30 mol-% **5**.

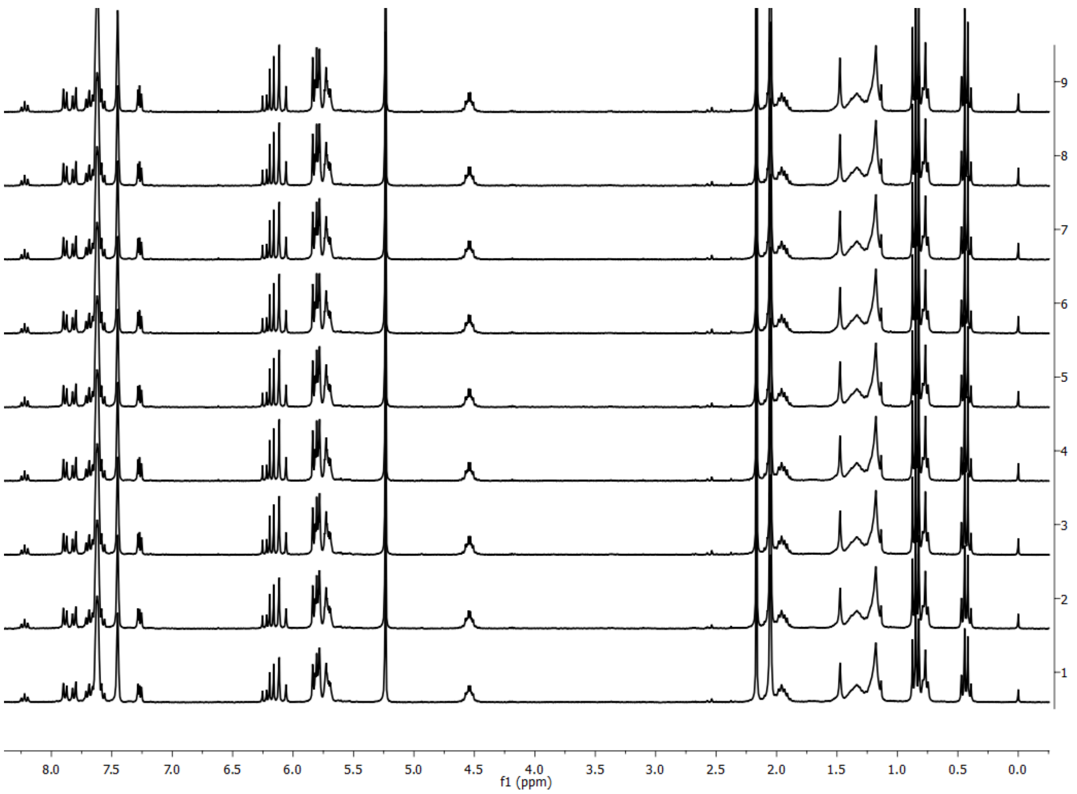

Figure S35: Periodic ^{1}H-NMR of the Diels-Alder reaction between **14** and **15** with 30 mol-% **6**.

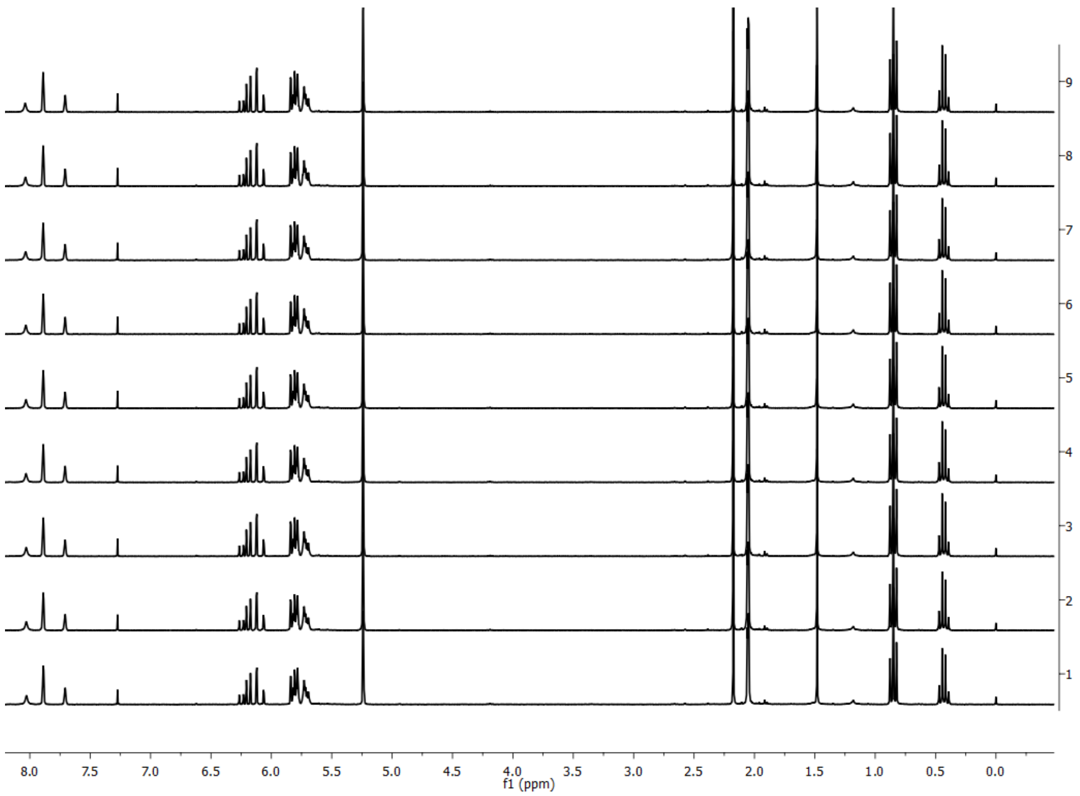

Figure S36: Periodic ^{1}H-NMR of the Diels-Alder reaction between **14** and **15** with 30 mol-% **7**.

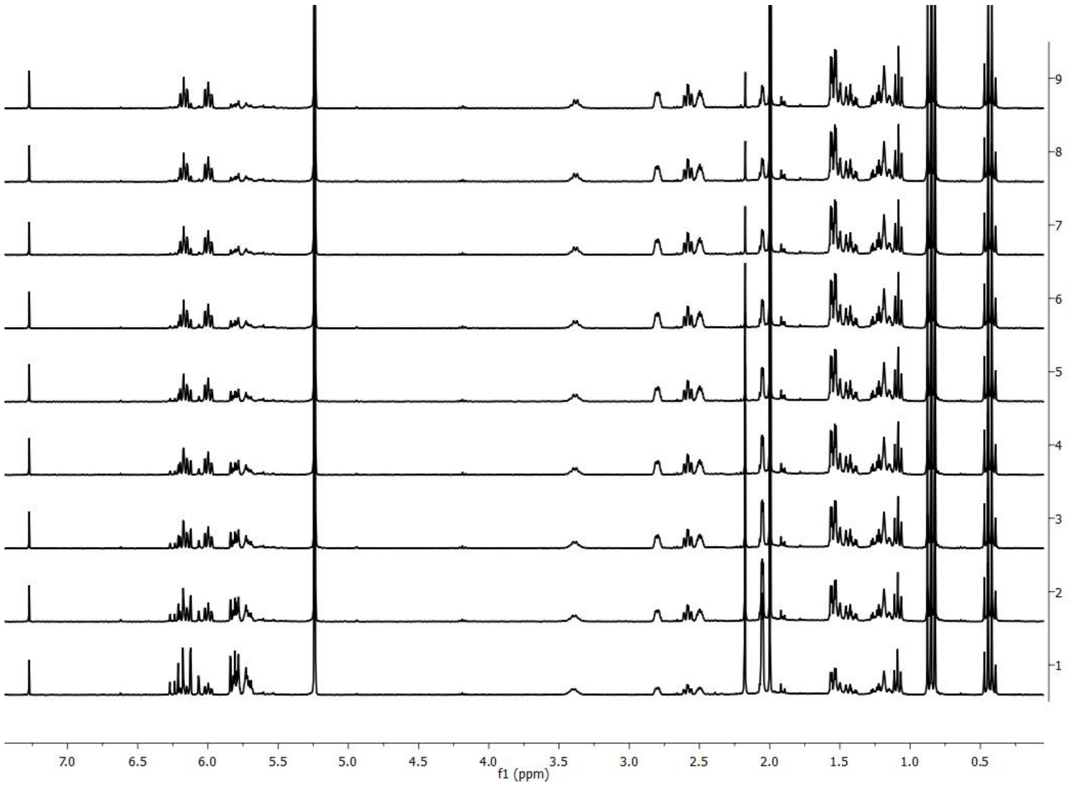

Figure S37: Periodic ^{1}H-NMR of the Diels-Alder reaction between **14** and **15** with 30 mol-% BF_3•Etherate.

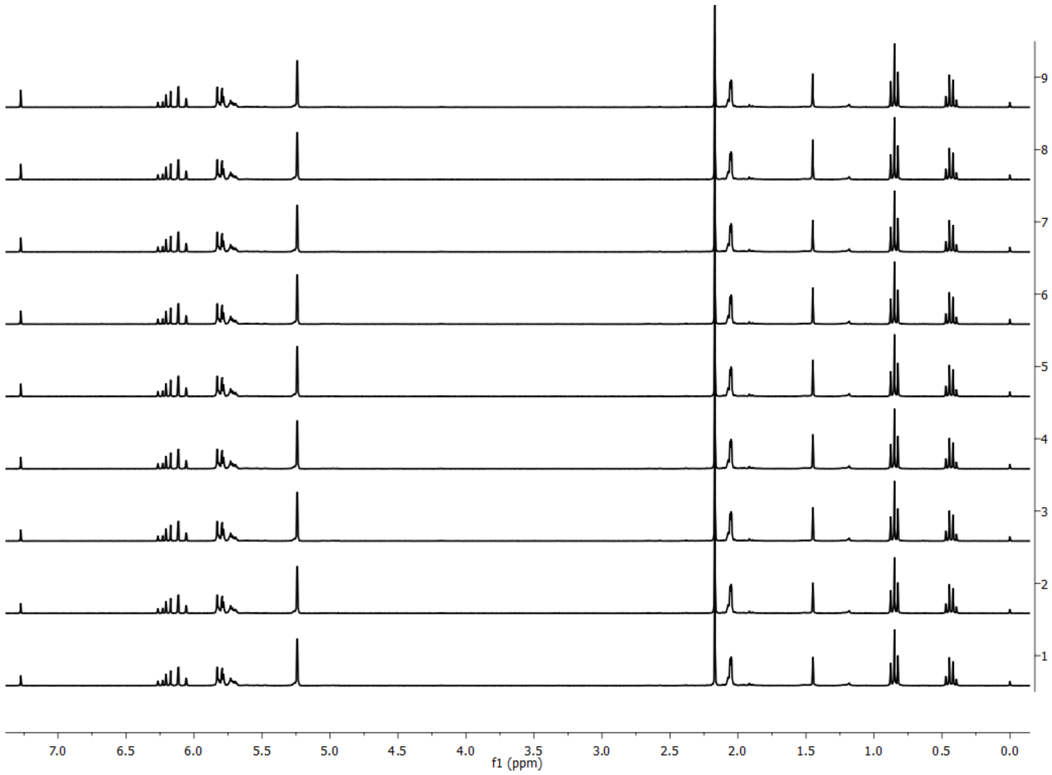

Figure S38: Periodic ^{1}H-NMR of the Diels-Alder reaction between **14** and **15** with 30 mol-% iodine.

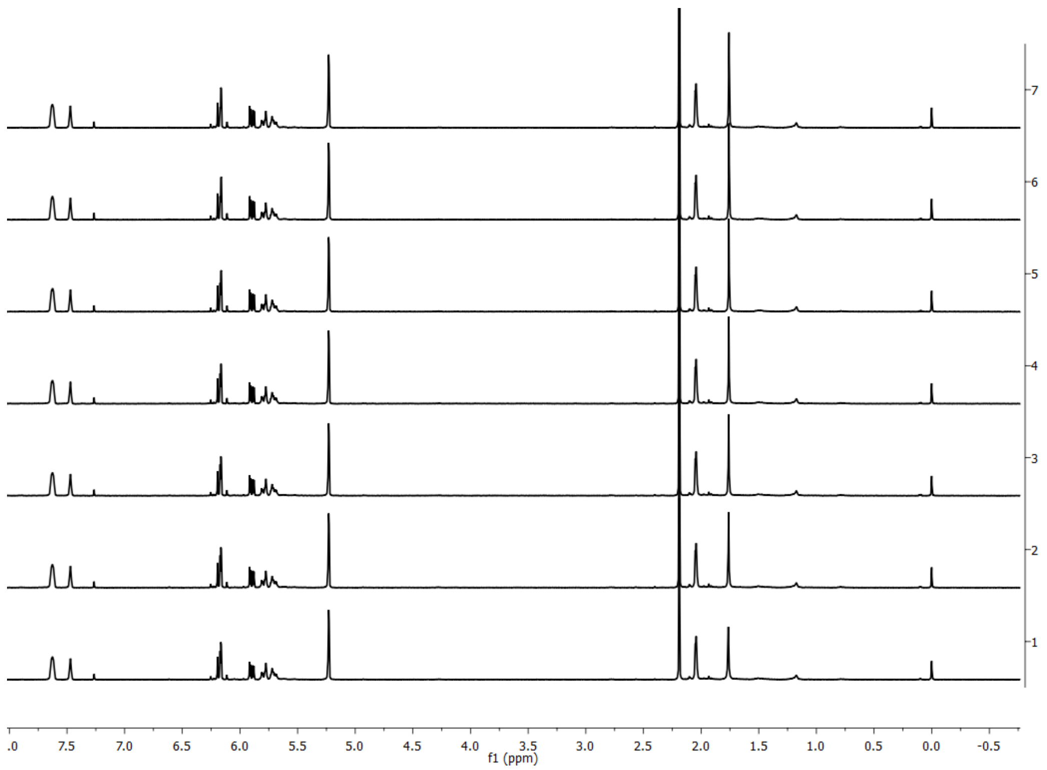

Figure S39: Periodic ^{1}H-NMR of the Diels-Alder reaction between **14** and **15** with 30 mol-% $NaBAr^{F}_{4}$.

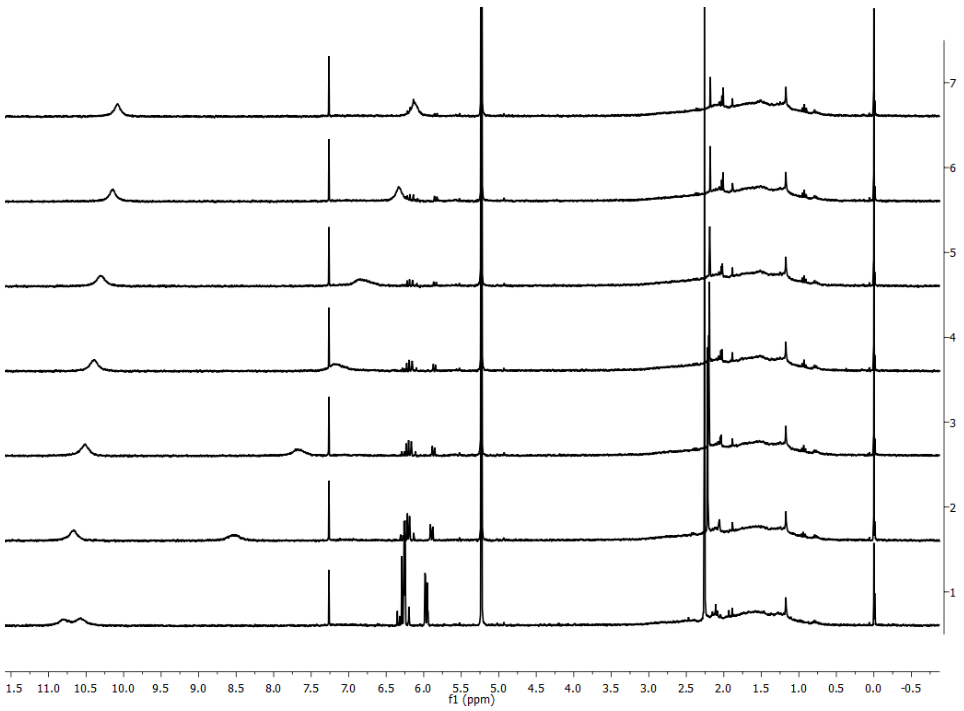

Figure S40: Periodic ^{1}H-NMR of the Diels-Alder reaction between **14** and **15** with 5 mol-% HOTf. Cyclohexadiene is at the first measurement point already decomposed and methyl vinyl ketone is also being degraded.

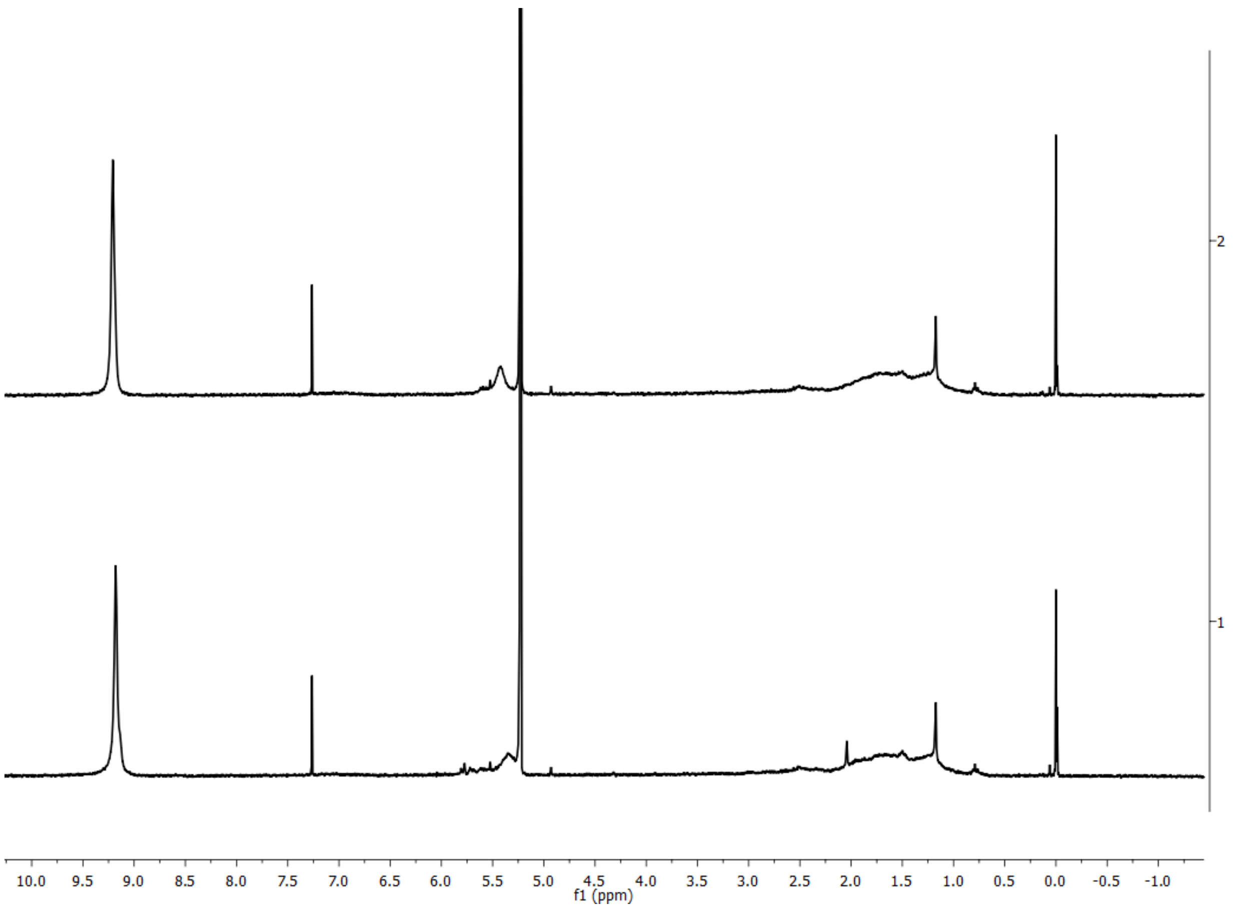

Figure S41: Cyclohexadiene **14** and 5 mol-% HOTf. After 5 minutes (bottom) only traces of **14** detectable and after 15 minutes complete degradation occurred (top).

3. DFT Calculations

3.1 Method

Orientating density functional (DFT) calculations were performed with the Gaussian16 suite of programs (Revision B.01).[S10] The M06-2X density functional was used, which is recommended for weak non-covalent interactions.[S11] The basis set (def2-TZVP(D))[S12] was of triple-zeta quality, and the corresponding pseudopotential for iodine was employed.[S13] The nature of the obtained minima and transition state structures was confirmed by the expected number of imaginary frequencies (N_{imag}=0 and N_{imag}=1). Grimme's low-frequency entropy corrections[S14] have been applied to all Gibbs free energies. In all computations, the non-coordinating counterions were omitted.

3.2 Data

Energy profile of the Michael addition reaction

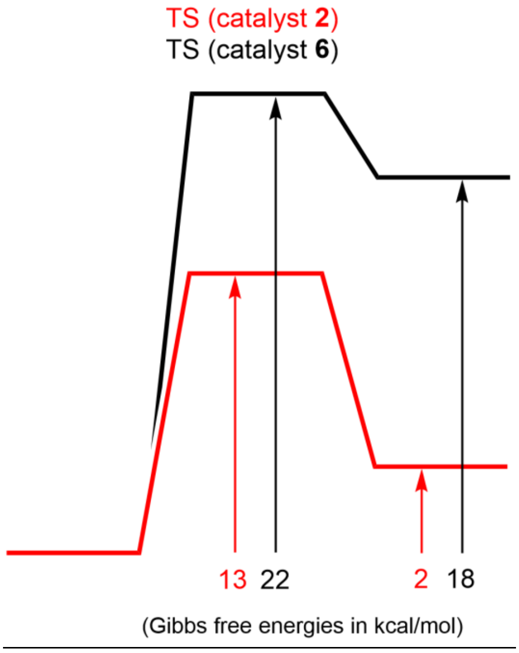

Transition state of the Michael addition reaction involving catalyst **2**

E (hartree) = -2472.650480

G (hartree) = -2472.191412

C	-2.81234900	-1.82724800	-0.39129400
H	-1.96719900	-1.89806200	-1.06582900
C	-2.57026600	-1.25364300	0.87105100
H	-3.31190700	-1.39341900	1.64524200
O	-0.53234200	-0.28349000	0.24451100

C	-3.87368600	-2.87497500	-0.51157800
C	-5.82449900	-0.20815300	-1.34000000
C	-4.65889200	0.31381100	-0.76297600
C	-4.75907300	1.07825100	0.39678500
C	-6.01490600	1.28899000	0.94074300
C	-7.16432900	0.74966700	0.35004000
C	-7.08947300	-0.01024500	-0.80419800
C	-4.13038500	-0.85061500	-2.63251600
C	-3.54617800	-0.16723000	-1.55378800
H	-3.87863600	1.51470000	0.85240500
H	-8.12962000	0.93710600	0.79983800
H	-7.97813100	-0.42314000	-1.26299700
H	-2.55836300	0.26527700	-1.58747400
H	-3.65032700	-1.32549100	-3.47566400
N	-5.45250900	-0.91809600	-2.48570300
H	-4.85132800	-2.47070600	-0.23668500
H	-3.93034400	-3.30097100	-1.51044000
H	-3.65603300	-3.67960100	0.19486300
C	-1.46001000	-0.47228100	1.13944600
C	-1.26943200	0.15123000	2.46982200
C	0.02381800	0.42782600	2.92328300
C	-2.35299800	0.46616600	3.29459500
C	0.23132000	0.99085300	4.17143600
H	0.87712800	0.17515200	2.30450700
C	-2.14513100	1.04188100	4.53713600
H	-3.36446500	0.28009800	2.95690400
C	-0.85459300	1.30228100	4.97973300
H	1.23771700	1.18139400	4.51968100
H	-2.99195200	1.28858800	5.16328000
H	-0.69513000	1.74549500	5.95365100
C	4.01181500	-2.32374100	-0.33456500
C	2.97769700	-3.20474500	-0.00290900
C	3.16388000	-4.56106400	0.16499900
C	4.45051400	-5.05658600	-0.00946300
C	5.50135400	-4.20633400	-0.34104900
C	5.29211500	-2.84777000	-0.50406700
C	3.61688500	-0.93489200	-0.46087000
H	2.35079500	-5.22585300	0.42419600
H	4.63091300	-6.11557700	0.11602900
H	6.49526200	-4.61172800	-0.47217600

H	6.11657600	-2.19370100	-0.76038900
C	2.31245500	-0.57706800	-0.25413200
C	1.90048300	3.49434800	-0.77252900
C	3.16667200	2.92527800	-0.94157000
C	4.22506900	3.76649800	-1.28036200
C	3.99658400	5.12257700	-1.43702300
C	2.72389800	5.65798600	-1.26057000
C	1.65069200	4.84265000	-0.92230300
C	2.09012400	0.80203900	-0.40947100
C	3.22560400	1.49148700	-0.73960400
H	5.21902700	3.35912600	-1.41951600
H	4.81837500	5.77457100	-1.69957400
H	2.56210800	6.71997100	-1.38618800
H	0.66382300	5.26376600	-0.78479400
S	4.57886300	0.43560400	-0.85470000
I	0.46806100	2.04568500	-0.25008400
I	1.14045100	-2.19865400	0.19981000
C	-6.38733700	-1.62452500	-3.34461600
H	-7.13096800	-0.92698300	-3.72750400
H	-5.84340900	-2.06317700	-4.17669600
H	-6.88741500	-2.41167400	-2.78064000
H	-6.11780700	1.89092900	1.833848

<u>Starting material complex of the Michael addition reaction involving catalyst **2**</u>

E (hartree) = -2472.670034

G (hartree) = -2472.212729

C	-1.87750100	-2.98683500	0.42051100
H	-1.09825500	-2.58751700	-0.22517500
C	-2.48111700	-2.15995200	1.28228100
H	-3.31365600	-2.50479900	1.88288900
O	-0.95453100	-0.35545800	1.12209500
C	-2.23285400	-4.41850600	0.24800400
C	-2.36118700	-2.30032000	-2.57704900
C	-2.01784400	-0.98776800	-2.17858800
C	-3.04523400	-0.12737500	-1.76710200
C	-4.34392400	-0.59021200	-1.74320400
C	-4.65759000	-1.90298500	-2.13637400
C	-3.67565000	-2.76953400	-2.56765500
C	-0.15639800	-2.08969200	-2.79248200
C	-0.59526200	-0.88141700	-2.32293300

H	-2.83977200	0.90296400	-1.50600000
H	-5.68912300	-2.22819100	-2.12756400
H	-3.92163700	-3.77006600	-2.90120400
H	0.02394100	-0.01015100	-2.18391800
H	0.83696300	-2.40108600	-3.07616300
N	-1.20781200	-2.95438100	-2.93655400
H	-2.59316100	-4.58679800	-0.76856300
H	-1.34864000	-5.04772600	0.37670600
H	-3.00074700	-4.73731700	0.94944200
C	-2.09496300	-0.76034700	1.43123500
C	-3.06999200	0.18128800	2.00846600
C	-2.62250100	1.24063100	2.80420400
C	-4.43725800	0.01616300	1.77570800
C	-3.53191500	2.12853400	3.35214800
H	-1.56768400	1.32634600	3.03576800
C	-5.34092900	0.92295000	2.30402400
H	-4.78343900	-0.80129300	1.15574200
C	-4.88985100	1.97475100	3.09253500
H	-3.19059800	2.92748900	3.99665300
H	-6.39868100	0.80552500	2.11132200
H	-5.60080500	2.66901000	3.52097300
C	4.11181500	-1.03907800	0.40439200
C	3.42720300	-2.09335900	1.01399400
C	4.02996100	-3.26718600	1.41453500
C	5.39634400	-3.38591400	1.19067100
C	6.11112000	-2.35655500	0.58443300
C	5.48154400	-1.18828600	0.19132100
C	3.30001400	0.11347700	0.07335300
H	3.47858300	-4.06793900	1.88845900
H	5.90446800	-4.29074700	1.49556000
H	7.17400300	-2.46979600	0.42040200
H	6.04794700	-0.39230000	-0.27653100
C	1.95862900	0.12069700	0.35444100
C	0.37682100	3.77995000	-0.76247300
C	1.73318200	3.53331700	-0.98925100
C	2.48773800	4.54278400	-1.58513800
C	1.88001200	5.73789300	-1.92885300
C	0.52493200	5.94720200	-1.68825000
C	-0.25324600	4.96031100	-1.09509700
C	1.33261700	1.32969500	-0.00678500

C	2.20124000	2.22931100	-0.56822600
H	3.54275100	4.39022900	-1.77737500
H	2.46661500	6.51980500	-2.39132800
H	0.06543500	6.88679200	-1.96366000
H	-1.30572700	5.12638800	-0.90946300
S	3.79405600	1.59102000	-0.65048800
I	-0.55482100	2.13262900	0.14424900
I	1.38446300	-1.66024400	1.20841900
C	-1.15533000	-4.26774200	-3.54840500
H	-1.65303500	-4.25816100	-4.51845400
H	-0.11523500	-4.55155900	-3.68744100
H	-1.63766700	-5.00832900	-2.91061500
H	-5.14359200	0.07548700	-1.44530000

Product complex of the Michael addition reaction involving catalyst **2**

E (hartree) = -2472.668177

G (hartree) = -2472.206165

C	-2.91844100	-1.70655200	-0.15166600
H	-2.04522000	-2.06815500	-0.70190400
C	-2.48384800	-1.11286200	1.15428100
H	-3.12837300	-1.28136700	2.00723300
O	-0.49211900	-0.21663600	0.30747300
C	-3.90334100	-2.85163300	0.05967800
C	-5.81013200	-0.17874800	-1.44462300
C	-4.77581400	0.07830300	-0.55223700
C	-5.04473000	0.82369300	0.58152000
C	-6.34819400	1.27014500	0.78215000
C	-7.36523600	0.98683700	-0.12636700
C	-7.11109000	0.24943000	-1.27583800
C	-4.01147700	-1.14177300	-2.35582600
C	-3.52752900	-0.57768000	-1.07100300
H	-4.26249900	1.05864100	1.29027100
H	-8.36646800	1.34940900	0.06102000
H	-7.89418900	0.03189400	-1.98970800
H	-2.71055300	0.12880900	-1.25525600
H	-3.43690400	-1.70779800	-3.07831500
N	-5.27120600	-0.93476500	-2.53086200
H	-4.81398100	-2.49703800	0.54630800
H	-4.17988100	-3.34055300	-0.87667400
H	-3.45958700	-3.60711300	0.70702100

C	-1.36236100	-0.38411500	1.31540100
C	-0.99899000	0.22760500	2.62367400
C	0.34098500	0.43657200	2.95509200
C	-1.97511600	0.60130600	3.54928500
C	0.69665600	0.98541200	4.17840100
H	1.12232800	0.15415500	2.25945100
C	-1.62245100	1.15448200	4.76953200
H	-3.02283800	0.46668800	3.31288400
C	-0.28490200	1.34686000	5.08967200
H	1.74123000	1.12613800	4.42308900
H	-2.39345000	1.44054100	5.47250600
H	-0.01037300	1.77745200	6.04318100
C	3.86681800	-2.39412600	-0.49879700
C	2.82124600	-3.22066500	-0.07140700
C	2.97509000	-4.57896900	0.11838500
C	4.22616900	-5.13045000	-0.13062900
C	5.28221400	-4.33125900	-0.55823700
C	5.11183100	-2.97001900	-0.74334700
C	3.51682100	-0.99252500	-0.62963800
H	2.15912800	-5.20550000	0.45388900
H	4.37594000	-6.19195100	0.01274900
H	6.24873500	-4.77825300	-0.74648000
H	5.93932300	-2.35429400	-1.07414400
C	2.24198700	-0.59274800	-0.34435100
C	1.89732200	3.47491300	-0.80726000
C	3.13739400	2.88529300	-1.07796500
C	4.18709600	3.70682300	-1.48420000
C	3.97928500	5.06976200	-1.60710600
C	2.73578900	5.62976000	-1.32925200
C	1.67341400	4.83175500	-0.92259900
C	2.05215500	0.78911300	-0.48868400
C	3.17967700	1.44790000	-0.89256800
H	5.15839400	3.27892500	-1.70046000
H	4.79421800	5.70722000	-1.92158700
H	2.58986900	6.69689100	-1.42769200
H	0.71004500	5.27348400	-0.70484400
S	4.49188300	0.34997100	-1.09227800
I	0.46828400	2.04808300	-0.19479200
I	1.03242500	-2.13876300	0.23183500
C	-6.08607500	-1.38875000	-3.65356800

H	-6.52282200	-0.51952300	-4.14218700
H	-5.46053900	-1.93720200	-4.35138700
H	-6.87837800	-2.03085700	-3.27222900
H	-6.57880400	1.85372400	1.66325300

Transition state of the Michael addition reaction involving catalyst **6**

E (hartree) = -2865.38545

G (hartree) = -2864.761126

C	-4.09677500	-2.03149100	-0.35945100
H	-3.18808300	-2.24585700	-0.91301400
C	-3.89460500	-1.53544400	0.95537700
H	-4.68088500	-1.67347300	1.68396000
O	-1.82461800	-0.58519600	0.43212600
C	-5.23146200	-2.99514900	-0.56715200
C	-6.75528400	-0.12840300	-1.65341000
C	-5.65716500	0.20692200	-0.85442500
C	-5.86275900	0.94504000	0.30623300
C	-7.15862700	1.31759400	0.62705700
C	-8.24174700	0.96244800	-0.18422500
C	-8.05903100	0.22867200	-1.34437700
C	-4.95759000	-0.97056700	-2.65958700
C	-4.48296700	-0.41468100	-1.45056100
H	-5.02946600	1.23165300	0.93519500
H	-9.23954000	1.26990900	0.09720300
H	-8.89552000	-0.04630400	-1.97309100
H	-3.47261600	-0.04281200	-1.33917400
H	-4.40280300	-1.49962800	-3.42113000
N	-6.27670400	-0.85702500	-2.75147500
H	-6.19039300	-2.52592200	-0.33533500
H	-5.26612100	-3.39186400	-1.58029000
H	-5.10880400	-3.83287800	0.12185700
C	-2.76227000	-0.82180600	1.29077900
C	-2.59340500	-0.28162700	2.66905900
C	-1.30815500	-0.09874400	3.18142800
C	-3.68718200	0.05377200	3.46866700
C	-1.12106300	0.39142800	4.46406700
H	-0.45299700	-0.36422400	2.57215900
C	-3.49964800	0.55628700	4.74680600
H	-4.69300300	-0.06239500	3.08581300
C	-2.21655900	0.72338700	5.24988100

H	-0.11981600	0.50921200	4.85838400
H	-4.35693100	0.81842500	5.35237700
H	-2.07222000	1.10878200	6.25045400
C	-7.12933400	-1.42343300	-3.78100700
H	-7.70785300	-0.63070800	-4.25327300
H	-6.50909300	-1.91181100	-4.52761700
H	-7.80912900	-2.15137400	-3.33847400
H	-7.34150600	1.89981400	1.52035500
H	3.65158700	-1.57756500	2.76912600
C	3.45400400	-0.68245100	2.19335600
C	2.84528500	1.54526900	0.68577500
C	3.29697700	-0.79306300	0.81835400
C	3.33422300	0.54964200	2.81144400
C	2.99840900	1.66257300	2.05661600
C	3.04803900	0.32910800	0.02856200
H	3.46161900	0.63593600	3.88162700
H	2.83565300	2.62555700	2.52342800
N	2.40465200	2.70281000	-0.03484200
N	3.33250100	-2.11105600	0.26464400
C	2.24738900	-2.82820400	-0.09386000
N	2.65055400	-4.00977300	-0.55443700
C	4.48044600	-2.86396500	0.02556700
C	4.04193100	-4.07471100	-0.49818900
C	1.11011500	2.99361200	-0.29893200
N	1.05154500	4.19327400	-0.87000800
C	3.20468100	3.77419400	-0.43472800
C	2.33923300	4.71847400	-0.97391400
C	1.81544100	-5.10083900	-1.03817200
H	1.99317000	-5.98387200	-0.42684800
H	0.77238300	-4.80973000	-0.96636400
H	2.06969200	-5.31125300	-2.07554800
C	-0.14049200	4.89368300	-1.32894400
H	-0.06355900	5.06449600	-2.40132700
H	-1.01464600	4.28729600	-1.11416500
H	-0.21642700	5.84582000	-0.80679800
C	4.92917900	-5.08036100	-0.85975400
C	5.82696300	-2.58443000	0.21126900
C	4.57525200	3.97911900	-0.37408600
C	2.79875700	5.92492300	-1.48638200
H	4.59260800	-6.02415000	-1.26620300

H	6.16415300	-1.63891800	0.61371700
H	2.12915000	6.66123500	-1.90904900
H	5.24479700	3.23796900	0.04046400
C	6.27114000	-4.80850300	-0.67284900
C	6.71165400	-3.58366800	-0.14843300
C	5.03474100	5.18058600	-0.87976700
C	4.16327300	6.13503100	-1.42625000
H	7.00308200	-5.55856600	-0.93867100
H	7.77296000	-3.41877100	-0.02371900
H	4.57200600	7.05915000	-1.81082500
H	6.09491700	5.39173500	-0.85673000
C	3.02949500	0.30653200	-1.49208600
I	0.31218900	-2.07335800	0.09400300
I	-0.42865700	1.66042400	0.13319400
F	3.84390100	1.25447900	-1.96000400
F	1.81433300	0.54662300	-1.98672200
F	3.44204400	-0.85288100	-1.99578900

Starting material complex of the Michael addition reaction involving catalyst **6**

E (hartree) = -2865.419821

G (hartree) = -2864.796319

C	2.83500100	-3.63199800	2.25790100
H	1.90035500	-3.20969500	2.61495300
C	2.97760100	-3.80222100	0.94021300
H	3.88913600	-4.22404500	0.53944500
O	0.80466900	-3.06311700	0.40971200
C	3.86578300	-3.95395600	3.27417300
C	4.10342700	1.01820500	2.45302800
C	3.58338800	0.00020200	1.61830800
C	4.40275800	-0.52211200	0.60835200
C	5.67423200	-0.01224200	0.44077000
C	6.16192200	1.01287500	1.27130000
C	5.39007400	1.53564400	2.29015900
C	2.03336400	0.57354600	3.13411100
C	2.25006300	-0.26052600	2.07349500
H	4.04211600	-1.31912000	-0.03042300
H	7.16770700	1.38222600	1.12241200
H	5.77652700	2.30701500	2.94422600
H	1.54806700	-0.97583100	1.67315000
H	1.16211700	0.68114000	3.76080200

N	3.13896900	1.34769900	3.36974000
H	4.75742900	-4.39244300	2.83128000
H	4.14479500	-3.04228400	3.80931800
H	3.46036800	-4.64081200	4.02034000
C	1.91717500	-3.40174000	0.01229600
C	2.20839500	-3.36608000	-1.45264900
C	1.13288800	-3.33947400	-2.34663600
C	3.51014600	-3.30947200	-1.95365200
C	1.35546000	-3.25328300	-3.71123400
H	0.12537400	-3.41297800	-1.95722500
C	3.73028800	-3.20683300	-3.31979800
H	4.35941700	-3.32956600	-1.28420500
C	2.65617800	-3.18002300	-4.19867300
H	0.51934000	-3.25820200	-4.39797700
H	4.74221700	-3.15821800	-3.69878300
H	2.83162900	-3.11603900	-5.26447700
C	3.30153200	2.31769800	4.43102700
H	3.51800700	3.30463700	4.02004500
H	2.38005700	2.37091100	5.00510000
H	4.11442800	2.02532300	5.09629700
H	6.31801500	-0.41304400	-0.33146000
H	-4.39232500	-0.05499900	-2.63163800
C	-3.52190500	0.51904600	-2.34029400
C	-1.26932300	1.89491600	-1.55092700
C	-3.16759400	0.56065500	-0.99704600
C	-2.76601700	1.19003500	-3.28407800
C	-1.61967000	1.86219800	-2.88889000
C	-2.06038200	1.29123800	-0.56885300
H	-3.04804600	1.16115400	-4.32740400
H	-0.97985600	2.34966500	-3.61323100
N	-0.02549300	2.51301500	-1.19973700
N	-3.97947700	-0.19408400	-0.09584300
C	-3.62171100	-1.32607400	0.54355400
N	-4.65104700	-1.75387200	1.27042600
C	-5.29844700	0.11866800	0.23556100
C	-5.72231200	-0.87794400	1.10627100
C	1.16551900	1.87459600	-1.19250800
N	2.12836400	2.74026900	-0.90365700
C	0.19075000	3.86408200	-0.92253400
C	1.55976500	3.99972700	-0.72321400

C	-4.71684400	-2.94028400	2.11622500
H	-5.50546500	-3.59423400	1.74835000
H	-3.76530900	-3.46046800	2.08448200
H	-4.93504500	-2.63412500	3.13776500
C	3.56388200	2.48688900	-0.80169600
H	3.88210100	2.60781200	0.23319900
H	3.77812500	1.47038800	-1.11358200
H	4.08252800	3.19407000	-1.44616900
C	-7.00236900	-0.88515200	1.64612700
C	-6.12445700	1.16896000	-0.14115400
C	-0.67761900	4.94327100	-0.83788900
C	2.13824400	5.22611500	-0.41835700
H	-7.33607000	-1.65972600	2.32276400
H	-5.79084300	1.94689800	-0.81419600
H	3.20234800	5.33254400	-0.25916600
H	-1.74229100	4.83228600	-0.99055900
C	-7.82935600	0.15613900	1.27135800
C	-7.39805100	1.16453300	0.39556400
C	-0.10388900	6.16415700	-0.54101100
C	1.27791400	6.30289500	-0.33265700
H	-8.83613200	0.19735600	1.66343400
H	-8.08271600	1.95978700	0.13488400
H	1.67735200	7.28063700	-0.10135500
H	-0.73510400	7.03878700	-0.46527000
C	-1.68898300	1.51754100	0.88762700
I	-1.74073800	-2.17827200	0.42288500
I	1.38020700	-0.12475500	-1.62623800
F	-1.63486500	2.82781900	1.13460800
F	-0.49159300	1.00724400	1.17537700
F	-2.56462000	0.99614400	1.74294500

Product complex of the Michael addition reaction involving catalyst **6**

E (hartree) = -2865.394436

G (hartree) = -2864.767438

C	-4.14114400	-1.92368000	-0.08841000
H	-3.26419000	-2.41139800	-0.52788500
C	-3.78969600	-1.36467500	1.25057200
H	-4.45220600	-1.58156100	2.07759500
O	-1.83748300	-0.34796300	0.46145600
C	-5.29622400	-2.91316600	-0.00707300

C	-6.59280300	-0.03924600	-1.76235300
C	-5.66315700	0.09314300	-0.73686200
C	-5.98430600	0.87951000	0.35615500
C	-7.23441000	1.49030000	0.38154800
C	-8.14849800	1.33046700	-0.65827400
C	-7.83949200	0.55425000	-1.76729100
C	-4.82495300	-1.23563100	-2.41562200
C	-4.45745500	-0.72547600	-1.08244000
H	-5.27902300	1.01562200	1.16430100
H	-9.11115100	1.81982400	-0.60438100
H	-8.54141700	0.42930700	-2.58087700
H	-3.51929800	-0.15600100	-1.11245800
H	-4.23746700	-1.88627700	-3.05082500
N	-6.01573800	-0.87379900	-2.76486600
H	-6.19937700	-2.42761400	0.36787400
H	-5.52483600	-3.36999100	-0.97252100
H	-5.04259600	-3.71507400	0.68552500
C	-2.69319500	-0.60161800	1.44451700
C	-2.39403300	-0.01417200	2.78254300
C	-1.07183500	0.15503600	3.19433200
C	-3.41304500	0.37374200	3.65308800
C	-0.77819200	0.68416200	4.44213900
H	-0.26706200	-0.14885900	2.53669400
C	-3.12118300	0.90886800	4.89830500
H	-4.44691000	0.26245500	3.35124800
C	-1.80137700	1.06561900	5.29863000
H	0.25304200	0.79288700	4.75399300
H	-3.92570600	1.20763100	5.55724200
H	-1.57366700	1.48088300	6.27126800
C	-6.72364000	-1.24708100	-3.98280900
H	-6.98267100	-0.34317000	-4.53133000
H	-6.08352400	-1.88151900	-4.58860600
H	-7.63253500	-1.78164000	-3.71121400
H	-7.50546200	2.10667400	1.22819200
H	3.45287700	-1.73238300	2.73958200
C	3.32853900	-0.82982600	2.15508800
C	2.89624700	1.42418700	0.62657700
C	3.16076700	-0.94195000	0.78147000
C	3.30753700	0.41360800	2.76137300
C	3.05907100	1.54221700	1.99623400

C	3.00151000	0.18940000	-0.01839000
H	3.44179200	0.49950700	3.83073800
H	2.97127600	2.51915600	2.45400400
N	2.54419000	2.60636700	-0.10354100
N	3.08148700	-2.26458800	0.24403700
C	1.93224900	-2.89248600	-0.08542400
N	2.23337200	-4.11415100	-0.52109500
C	4.15962400	-3.11634700	0.01344500
C	3.61463000	-4.29735900	-0.47730200
C	1.27365300	2.99760400	-0.35715600
N	1.30806300	4.19767600	-0.93071400
C	3.42387200	3.60914500	-0.51291100
C	2.63226900	4.61883300	-1.04714900
C	1.30019900	-5.13813100	-0.96665600
H	1.40977000	-6.02063500	-0.33862400
H	0.28857400	-4.75348400	-0.88361100
H	1.51563300	-5.39349600	-2.00286000
C	0.17080000	4.98897300	-1.37775900
H	0.24347000	5.14604500	-2.45262300
H	-0.74542600	4.45626800	-1.14335300
H	0.17989900	5.94800400	-0.86274700
C	4.41021500	-5.38218500	-0.82178600
C	5.52661700	-2.94872200	0.18179700
C	4.80678800	3.70403400	-0.46446600
C	3.18268200	5.78374700	-1.56565100
H	3.99084900	-6.30328500	-1.20255400
H	5.94644900	-2.02631400	0.55948300
H	2.57048700	6.57086200	-1.98386400
H	5.41796300	2.91177100	-0.05426700
C	5.77276300	-5.22163700	-0.65242400
C	6.32056000	-4.02743000	-0.16088900
C	5.35706900	4.86407500	-0.97659400
C	4.56029900	5.88440200	-1.51743100
H	6.43550600	-6.03742300	-0.90581800
H	7.39312200	-3.95030400	-0.04800500
H	5.03823600	6.77243600	-1.90718700
H	6.43094800	4.98937500	-0.96309100
C	2.97746600	0.15396900	-1.53807500
I	0.06066400	-1.93382600	0.11609300
I	-0.35808800	1.77384500	0.09875000

F	3.86804400	1.02685200	-2.01596400
F	1.78675700	0.49103800	-2.03579400
F	3.29159900	-1.03976900	-2.03199800

Transition state of the nitro-Michael addition reaction involving catalyst **2**

E (hartree) = -2599.748415

G (hartree) = -2599.320700

C	3.27270200	-0.10022600	-1.65517500
H	3.02905200	-0.60761700	-2.58047200
C	2.18340600	0.17871100	-0.81078000
H	2.19291400	0.89573900	-0.00859600
N	1.05188400	-0.52979100	-0.93683700
O	0.93682700	-1.48564100	-1.70853900
O	0.03500600	-0.21247600	-0.19415500
C	4.45585300	0.74487100	-1.65203400
C	5.20648200	0.85987900	-2.82853400
C	4.86677700	1.44206000	-0.51010100
C	6.32049900	1.67826200	-2.87391600
H	4.89731600	0.31469100	-3.71337600
C	5.98974700	2.25017800	-0.55455400
H	4.32796600	1.32695700	0.42359100
C	6.71262900	2.37478800	-1.73524200
H	6.88314500	1.77697800	-3.79237500
H	6.30544500	2.78202700	0.33291700
H	7.58524600	3.01386400	-1.76866100
C	5.75935700	-1.43100100	0.44298100
C	4.37062000	-1.61739400	0.51564900
C	3.69320200	-1.29397000	1.68333300
C	4.42296400	-0.79920900	2.76147000
C	5.81716200	-0.62884900	2.67009600
C	6.49796200	-0.94583300	1.50758000
C	5.10462900	-2.21404500	-1.54780200
C	3.93790600	-2.06397400	-0.78933100
H	2.62616100	-1.43842600	1.79501700
H	6.37577700	-0.25646500	3.51543100
H	7.56956500	-0.80987500	1.44462000
H	7.10380200	-1.76048200	-1.18971400
H	2.99328200	-2.52109900	-1.04175000
H	5.21503700	-2.55776500	-2.56464700
N	6.15768200	-1.80227500	-0.84212200

O	3.70900000	-0.50859900	3.86726900
C	4.40138000	-0.09381700	5.03321200
H	4.91874300	0.85366700	4.86713400
H	3.64437700	0.03725900	5.80015600
H	5.11629100	-0.85407900	5.35388800
C	-4.65776300	-2.25620600	0.06873500
C	-3.62237000	-3.16153600	-0.17833600
C	-3.81270000	-4.52484400	-0.27051700
C	-5.10775100	-5.00021400	-0.10634900
C	-6.16201900	-4.12503200	0.14053800
C	-5.94741700	-2.76083500	0.22870700
C	-4.25398700	-0.86691000	0.12683400
H	-2.99685400	-5.20850300	-0.46289000
H	-5.29249400	-6.06380800	-0.17248000
H	-7.16242000	-4.51615100	0.26525700
H	-6.77420900	-2.08805800	0.42118400
C	-2.93691700	-0.52453800	-0.03414500
C	-2.60357900	3.60085400	0.11113700
C	-3.86234900	3.01975000	0.28225100
C	-4.95127500	3.86788500	0.47787900
C	-4.75594800	5.23793500	0.49644100
C	-3.48667800	5.78236700	0.32237000
C	-2.38237100	4.96195600	0.12702700
C	-2.72982200	0.86751100	0.02369900
C	-3.88683900	1.57277500	0.22441500
H	-5.94314800	3.45449100	0.61392000
H	-5.60187200	5.89432300	0.64798000
H	-3.35151800	6.85530300	0.33808400
H	-1.39757900	5.38839500	-0.00768200
S	-5.23962900	0.52214900	0.35393300
I	-1.11826300	2.13904200	-0.13567900
I	-1.77729400	-2.18575200	-0.38561400

Energy profile of the nitro-Michael addition reaction

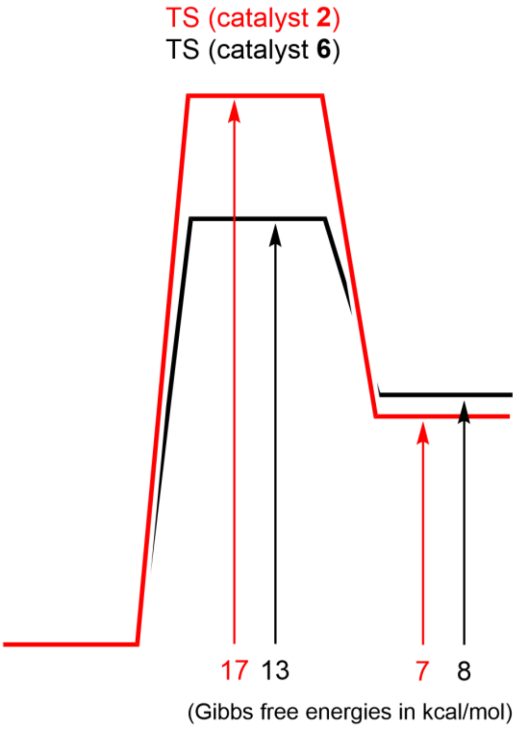

Starting material complex of the nitro-Michael addition reaction involving catalyst **2**

E (hartree) = -2599.775017

G (hartree) = -2599.348144

C	3.48188100	-2.35527300	-0.55940100
H	3.33324500	-3.29781900	-0.04226300
C	2.49039400	-1.45376000	-0.47849200
H	2.46811100	-0.45299900	-0.87908200
N	1.30287600	-1.79942700	0.21801400
O	1.15970700	-2.87134400	0.75287500
O	0.38967400	-0.94596200	0.23140000
C	4.73273000	-2.17922400	-1.25834800
C	5.74105100	-3.12856400	-1.05245800
C	4.98136400	-1.08356500	-2.09702300
C	6.98381700	-2.97066400	-1.64180400
H	5.54454000	-3.98463300	-0.41804800
C	6.21916100	-0.93361900	-2.69077700
H	4.20261900	-0.35739000	-2.29271900
C	7.22403800	-1.87129500	-2.45704200
H	7.76034700	-3.70494700	-1.47625400
H	6.40727400	-0.09508900	-3.34807800
H	8.19126300	-1.75029300	-2.92710200

C	4.70052800	0.66952700	0.86939000
C	3.66999600	0.52848000	1.83207400
C	2.61062900	1.44452900	1.82390700
C	2.61854600	2.44356400	0.87188500
C	3.62984800	2.56103900	-0.09179300
C	4.68685500	1.67320100	-0.09851000
C	5.18901200	-1.08334100	2.16463400
C	4.00454800	-0.60041500	2.64111400
H	1.82352800	1.39479700	2.56811200
H	3.57888900	3.37233000	-0.80547100
H	5.48780000	1.76967500	-0.82012500
H	6.44752300	-0.48643800	0.56972200
H	3.45918700	-0.98659000	3.48547600
H	5.78595900	-1.90960200	2.51493500
N	5.61226000	-0.32070300	1.10758800
O	1.57305800	3.36906500	0.83217000
C	1.58490300	4.30420300	1.92189100
H	2.48926000	4.91035300	1.86843700
H	0.70524500	4.93524800	1.81541400
H	1.54772100	3.78130500	2.87734400
C	-4.78541200	-1.87251600	-0.22652000
C	-4.00121900	-2.95505000	0.17818300
C	-4.48344900	-4.23474700	0.35121900
C	-5.83593300	-4.43670900	0.10384600
C	-6.65094300	-3.38359000	-0.30158500
C	-6.13824700	-2.10869100	-0.46757700
C	-4.08840400	-0.60892700	-0.33883000
H	-3.85182000	-5.05416900	0.66610100
H	-6.25279800	-5.42663300	0.23030300
H	-7.70061300	-3.56355400	-0.48925200
H	-6.78187600	-1.29664200	-0.78289600
C	-2.74593600	-0.52583900	-0.07283700
C	-1.54238000	3.41798600	-0.42899300
C	-2.86640900	3.07929700	-0.71743000
C	-3.71907800	4.10142400	-1.13296700
C	-3.23365300	5.39169200	-1.25598500
C	-1.89987300	5.68325600	-0.98431500
C	-1.02459400	4.68755900	-0.56706900
C	-2.22512600	0.77797000	-0.20869000
C	-3.19650400	1.67676300	-0.57019400

H	-4.75401200	3.88197000	-1.36541400
H	-3.89772100	6.18156100	-1.57929600
H	-1.53243600	6.69354300	-1.10308700
H	0.01896100	4.89719400	-0.37921600
S	-4.72977700	0.92596800	-0.76138900
I	-0.42737300	1.74307200	0.14694800
I	-2.00398600	-2.38020400	0.45922000

Product complex of the nitro-Michael addition reaction involving catalyst **2**

E (hartree) = -2599.767196

G (hartree) = -2599.336790

C	3.14875700	0.51963800	-1.41705100
H	2.70096500	0.62534800	-2.40978500
C	2.04363100	0.54081100	-0.41839000
H	2.06640700	1.12715700	0.48575500
N	0.99454800	-0.20098900	-0.62106600
O	0.87380200	-0.98551100	-1.60023100
O	-0.01884500	-0.15246400	0.23580100
C	4.15719900	1.62719200	-1.20363000
C	4.53827700	2.42118100	-2.28282000
C	4.74949900	1.84646700	0.03926700
C	5.49797100	3.41256100	-2.12838800
H	4.06940800	2.27502900	-3.25007200
C	5.70840600	2.83829900	0.19397300
H	4.46422700	1.24338200	0.89385500
C	6.08651900	3.62046300	-0.88913900
H	5.77786600	4.02719600	-2.97361300
H	6.15827000	3.00308900	1.16414000
H	6.83124000	4.39521200	-0.76521800
C	5.86605300	-1.45683000	-0.38422200
C	4.49432000	-1.33928300	-0.16403800
C	3.98378900	-1.61014500	1.08195400
C	4.87506000	-1.98313700	2.10194100
C	6.25266800	-2.08117400	1.85530900
C	6.76589000	-1.82063500	0.59348200
C	5.00518800	-0.85493400	-2.37062500
C	3.84523500	-0.89854900	-1.44501000
H	2.92752800	-1.55147400	1.30901200
H	6.92946500	-2.36980800	2.64491400
H	7.82705500	-1.90637700	0.40073100

H	7.01428600	-1.14806800	-2.18077700
H	3.08047900	-1.59801500	-1.80503000
H	5.00404600	-0.58094300	-3.41681200
N	6.09582200	-1.14875300	-1.74862400
O	4.31267300	-2.22873800	3.28802900
C	5.13874600	-2.63131400	4.37400000
H	5.86585300	-1.85413700	4.61568400
H	4.47016600	-2.77956100	5.21550300
H	5.65109700	-3.56678300	4.14312800
C	-4.57514800	-2.37874400	-0.27312600
C	-3.47771600	-3.21945000	-0.48373300
C	-3.60122400	-4.57230600	-0.72724100
C	-4.88397700	-5.10430400	-0.75873700
C	-5.99623000	-4.29278600	-0.55249300
C	-5.85147500	-2.93804500	-0.31114100
C	-4.24075100	-0.99002500	-0.03578900
H	-2.74036100	-5.20693800	-0.88935000
H	-5.01392600	-6.16151000	-0.94607300
H	-6.98643200	-4.72639600	-0.58104700
H	-6.72221300	-2.31393500	-0.15154600
C	-2.92858800	-0.60069900	-0.00845700
C	-2.71549400	3.47472200	0.55600300
C	-3.97767500	2.87417500	0.53131800
C	-5.09984400	3.68751600	0.67792000
C	-4.93663600	5.05206800	0.84226800
C	-3.66688500	5.62201200	0.86353900
C	-2.53252500	4.83241400	0.72181800
C	-2.77338600	0.77815200	0.21110400
C	-3.96494300	1.43761800	0.33935500
H	-6.09169300	3.25245900	0.66288100
H	-5.80739600	5.68314700	0.95567100
H	-3.55635400	6.69019200	0.99186700
H	-1.54784100	5.28024000	0.74082400
S	-5.29094000	0.35058300	0.20762600
I	-1.17354200	2.05457400	0.36230500
I	-1.66228800	-2.16671600	-0.39538000

Transition state of the nitro-Michael addition reaction involving catalyst **6**

E (hartree) = -2992.491429

G (hartree) = -2991.896986

C	-4.39939000	-0.16442600	-1.51076900
H	-3.91270000	0.32457100	-2.34654700
C	-3.51613700	-0.65546700	-0.51757200
H	-3.74298500	-1.45882800	0.16085900
N	-2.33042600	-0.07093900	-0.32290900
O	-1.99296400	0.94131800	-1.00273800
O	-1.54872900	-0.50775300	0.56969000
C	-5.59780700	-0.95147000	-1.84566000
C	-6.04704400	-0.97345200	-3.16816700
C	-6.30487300	-1.66648400	-0.87727200
C	-7.16514700	-1.70991500	-3.52140700
H	-5.50261300	-0.42055000	-3.92589000
C	-7.42996300	-2.39611700	-1.23034700
H	-5.98869600	-1.63892200	0.15918700
C	-7.85977200	-2.42212000	-2.55020800
H	-7.49357300	-1.73373100	-4.55185700
H	-7.97153500	-2.94673200	-0.47269900
H	-8.73364500	-2.99839400	-2.82394000
C	-7.11599500	1.20965700	0.18391300
C	-5.74891800	1.21633600	0.48958800
C	-5.31884000	0.79054100	1.73503600
C	-6.27639000	0.37168500	2.65880900
C	-7.64435900	0.37303200	2.33147600
C	-8.07744400	0.79443700	1.08547000
C	-6.05551000	1.94662000	-1.64322800
C	-5.04209800	1.61147500	-0.71926200
H	-4.27446500	0.78395500	2.01954900
H	-8.37710500	0.05035800	3.05541100
H	-9.13069100	0.78867100	0.83809000
H	-8.11588600	1.71900500	-1.64201300
H	-4.05206300	2.04503100	-0.75282600
H	-5.95494700	2.30918000	-2.65480200
N	-7.24479900	1.65967300	-1.13547300
O	-5.79956400	-0.02118700	3.85457700
C	-6.71871700	-0.41751200	4.85742500
H	-7.28794700	-1.29528400	4.54347000
H	-6.12118300	-0.66671600	5.72876800
H	-7.40229300	0.39711300	5.10547600
H	3.49794900	2.06920700	-3.06126700
C	3.54802000	1.13571500	-2.51579800

C	3.58830900	-1.21940100	-1.06370800
C	3.51478300	1.16260900	-1.13261300
C	3.62583400	-0.07841600	-3.17960800
C	3.62170800	-1.25446000	-2.45148400
C	3.58264100	-0.00816700	-0.37328400
H	3.65423900	-0.10749400	-4.25992000
H	3.62922500	-2.21651400	-2.94759700
N	3.52355700	-2.47469400	-0.38566700
N	3.36786100	2.43352100	-0.49104100
C	2.18599700	2.98859400	-0.14350200
N	2.42000000	4.19542500	0.36795200
C	4.39849000	3.33312300	-0.21376800
C	3.78985200	4.45181100	0.34098200
C	2.43701500	-2.97435800	0.23615700
N	2.75322100	-4.15382600	0.76646200
C	4.58375900	-3.36941000	-0.24818500
C	4.08825600	-4.43976600	0.48714300
C	1.44257400	5.14472600	0.88392800
H	1.49123200	6.06076000	0.29766700
H	0.44987400	4.71316200	0.81039100
H	1.67339600	5.35924400	1.92590000
C	1.88707700	-5.04756800	1.52467800
H	2.31160700	-5.19521000	2.51614500
H	0.90076000	-4.60364900	1.61101100
H	1.81761200	-6.00166700	1.00517300
C	4.52393300	5.55832900	0.74888000
C	5.77146500	3.25350900	-0.39461000
C	5.89920700	-3.32244900	-0.68809200
C	4.88169900	-5.53048700	0.81848300
H	4.05426600	6.43034900	1.18266500
H	6.24063500	2.37699000	-0.82010500
H	4.49992100	-6.36537800	1.38977600
H	6.28270500	-2.48510600	-1.25498000
C	5.89198000	5.48554300	0.56833500
C	6.50399800	4.35472400	0.00631100
C	6.69096600	-4.40650900	-0.35851100
C	6.19149300	-5.49032500	0.37951800
H	6.50876900	6.32141400	0.86801500
H	7.57857600	4.34568700	-0.11329600
H	6.85097000	-6.31505000	0.61164600

H	7.72423000	-4.42119600	-0.67667700
C	3.69668500	0.11761700	1.13878900
I	0.34177400	2.06493900	-0.43369100
I	0.61864000	-1.97393000	0.33835200
F	3.89053600	-1.04822300	1.74864700
F	2.61034300	0.67486600	1.67496300
F	4.73629200	0.89761000	1.44232700

Starting material complex of the nitro-Michael addition reaction involving catalyst **6**

E (hartree) = -2992.510834

G (hartree) = -2991.918185

C	4.41485700	2.23092800	-1.17121900
H	4.24554200	2.00752500	-2.21879000
C	3.34175300	2.43622900	-0.39730400
H	3.32484500	2.72917400	0.63860600
N	2.03506100	2.23820900	-0.92312800
O	1.87213500	1.78349300	-2.04801300
O	1.09631300	2.50645300	-0.18073400
C	5.78812800	2.29059900	-0.71192600
C	6.81060100	2.40132700	-1.66052500
C	6.11688900	2.21056800	0.64598600
C	8.13309000	2.46840000	-1.25998700
H	6.55700400	2.44880900	-2.71287000
C	7.44032300	2.26827200	1.04246900
H	5.33992600	2.06285900	1.38756400
C	8.44765100	2.40522700	0.09273100
H	8.91823800	2.57179400	-1.99663500
H	7.69090200	2.20273300	2.09295500
H	9.48153400	2.45902700	0.40815700
C	5.63448700	-0.97598000	0.76829400
C	4.33536800	-0.74701900	0.26331000
C	3.30476700	-0.44938500	1.16234700
C	3.57928400	-0.40280400	2.51937800
C	4.87987400	-0.64986200	3.00174100
C	5.91187500	-0.93707300	2.12841500
C	5.73899200	-1.10575400	-1.45957400
C	4.42865200	-0.84383800	-1.16504800
H	2.29281900	-0.23690600	0.83908700
H	5.08582000	-0.61764200	4.06100900
H	6.91113100	-1.11535600	2.50447900

H	7.45561100	-1.35254600	-0.25911100
H	3.64707800	-0.70183800	-1.89331100
H	6.21478600	-1.23688800	-2.41779400
N	6.46541900	-1.18501400	-0.30913500
O	2.52487800	-0.11136100	3.32738200
C	2.75642500	-0.01685000	4.71894800
H	3.48337200	0.76638100	4.94685700
H	1.80094700	0.23827000	5.16808700
H	3.10593600	-0.96754600	5.12855900
H	-2.56678100	-2.87703300	-3.07897300
C	-2.98277600	-2.07502000	-2.48282900
C	-3.94101600	0.01211900	-0.93394100
C	-2.54829500	-1.90763800	-1.18062600
C	-3.92177300	-1.20242300	-3.01146800
C	-4.37777000	-0.14642700	-2.24413400
C	-3.05129300	-0.89379900	-0.35842200
H	-4.26740900	-1.32475900	-4.02854000
H	-5.07185200	0.57710400	-2.65250400
N	-4.43957900	1.14965200	-0.22731000
N	-1.49948600	-2.76047200	-0.70670900
C	-0.18652900	-2.46659800	-0.80525100
N	0.52861000	-3.46094500	-0.29317000
C	-1.63351900	-4.02218300	-0.12444100
C	-0.34127000	-4.45842300	0.14594000
C	-3.73916000	2.24749800	0.12080400
N	-4.55049700	3.10122400	0.73958000
C	-5.76307500	1.30639600	0.18664100
C	-5.82906600	2.55048800	0.80212000
C	1.98142500	-3.54009900	-0.15634400
H	2.31571900	-4.49049800	-0.56719900
H	2.45035900	-2.72259900	-0.69690300
H	2.24536900	-3.46834200	0.89772600
C	-4.22295500	4.41440100	1.28376200
H	-4.42968600	4.41482100	2.35247100
H	-3.17247100	4.62532200	1.11351400
H	-4.83383800	5.16561400	0.78647000
C	-0.09370400	-5.68816500	0.74407400
C	-2.75031500	-4.78757200	0.17946600
C	-6.87085000	0.47643800	0.08027700
C	-7.01155800	3.04016800	1.34238400

H	0.90921200	-6.02795200	0.96208500
H	-3.75324600	-4.44043900	-0.02762700
H	-7.06681800	4.00806100	1.82119800
H	-6.81617400	-0.49392000	-0.39391100
C	-1.20205600	-6.45418000	1.04682400
C	-2.50569900	-6.01287100	0.76807300
C	-8.04812000	0.96102400	0.61791600
C	-8.11760700	2.21925700	1.23596600
H	-1.06417600	-7.42062400	1.51152800
H	-3.34031500	-6.65075000	1.02429800
H	-9.06302000	2.55331200	1.64043500
H	-8.94178200	0.35487800	0.56228900
C	-2.64287300	-0.88755900	1.10783000
I	0.53620000	-0.72606600	-1.63083200
I	-1.71654300	2.47757400	-0.20982600
F	-3.27478800	0.03758700	1.82336700
F	-1.33198600	-0.68735800	1.25828600
F	-2.93019000	-2.07068800	1.65232100

Product complex of the nitro-Michael addition reaction involving catalyst **6**

E (hartree) = -2992.502520

G (hartree) = -2991.905795

C	4.21659700	0.37404700	-1.45288200
H	3.62253000	0.21183200	-2.35618400
C	3.30553200	0.74233000	-0.33819100
H	3.48459900	1.57058100	0.32706100
N	2.25454300	0.01870200	-0.08628800
O	1.97244600	-1.00819100	-0.81336900
O	1.48468600	0.28250500	0.91721900
C	5.27116200	1.42369300	-1.72567400
C	5.49815700	1.84533200	-3.03368400
C	6.06210000	1.94489800	-0.70274700
C	6.49917400	2.76383600	-3.31940100
H	4.87506300	1.46401500	-3.83560900
C	7.06348900	2.86299600	-0.98790700
H	5.89571800	1.63831900	0.32362000
C	7.28650600	3.27177000	-2.29586700
H	6.65666200	3.08931400	-4.33917600
H	7.66688000	3.26490600	-0.18461700
H	8.06410400	3.99123100	-2.51483700

C	7.00848700	-1.34548800	-0.20333200
C	5.66927200	-1.12731900	0.11881700
C	5.29673800	-1.01838300	1.43704400
C	6.29668800	-1.11503400	2.41880700
C	7.63919000	-1.32022400	2.06654900
C	8.01105300	-1.44401500	0.73595000
C	5.92939400	-1.30166100	-2.17284100
C	4.88508900	-1.03790800	-1.15737400
H	4.27097400	-0.86543000	1.74728800
H	8.39880100	-1.39308500	2.82972600
H	9.04474600	-1.61205100	0.46446400
H	7.95019300	-1.58274700	-2.12550800
H	4.06196600	-1.75982300	-1.21414100
H	5.81351600	-1.32878600	-3.24743400
N	7.08535100	-1.44141000	-1.61481300
O	5.87046300	-0.99848800	3.68020400
C	6.81270500	-1.09755800	4.73842200
H	7.55992300	-0.30476100	4.66839400
H	6.24293600	-0.98095200	5.65448300
H	7.30115600	-2.07374500	4.73237300
H	-3.32661700	-1.80676600	-3.20483100
C	-3.36810900	-0.91139200	-2.59832800
C	-3.38610000	1.33793400	-0.98536500
C	-3.42194400	-1.03476200	-1.22129700
C	-3.34834300	0.34787200	-3.17656100
C	-3.33214300	1.46898600	-2.36677800
C	-3.48255200	0.08321800	-0.38557300
H	-3.30929600	0.45288400	-4.25178000
H	-3.26222300	2.46204800	-2.79166500
N	-3.30025700	2.53834200	-0.21778000
N	-3.36671500	-2.35171000	-0.66392100
C	-2.22810500	-2.97782100	-0.29118000
N	-2.54197300	-4.20380100	0.12496000
C	-4.44733400	-3.21985400	-0.50329800
C	-3.91710500	-4.39797300	0.00731000
C	-2.22811200	2.93040700	0.50122300
N	-2.51865500	4.09165400	1.08606000
C	-4.31811900	3.48190200	-0.08833200
C	-3.81471200	4.47330900	0.74560800
C	-1.63317400	-5.22289800	0.63058900

H	-1.68141000	-6.09799800	-0.01533000
H	-0.62287800	-4.82714700	0.63989100
H	-1.93068800	-5.49350100	1.64224000
C	-1.65666400	4.87927000	1.95644100
H	-2.12800700	4.97463900	2.93318000
H	-0.69973300	4.37798300	2.05873700
H	-1.51133000	5.86515900	1.51822500
C	-4.71789100	-5.49440600	0.30007200
C	-5.80343600	-3.06816700	-0.75144900
C	-5.60272800	3.53924700	-0.60991000
C	-4.56887700	5.58447700	1.09907400
H	-4.30927700	-6.41238700	0.69946800
H	-6.21134100	-2.14530500	-1.14055000
H	-4.18096200	6.35795400	1.74751100
H	-5.99304300	2.76225600	-1.25303400
C	-6.06979300	-5.34979500	0.05174000
C	-6.60232000	-4.15924200	-0.46505000
C	-6.35585700	4.64419400	-0.25825300
C	-5.84815900	5.64838100	0.57908700
H	-6.73598100	-6.17509100	0.26156500
H	-7.66719100	-4.09419200	-0.64073100
H	-6.47604800	6.49343500	0.82520900
H	-7.36419600	4.73783600	-0.63716600
C	-3.70905200	-0.14078400	1.10212100
I	-0.33072200	-2.10317500	-0.41760900
I	-0.46553300	1.79163200	0.66455400
F	-3.87743000	0.98823900	1.78392200
F	-2.70238600	-0.80570100	1.66826700
F	-4.81452500	-0.87125000	1.27399500

Energy profile of the Diels-Alder reaction

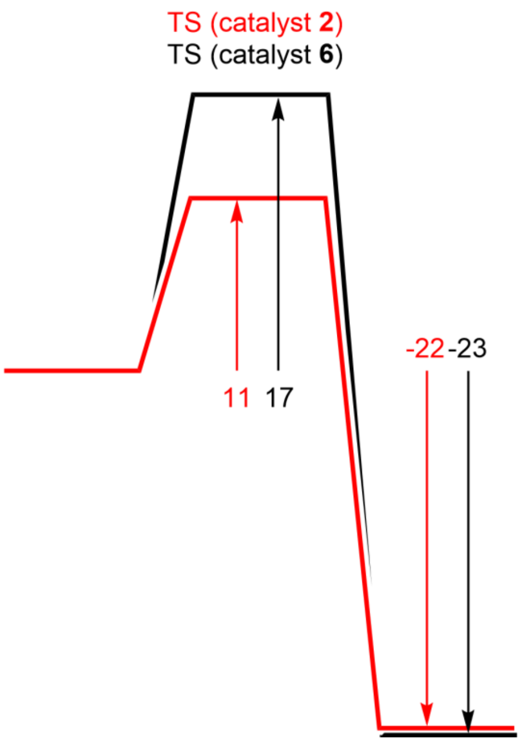

Transition state of the Diels-Alder reaction involving catalyst **2**

E (hartree) = -2071.876634

G (hartree) = -2071.527862

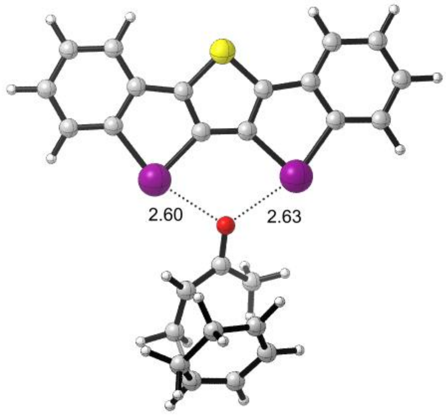

C	-4.39939000	-0.16442600	-1.51076900
H	-3.91270000	0.32457100	-2.34654700
C	-3.51613700	-0.65546700	-0.51757200
H	-3.74298500	-1.45882800	0.16085900
N	-2.33042600	-0.07093900	-0.32290900
O	-1.99296400	0.94131800	-1.00273800

O	-1.54872900	-0.50775300	0.56969000
C	-5.59780700	-0.95147000	-1.84566000
C	-6.04704400	-0.97345200	-3.16816700
C	-6.30487300	-1.66648400	-0.87727200
C	-7.16514700	-1.70991500	-3.52140700
H	-5.50261300	-0.42055000	-3.92589000
C	-7.42996300	-2.39611700	-1.23034700
H	-5.98869600	-1.63892200	0.15918700
C	-7.85977200	-2.42212000	-2.55020800
H	-7.49357300	-1.73373100	-4.55185700
H	-7.97153500	-2.94673200	-0.47269900
H	-8.73364500	-2.99839400	-2.82394000
C	-7.11599500	1.20965700	0.18391300
C	-5.74891800	1.21633600	0.48958800
C	-5.31884000	0.79054100	1.73503600
C	-6.27639000	0.37168500	2.65880900
C	-7.64435900	0.37303200	2.33147600
C	-8.07744400	0.79443700	1.08547000
C	-6.05551000	1.94662000	-1.64322800
C	-5.04209800	1.61147500	-0.71926200
H	-4.27446500	0.78395500	2.01954900
H	-8.37710500	0.05035800	3.05541100
H	-9.13069100	0.78867100	0.83809000
H	-8.11588600	1.71900500	-1.64201300
H	-4.05206300	2.04503100	-0.75282600
H	-5.95494700	2.30918000	-2.65480200
N	-7.24479900	1.65967300	-1.13547300
O	-5.79956400	-0.02118700	3.85457700
C	-6.71871700	-0.41751200	4.85742500
H	-7.28794700	-1.29528400	4.54347000
H	-6.12118300	-0.66671600	5.72876800
H	-7.40229300	0.39711300	5.10547600
H	3.49794900	2.06920700	-3.06126700
C	3.54802000	1.13571500	-2.51579800
C	3.58830900	-1.21940100	-1.06370800
C	3.51478300	1.16260900	-1.13261300
C	3.62583400	-0.07841600	-3.17960800
C	3.62170800	-1.25446000	-2.45148400
C	3.58264100	-0.00816700	-0.37328400
H	3.65423900	-0.10749400	-4.25992000

H	3.62922500	-2.21651400	-2.94759700
N	3.52355700	-2.47469400	-0.38566700
N	3.36786100	2.43352100	-0.49104100
C	2.18599700	2.98859400	-0.14350200
N	2.42000000	4.19542500	0.36795200
C	4.39849000	3.33312300	-0.21376800
C	3.78985200	4.45181100	0.34098200
C	2.43701500	-2.97435800	0.23615700
N	2.75322100	-4.15382600	0.76646200
C	4.58375900	-3.36941000	-0.24818500
C	4.08825600	-4.43976600	0.48714300
C	1.44257400	5.14472600	0.88392800
H	1.49123200	6.06076000	0.29766700
H	0.44987400	4.71316200	0.81039100
H	1.67339600	5.35924400	1.92590000
C	1.88707700	-5.04756800	1.52467800
H	2.31160700	-5.19521000	2.51614500
H	0.90076000	-4.60364900	1.61101100
H	1.81761200	-6.00166700	1.00517300
C	4.52393300	5.55832900	0.74888000
C	5.77146500	3.25350900	-0.39461000
C	5.89920700	-3.32244900	-0.68809200
C	4.88169900	-5.53048700	0.81848300
H	4.05426600	6.43034900	1.18266500
H	6.24063500	2.37699000	-0.82010500
H	4.49992100	-6.36537800	1.38977600
H	6.28270500	-2.48510600	-1.25498000
C	5.89198000	5.48554300	0.56833500
C	6.50399800	4.35472400	0.00631100
C	6.69096600	-4.40650900	-0.35851100
C	6.19149300	-5.49032500	0.37951800
H	6.50876900	6.32141400	0.86801500
H	7.57857600	4.34568700	-0.11329600
H	6.85097000	-6.31505000	0.61164600
H	7.72423000	-4.42119600	-0.67667700
C	3.69668500	0.11761700	1.13878900
I	0.34177400	2.06493900	-0.43369100
I	0.61864000	-1.97393000	0.33835200
F	3.89053600	-1.04822300	1.74864700
F	2.61034300	0.67486600	1.67496300

F	4.73629200	0.89761000	1.44232700

Starting material complex of the Diels-Alder reaction involving catalyst **2**

E (hartree) = -2071.890077

G (hartree) = -2071.545063

C	-3.86486000	0.32730600	1.48360700
C	-4.31521500	1.47510700	2.34645100
C	-5.81052200	1.75330000	2.19169800
C	-6.25994700	1.65819200	0.76119900
C	-5.65273900	0.83087700	-0.09390800
C	-4.50009000	0.04016500	0.34318600
H	-6.38983300	1.01996000	2.76575800
H	-4.07639100	1.27892000	3.39148300
H	-3.74508200	2.36973900	2.06661300
H	-3.01811500	-0.26533000	1.81434000
H	-7.15055000	2.19698100	0.45996700
H	-6.04781900	0.68519200	-1.09255900
H	-4.19731000	-0.80341200	-0.27113100
H	-6.05670400	2.72747600	2.61299500
C	-2.57958400	2.86551100	-0.40133700
H	-1.95146900	3.11230800	0.44736900
C	-3.69121200	3.55467200	-0.65976300
H	-3.98567200	4.39342500	-0.04173800
H	-4.34228100	3.31501400	-1.49039200
C	-2.15551000	1.71199700	-1.18387400
C	-2.94084500	1.27010000	-2.37675800
H	-3.99860800	1.17334400	-2.13676000
H	-2.85090500	2.02940400	-3.15826000
H	-2.55093600	0.33172500	-2.76326700
O	-1.10917600	1.11685600	-0.87446600
C	3.94883500	0.96656100	0.37051600
C	3.41554500	2.23668000	0.14007000
C	4.14669800	3.40341000	0.19506600
C	5.49731800	3.28388000	0.50132600
C	6.06726800	2.03624000	0.73873700
C	5.30657300	0.88149800	0.67584700
C	3.01082100	-0.13101800	0.26075200
H	3.70717000	4.37415700	0.01057400
H	6.10635600	4.17620500	0.55346700
H	7.12028000	1.96745400	0.97517300

H	5.76138900	-0.08392400	0.86138100
C	1.69749900	0.10282600	-0.05653200
C	-0.31442000	-3.52577500	-0.13647400
C	1.05078800	-3.51021900	0.15783400
C	1.66923000	-4.73179200	0.42108300
C	0.92444300	-5.89788900	0.38280100
C	-0.43517500	-5.87057300	0.08524000
C	-1.08169000	-4.66954100	-0.18242900
C	0.93370800	-1.08011500	-0.11859500
C	1.67144200	-2.20203500	0.15905300
H	2.72652500	-4.76558700	0.65409800
H	1.40643800	-6.84407900	0.58738200
H	-1.00252000	-6.79109200	0.05986700
H	-2.13805800	-4.65170000	-0.41344400
S	3.30951700	-1.80648400	0.48805000
I	-1.02390900	-1.58740400	-0.51190300
I	1.36664700	2.12224400	-0.29441300

Product complex of the Diels-Alder reaction involving catalyst **2**

E (hartree) = -2071.935451

G (hartree) = -2071.580214

C	-4.10933600	-0.66692500	0.79858700
C	-5.19056900	0.33032200	0.32607500
C	-6.22656700	-0.41722800	-0.54266400
C	-5.88294600	-1.92259300	-0.57210200
C	-5.73334300	-2.40803700	0.84235900
C	-4.79828700	-1.77520900	1.54585900
H	-7.22967300	-0.27768000	-0.14505300
H	-5.66188700	0.76889400	1.20433800
H	-4.72610400	1.14580600	-0.23653500
H	-3.35616200	-0.16667000	1.41049100
H	-6.64071200	-2.47684900	-1.12103900
H	-6.34540400	-3.20635900	1.24181300
H	-4.56758000	-1.98857000	2.58148200
H	-6.22981400	-0.03380600	-1.56421900
C	-3.45952200	-1.23056900	-0.52419500
H	-3.18425700	-0.36662200	-1.13442800
C	-4.51291700	-2.08376000	-1.26704900
H	-4.58109300	-1.75705400	-2.30514200
H	-4.23854600	-3.13770400	-1.28139400

C	-2.17671900	-1.90769400	-0.15774400
C	-2.15845500	-3.36054100	0.18171000
H	-3.07594800	-3.64487700	0.69732500
H	-2.12798100	-3.93318200	-0.75063600
H	-1.29017400	-3.60992900	0.78732000
O	-1.13480400	-1.24424000	-0.09940000
C	1.09528300	3.51112000	0.02118700
C	-0.30054700	3.48894100	-0.00806500
C	-1.09100800	4.61779500	-0.00444900
C	-0.43568500	5.84316900	0.03126100
C	0.95456600	5.90796400	0.06084900
C	1.72207900	4.75601000	0.05613600
C	1.73705800	2.21337000	0.01439200
H	-2.17125900	4.57099500	-0.02744000
H	-1.02029000	6.75317500	0.03598800
H	1.44277800	6.87252400	0.08825400
H	2.80318700	4.81901400	0.07984300
C	0.99001100	1.06380800	-0.01766200
C	3.57204200	-2.18862300	-0.00209000
C	4.10148700	-0.89676500	0.02223800
C	5.48942400	-0.76713500	0.04758800
C	6.28221000	-1.90188700	0.04777800
C	5.71476800	-3.17279700	0.02300900
C	4.33451300	-3.33647400	-0.00226800
C	1.78436700	-0.10132600	-0.01418700
C	3.12741300	0.17427800	0.01623700
H	5.94245500	0.21660400	0.06659000
H	7.35854900	-1.79890900	0.06730600
H	6.34895300	-4.04901800	0.02322800
H	3.89714900	-4.32539400	-0.02120100
S	3.41773600	1.86582900	0.04432200
I	1.47467300	-2.13936400	-0.02984100
I	-1.02047400	1.51881700	-0.06728300

Transition state of the Diels-Alder reaction involving catalyst **6**

E (hartree) = -2464.622029

G (hartree) = -2464.107300

C	5.45016200	-1.02570800	-1.51962900
C	6.01365000	0.35280800	-1.68898000
C	7.15655400	0.68300600	-0.70392100

C	7.48284000	-0.45227200	0.24325100
C	7.26297700	-1.76207200	-0.15635600
C	6.19192200	-2.05289300	-1.01933900
H	8.06697400	0.89935900	-1.26393800
H	6.39068300	0.39734800	-2.71533100
H	5.21493700	1.09269700	-1.64138900
H	4.51624200	-1.23810800	-2.02922000
H	8.31323800	-0.26911900	0.91567100
H	7.77991300	-2.56998300	0.34862200
H	5.86714600	-3.07774500	-1.14484900
H	6.93154500	1.58542200	-0.13367200
C	4.77330000	-0.14721300	0.91123400
H	4.49760100	0.83486900	0.54514000
C	5.99365500	-0.30021500	1.57262700
H	6.45156900	0.59942500	1.96732500
H	6.15877700	-1.16958400	2.19365400
C	3.76646500	-1.14262300	0.80553400
C	4.00544300	-2.54376200	1.29898900
H	5.05779400	-2.80693600	1.36288200
H	3.56640000	-2.63790000	2.29566400
H	3.50062600	-3.24797200	0.63808500
O	2.62043400	-0.87353600	0.36102800
H	-1.74663500	2.48806100	3.01780000
C	-2.00142800	1.58751200	2.47381900
C	-2.54051700	-0.70413500	1.03848300
C	-1.91451400	1.59590900	1.08836000
C	-2.38646600	0.43798500	3.14077800
C	-2.62840100	-0.72089800	2.41992100
C	-2.23931800	0.46631100	0.33761700
H	-2.45850700	0.43299000	4.21950200
H	-2.87289400	-1.64893000	2.92072100
N	-2.71728700	-1.94519100	0.34471000
N	-1.42177900	2.78902100	0.47365600
C	-0.17315900	2.95491000	-0.00733400
N	-0.06907800	4.17896300	-0.51807600
C	-2.15177300	3.95778800	0.26485500
C	-1.28618100	4.84216800	-0.36824200
C	-1.71566400	-2.78004200	-0.01093300
N	-2.24146200	-3.88151100	-0.53820900
C	-3.94000300	-2.55095700	0.05096900

C	-3.63226400	-3.78058500	-0.51837000
C	1.10148800	4.78625400	-1.13785400
H	1.39010200	5.66788300	-0.56822600
H	1.91579400	4.06901600	-1.14324300
H	0.85456300	5.06946000	-2.15952300
C	-1.53148600	-5.03875400	-1.06880000
H	-1.77387300	-5.15146200	-2.12405300
H	-0.46324600	-4.88879800	-0.95092100
H	-1.84102100	-5.92647600	-0.52020100
C	-1.68418600	6.12188700	-0.73383000
C	-3.46497400	4.29496800	0.56184300
C	-5.24877200	-2.12558900	0.22741200
C	-4.62072300	-4.65804400	-0.94647800
H	-1.01393000	6.81389100	-1.22485100
H	-4.13739500	3.60202400	1.04899700
H	-4.38603800	-5.61462800	-1.39261500
H	-5.48256600	-1.16453800	0.66462200
C	-2.98922800	6.46415800	-0.43593100
C	-3.86319800	5.56750200	0.19855600
C	-6.23386500	-2.99813900	-0.19392700
C	-5.92578300	-4.24038800	-0.77051900
H	-3.34923300	7.44901600	-0.69944400
H	-4.87603200	5.88313900	0.40753100
H	-6.73316200	-4.88660000	-1.08599200
H	-7.27169500	-2.71707400	-0.07969300
C	-2.33497700	0.45630400	-1.18094300
I	1.26650900	1.45804300	0.09051000
I	0.28544900	-2.28958900	0.23176800
F	-3.51926100	-0.03559600	-1.54753800
F	-1.39563500	-0.30845500	-1.74033800
F	-2.22812200	1.66976500	-1.71320900

Starting material complex of the Diels-Alder reaction involving catalyst **6**

E (hartree) = -2464.646203

G (hartree) = -2464.134755

C	3.17583700	-0.32594000	-1.70167700
C	3.75020700	0.95308500	-2.25026500
C	4.92558500	0.68410000	-3.19045100
C	5.84601300	-0.37482900	-2.64766500
C	5.37330100	-1.35987900	-1.88183100

C	3.95201600	-1.39763900	-1.52677400
H	4.55258200	0.33595300	-4.16163300
H	2.98017200	1.52734600	-2.76786300
H	4.09003000	1.57291700	-1.40939100
H	2.12640700	-0.35733600	-1.42825300
H	6.88350800	-0.37048200	-2.95929700
H	6.02050900	-2.16671200	-1.55849500
H	3.54037900	-2.31543300	-1.11937900
H	5.47091500	1.60705700	-3.38615300
C	4.87176600	0.80642000	1.17109100
H	4.34299400	1.75329600	1.16545500
C	6.15404400	0.73566000	0.82902900
H	6.70643600	1.61999600	0.53884900
H	6.69907800	-0.19942500	0.81991100
C	4.06240700	-0.36192200	1.54066400
C	4.72591600	-1.68790700	1.75429400
H	5.29245200	-1.97486400	0.86784500
H	5.42873800	-1.61614400	2.58648500
H	3.98117400	-2.44744900	1.97860900
O	2.84929800	-0.23502700	1.69254000
H	-2.72029600	2.28352600	2.81146200
C	-2.64490100	1.34152200	2.28334200
C	-2.34201400	-1.03535400	0.91931500
C	-2.17702400	1.34231300	0.97620900
C	-2.98632500	0.15092700	2.90028200
C	-2.80759300	-1.04530400	2.22290800
C	-2.06714400	0.15840900	0.24734000
H	-3.35360100	0.15082300	3.91717000
H	-3.01447000	-1.99388800	2.70174300
N	-2.10222800	-2.29846600	0.28625000
N	-1.76156000	2.59772300	0.43289800
C	-0.48279900	2.99396400	0.28128000
N	-0.46547800	4.20282800	-0.27209500
C	-2.60709100	3.59868200	-0.04330600
C	-1.77771200	4.62048800	-0.49136600
C	-0.91757100	-2.94656200	0.27392600
N	-1.07387100	-4.12715300	-0.31567900
C	-3.06885500	-3.11410200	-0.30520100
C	-2.40840400	-4.27338200	-0.69371900
C	0.69886000	5.01503900	-0.60465000

H	0.67510400	5.93135200	-0.01740300
H	1.60213200	4.45715200	-0.37964600
H	0.66959500	5.25348000	-1.66625500
C	-0.05884500	-5.14558000	-0.55966800
H	0.03562200	-5.30184800	-1.63280100
H	0.89004500	-4.81322300	-0.15150600
H	-0.36330700	-6.07102100	-0.07431700
C	-2.28726500	5.79179500	-1.03788000
C	-3.99039000	3.68130700	-0.12264700
C	-4.42547000	-2.92653500	-0.52853600
C	-3.06971000	-5.31572400	-1.33176800
H	-1.64661600	6.59094800	-1.38448400
H	-4.63237100	2.88225600	0.22226900
H	-2.55961900	-6.21863600	-1.63788900
H	-4.93375900	-2.01995600	-0.22991800
C	-3.66370200	5.88078600	-1.11471100
C	-4.49910800	4.84505500	-0.66697300
C	-5.08605900	-3.96300700	-1.15935500
C	-4.42082800	-5.13496400	-1.55409300
H	-4.11176400	6.77211300	-1.53162600
H	-5.57057300	4.96365400	-0.75107200
H	-4.98325700	-5.91638700	-2.04609900
H	-6.14497300	-3.87048700	-1.35758500
C	-1.72113400	0.09986200	-1.23284300
I	1.12376300	1.82396600	0.85834300
I	0.81298900	-2.13167800	1.05158100
F	-2.65408900	-0.59630900	-1.88165700
F	-0.55023400	-0.50684400	-1.44866300
F	-1.65452600	1.29995000	-1.79973200

Product complex of the Diels-Alder reaction involving catalyst **6**

E (hartree) = -2464.691910

G (hartree) = -2464.171026

C	4.94682500	-0.79658700	-0.93980500
C	5.89787100	0.41930600	-0.90644900
C	7.22224500	0.01425000	-0.22210800
C	7.17036900	-1.48446800	0.14618500
C	6.81530900	-2.26570600	-1.08817700
C	5.65558400	-1.92228900	-1.64224000
H	8.06781500	0.20014000	-0.88174200

H	6.07513400	0.74984000	-1.92923300
H	5.41618300	1.24330600	-0.37323000
H	4.00093600	-0.53629200	-1.41665900
H	8.11575400	-1.80462600	0.57866400
H	7.46290900	-3.03817900	-1.48258000
H	5.25449800	-2.37531100	-2.53973500
H	7.38740100	0.59702300	0.68562200
C	4.69964500	-1.18972500	0.56072300
H	4.35826700	-0.28294000	1.06773900
C	6.03069000	-1.67956500	1.17063200
H	6.25114500	-1.11531200	2.07761600
H	5.97971500	-2.72967900	1.45432500
C	3.53215000	-2.13858300	0.60312100
C	3.74706400	-3.61648000	0.64563600
H	4.56001000	-3.90288100	-0.02254300
H	4.05033900	-3.89797400	1.65768900
H	2.82966200	-4.14097600	0.39038800
O	2.39424400	-1.68005800	0.56230400
H	-1.26654200	2.71942200	3.02083100
C	-1.69820700	1.89349200	2.46999200
C	-2.69546400	-0.23074100	1.02246200
C	-1.54949100	1.85868200	1.09008900
C	-2.36938300	0.87558400	3.12423300
C	-2.84602100	-0.20493500	2.39844600
C	-2.09292600	0.82287700	0.33042900
H	-2.48824800	0.90457100	4.19847400
H	-3.32693700	-1.03992700	2.89183000
N	-3.14447400	-1.39933600	0.32512900
N	-0.76813900	2.89636500	0.49241500
C	0.50279400	2.76288400	0.06608400
N	0.91186200	3.91779000	-0.45011600
C	-1.19751100	4.19844400	0.24011100
C	-0.12435500	4.84660800	-0.36084400
C	-2.37456600	-2.46522900	0.01678900
N	-3.13497000	-3.40554900	-0.53455800
C	-4.46607000	-1.68047500	-0.02681100
C	-4.45401400	-2.95500600	-0.58012800
C	2.21807900	4.22808600	-1.01942900
H	2.67632200	5.02922100	-0.44227800
H	2.84591400	3.34345200	-0.98300800

H	2.08702000	4.54301000	-2.05308200
C	-2.72177200	-4.71390500	-1.02954400
H	-2.94901900	-4.77800500	-2.09213500
H	-1.65516600	-4.83803200	-0.87392200
H	-3.26495900	-5.48487600	-0.48606400
C	-0.19862100	6.17691000	-0.75474000
C	-2.40795600	4.83578400	0.47538500
C	-5.63117700	-0.93554700	0.08733600
C	-5.61165800	-3.56022200	-1.05380800
H	0.63379100	6.68586000	-1.22084100
H	-3.24238200	4.32699500	0.93846500
H	-5.60669700	-4.55094100	-1.48694600
H	-5.63425200	0.05871200	0.51259800
C	-1.39985000	6.81683400	-0.51857900
C	-2.48394700	6.15870500	0.08376400
C	-6.78424900	-1.53644300	-0.37950600
C	-6.77469100	-2.82393200	-0.93993400
H	-1.51025000	7.85327000	-0.80607700
H	-3.40348000	6.70439300	0.24453400
H	-7.70435000	-3.24901700	-1.29214300
H	-7.72149400	-1.00118300	-0.31434600
C	-2.11189100	0.80278700	-1.19085600
I	1.55658300	0.98970900	0.23799600
I	-0.34026800	-2.52065600	0.35983400
F	-3.36103900	0.61740900	-1.61775500
F	-1.36956800	-0.19039900	-1.68591700
F	-1.67122500	1.93458300	-1.73037500

4. XRD Data

Table S1: Crystallograpic data of the triflate salt of the bidentate catalyst **1**

Compound	**1**
CCDC number	2033153
Empirical formula	C18 H8 F6 I2 O6 S3
Formula weight [g/mol]	784.22
Crystal system	Monoclinic
Space group	$P2_1/n$ (14)
Lattice parameters [Å]	
a	12.6977(7)
b	10.2824(6)
c	17.2846(8)
α	90
β	91.824(4)
γ	90
Density [g/cm^3]	2.309
Crystal size [mm^3]	0.337 x 0.095 x 0.017
Volume [Å^3]	2255.6(2)
Z	4
Temperature [K]	170(2)
Diffraction Device	XtaLAB Mini (ROW)
Radiation Type	0.71073 Å (Mo K/ fine-focus sealed X-ray tube)
F(000)	1488
Absorption coefficient [mm^{-1}]	3.152
Absorption correction	Gaussian
Measurement range	2.3 - 26.5
Index range	-15 < h < 15
	-10 < k < 12
	-21 < l < 19
Measured reflexes	9514
Independent	4646
Observed	3887
R(int)	0.0317
Completeness (%) / theta (°)	99.4 / 25.242
Transmission (min / max)	0.540 / 1.000
R1 (observed/all)	0.0381 / 0.0486
wR2 (observed/all)	0.0942 / 0.1022
GooF = S	1.048
Rest electron density max./min. [e-/Å^3]	-0.971 / 1.856

4.1 Crystal Growth

In a 1.5 ml screw cap vile, 5 mg of **1** was dissolved in acetonitrile and the solvent then slowly evaporated until crystals were obtained.

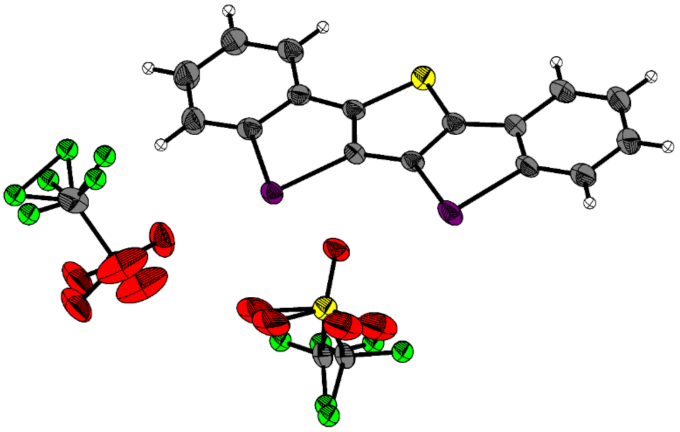

Figure S42: Asymmetric unit of **1** with ellipsoids at their 50% probability level. The triflate anions are disordered.

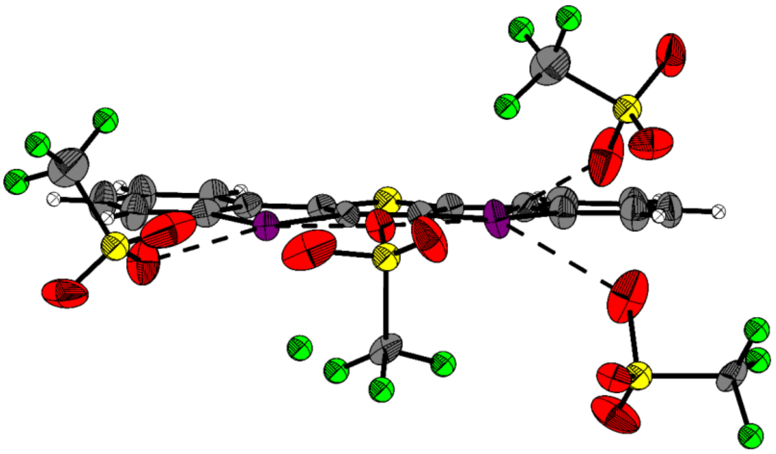

Figure S43: Front view of crystal structure **1** to show the four triflates coordinated to the bis(iodolium) cation.

Table S2: Atomic coordinates and equivalent isotropic displacement parameters [Å2] for catalyst **1**.

	x	y	z	U(eq)	S.O.F
I11	0.21089(2)	0.51173(3)	0.44011(2)	0.02612(11)	1
I21	0.41601(2)	0.80849(4)	0.33421(2)	0.03536(12)	1
S11	0.49177(9)	0.66836(12)	0.57454(7)	0.0283(3)	1
C11	0.3276(4)	0.4809(5)	0.5872(3)	0.0276(10)	1
C21	0.3548(4)	0.4333(5)	0.6604(3)	0.0343(12)	1
C31	0.2956(4)	0.3352(6)	0.6930(3)	0.0397(13)	1
C41	0.2088(5)	0.2844(6)	0.6534(4)	0.0438(14)	1
C51	0.1778(4)	0.3306(5)	0.5811(3)	0.0376(12)	1
C61	0.2386(4)	0.4273(5)	0.5493(3)	0.0293(10)	1
C71	0.3815(4)	0.5806(5)	0.5451(3)	0.0253(10)	1
C81	0.3471(3)	0.6138(5)	0.4714(3)	0.0248(10)	1
C91	0.4105(4)	0.7127(5)	0.4392(3)	0.0250(10)	1
C101	0.4919(4)	0.7513(5)	0.4875(3)	0.0269(10)	1
C111	0.5466(4)	0.8997(5)	0.3898(3)	0.0328(11)	1
C121	0.5645(4)	0.8512(5)	0.4635(3)	0.0306(11)	1
C131	0.6067(4)	0.9962(5)	0.3565(4)	0.0397(13)	1
C141	0.6877(4)	1.0465(6)	0.4022(4)	0.0403(13)	1
C151	0.7094(4)	1.0005(5)	0.4763(4)	0.0367(13)	1
C161	0.6491(4)	0.9034(5)	0.5079(3)	0.0353(12)	1
S1A2	0.17095(11)	0.69122(14)	0.25059(8)	0.0362(3)	0.656(7)
F1A2	0.0615(6)	0.7821(11)	0.3668(4)	0.077(3)	0.656(7)
F2A2	0.007(4)	0.853(5)	0.256(3)	0.062(4)	0.656(7)
F3A2	0.1590(7)	0.9148(8)	0.3153(8)	0.105(4)	0.656(7)
O1A2	0.1071(8)	0.6016(9)	0.2137(6)	0.068(3)	0.656(7)
O2A2	0.2222(7)	0.7855(11)	0.1959(6)	0.066(3)	0.656(7)
O3A2	0.2481(3)	0.6519(4)	0.3098(2)	0.0336(8)	0.656(7)
C1A2	0.0971(10)	0.8143(11)	0.3014(8)	0.033(2)	0.656(7)
S1B2	0.17095(11)	0.69122(14)	0.25059(8)	0.0362(3)	0.344(7)
F1B2	0.0312(14)	0.700(2)	0.3488(9)	0.077(3)	0.344(7)
F2B2	0.017(8)	0.836(10)	0.263(6)	0.062(4)	0.344(7)
F3B2	0.1267(16)	0.8649(19)	0.349(2)	0.105(4)	0.344(7)
O1B2	0.0906(17)	0.576(2)	0.2558(11)	0.068(3)	0.344(7)
O2B2	0.1924(15)	0.719(2)	0.1817(12)	0.066(3)	0.344(7)
O3B2	0.2481(3)	0.6519(4)	0.3098(2)	0.0336(8)	0.344(7)
C1B2	0.075(2)	0.774(2)	0.2995(19)	0.033(2)	0.344(7)
S1A3	−0.03942(10)	0.31989(14)	0.38446(8)	0.0358(3)	0.869(9)
F1A3	0.1094(4)	0.1485(6)	0.3929(5)	0.095(3)	0.869(9)
F2A3	−0.0400(5)	0.0777(6)	0.3436(5)	0.092(2)	0.869(9)
F3A3	−0.0182(7)	0.1125(6)	0.4639(5)	0.111(3)	0.869(9)
O1A3	−0.1509(5)	0.3112(6)	0.3935(5)	0.068(2)	0.869(9)
O2A3	−0.0137(7)	0.3620(7)	0.3084(3)	0.081(2)	0.869(9)
O3A3	0.0199(3)	0.3881(4)	0.4432(2)	0.0506(11)	0.869(9)
C1A3	0.0073(5)	0.1562(7)	0.3933(5)	0.0579(19)	0.869(9)

S1B3	−0.03942(10)	0.31989(14)	0.38446(8)	0.0358(3)	0.131(9)
F1B3	0.055(3)	0.097(4)	0.449(4)	0.095(3)	0.131(9)
F2B3	0.090(3)	0.140(3)	0.334(3)	0.092(2)	0.131(9)
F3B3	−0.044(5)	0.044(5)	0.386(4)	0.111(3)	0.131(9)
O1B3	−0.124(4)	0.290(5)	0.428(4)	0.068(2)	0.131(9)
O2B3	−0.005(6)	0.300(5)	0.309(3)	0.081(2)	0.131(9)
O3B3	0.0199(3)	0.3881(4)	0.4432(2)	0.0506(11)	0.131(9)
C1B3	0.0073(5)	0.1562(7)	0.3933(5)	0.0579(19)	0.131(9)
H21	0.414079	0.468	0.68818	0.041	1
H31	0.315038	0.303	0.742881	0.048	1
H41	0.16967	0.217	0.676314	0.053	1
H51	0.117231	0.297	0.554252	0.045	1
H131	0.592712	1.026	0.305102	0.048	1
H141	0.729697	1.115	0.382406	0.048	1
H151	0.766738	1.036	0.505892	0.044	1
H161	0.664484	0.872	0.558799	0.042	1
F3B2	0.1267(16)	0.8649(19)	0.349(2)	0.105(4)	0.344(7)
O1B2	0.0906(17)	0.576(2)	0.2558(11)	0.068(3)	0.344(7)
O2B2	0.1924(15)	0.719(2)	0.1817(12)	0.066(3)	0.344(7)
O3B2	0.2481(3)	0.6519(4)	0.3098(2)	0.0336(8)	0.344(7)
O3B3	0.0199(3)	0.3881(4)	0.4432(2)	0.0506(11)	0.131(9)
C1B3	0.0073(5)	0.1562(7)	0.3933(5)	0.0579(19)	0.131(9)
H21	0.414079	0.468	0.68818	0.041	1
H31	0.315038	0.303	0.742881	0.048	1
H41	0.16967	0.217	0.676314	0.053	1
H51	0.117231	0.297	0.554252	0.045	1
H131	0.592712	1.026	0.305102	0.048	1
H141	0.729697	1.115	0.382406	0.048	1
H151	0.766738	1.036	0.505892	0.044	1

Table S3: Anisotropic displacement parameters [Å2] for catalyst **1**.

	U11	U22	U33	U23	U13	U12
I11	0.02602(18)	0.03109(19)	0.02083(17)	0.00004(12)	– 0.00567(12)	– 0.00421(11)
I21	0.02584(18)	0.0525(2)	0.02715(19)	0.01317(15)	– 0.00866(13)	– 0.01105(13)
S11	0.0269(6)	0.0331(7)	0.0241(6)	0.0029(5)	−0.0088(5)	−0.0040(5)
C11	0.028(2)	0.026(3)	0.028(3)	−0.002(2)	−0.005(2)	0.0033(19)
C21	0.033(3)	0.041(3)	0.028(3)	0.003(2)	−0.007(2)	−0.003(2)
C31	0.041(3)	0.046(3)	0.032(3)	0.013(2)	−0.003(2)	−0.007(2)
C41	0.049(3)	0.045(3)	0.037(3)	0.014(3)	−0.005(3)	−0.011(3)
C51	0.039(3)	0.039(3)	0.035(3)	0.006(2)	−0.008(2)	−0.011(2)
C61	0.030(2)	0.032(3)	0.026(3)	0.001(2)	−0.004(2)	0.002(2)
C71	0.024(2)	0.027(3)	0.025(2)	−0.0028(19)	−0.0074(18)	0.0012(18)
C81	0.021(2)	0.028(3)	0.024(2)	−0.0013(19)	−0.0019(18)	−0.0016(18)
C91	0.024(2)	0.031(3)	0.019(2)	0.0030(19)	−0.0043(18)	0.0003(18)

C101	0.026(2)	0.032(3)	0.023(2)	0.002(2)	−0.0043(19)	−0.0005(19)
C111	0.022(2)	0.039(3)	0.036(3)	0.007(2)	−0.009(2)	−0.005(2)
C121	0.023(2)	0.030(3)	0.039(3)	0.002(2)	−0.005(2)	−0.0022(19)
C131	0.029(3)	0.044(3)	0.046(3)	0.013(3)	−0.007(2)	−0.003(2)
C141	0.029(3)	0.040(3)	0.052(4)	0.012(3)	0.000(2)	−0.007(2)
C151	0.026(3)	0.042(3)	0.042(3)	0.001(2)	−0.005(2)	−0.007(2)
C161	0.028(3)	0.042(3)	0.035(3)	0.000(2)	−0.010(2)	−0.001(2)
S1A2	0.0322(7)	0.0504(9)	0.0252(6)	−0.0074(6)	−0.0106(5)	0.0083(5)
F1A2	0.066(5)	0.135(9)	0.031(4)	−0.002(4)	0.009(3)	0.051(6)
F2A2	0.059(9)	0.081(13)	0.046(9)	0.008(7)	−0.013(6)	0.050(6)
F3A2	0.061(6)	0.048(6)	0.207(13)	−0.048(6)	0.001(6)	−0.004(3)
O1A2	0.069(5)	0.060(5)	0.071(7)	−0.027(6)	−0.052(6)	0.012(4)
O2A2	0.042(5)	0.101(9)	0.056(5)	0.047(6)	0.007(4)	0.006(4)
O3A2	0.0275(18)	0.046(2)	0.0270(19)	0.0077(16)	−0.0066(14)	−0.0024(15)
C1A2	0.035(6)	0.024(8)	0.040(4)	−0.015(6)	−0.018(4)	−0.007(4)
S1B2	0.0322(7)	0.0504(9)	0.0252(6)	−0.0074(6)	−0.0106(5)	0.0083(5)
F1B2	0.066(5)	0.135(9)	0.031(4)	−0.002(4)	0.009(3)	0.051(6)
F2B2	0.059(9)	0.081(13)	0.046(9)	0.008(7)	−0.013(6)	0.050(6)
F3B2	0.061(6)	0.048(6)	0.207(13)	−0.048(6)	0.001(6)	−0.004(3)
O1B2	0.069(5)	0.060(5)	0.071(7)	−0.027(6)	−0.052(6)	0.012(4)
O2B2	0.042(5)	0.101(9)	0.056(5)	0.047(6)	0.007(4)	0.006(4)
O3B2	0.0275(18)	0.046(2)	0.0270(19)	0.0077(16)	−0.0066(14)	−0.0024(15)
C1B2	0.035(6)	0.024(8)	0.040(4)	−0.015(6)	−0.018(4)	−0.007(4)
S1A3	0.0300(7)	0.0466(8)	0.0306(7)	−0.0135(6)	−0.0039(5)	0.0012(5)
F1A3	0.043(3)	0.071(4)	0.168(8)	−0.017(4)	−0.022(4)	0.016(2)
F2A3	0.097(4)	0.051(4)	0.125(6)	−0.048(3)	−0.051(4)	0.029(3)
F3A3	0.137(7)	0.068(4)	0.126(6)	0.047(4)	−0.008(5)	−0.015(4)
O1A3	0.025(3)	0.058(4)	0.118(7)	−0.043(4)	−0.005(3)	0.002(2)
O2A3	0.147(6)	0.066(5)	0.030(3)	0.003(3)	0.009(3)	0.047(5)
O3A3	0.054(3)	0.055(3)	0.041(2)	−0.004(2)	−0.013(2)	−0.023(2)
C1A3	0.043(4)	0.051(4)	0.078(5)	0.001(4)	−0.022(4)	0.002(3)
S1B3	0.0300(7)	0.0466(8)	0.0306(7)	−0.0135(6)	−0.0039(5)	0.0012(5)
F1B3	0.043(3)	0.071(4)	0.168(8)	−0.017(4)	−0.022(4)	0.016(2)
F2B3	0.097(4)	0.051(4)	0.125(6)	−0.048(3)	−0.051(4)	0.029(3)
F3B3	0.137(7)	0.068(4)	0.126(6)	0.047(4)	−0.008(5)	−0.015(4)
O1B3	0.025(3)	0.058(4)	0.118(7)	−0.043(4)	−0.005(3)	0.002(2)
O2B3	0.147(6)	0.066(5)	0.030(3)	0.003(3)	0.009(3)	0.047(5)
O3B3	0.054(3)	0.055(3)	0.041(2)	−0.004(2)	−0.013(2)	−0.023(2)
C1B3	0.043(4)	0.051(4)	0.078(5)	0.001(4)	−0.022(4)	0.002(3)

5. Literature

[S1] M. R. Willcott, *J. Am. Chem. Soc.* **2009**, *131*, 13180.

[S2] a) B. Putz, K. Brandenburg, *Diamond*, Crystal Impact, Bonn, **2014**; b) G. M. Sheldrick, *Acta Crystallogr A Found Crystallogr* **2008**, *64*, 112; c) C. B. Hübschle, G. M. Sheldrick, B. Dittrich, *J. Appl. Crystallogr.* **2011**, *44*, 1281; d) L. J. Farrugia, *J. Appl. Crystallogr.* **1999**, *32*, 837.

[S3] F. Heinen, E. Engelage, A. Dreger, R. Weiss, S. M. Huber, *Angew. Chem. Int. Ed.* **2018**, *57*, 3830.

[S4] J.-P. Gliese, S. H. Jungbauer, S. M. Huber, *Chem. Commun.* **2017**, *53*, 12052.

[S5] A. Wittkopp, P. R. Schreiner, *Chem. Eur. J.* **2003**, *9*, 407.

[S6] B. Wu, N. Yoshikai, *Angew. Chem. Int. Ed.* **2015**, *54*, 8736.

[S7] J. Wolf, F. Huber, N. Erochok, F. Heinen, V. Guérin, C. Y. Legault, S. F. Kirsch, S. M. Huber, *Angew. Chem. Int. Ed.* **2020**, *59*, 16496.

[S8] P. Wonner, T. Steinke, L. Vogel, S. M. Huber, *Chem. Eur. J.* **2020**, *26*, 1258.

[S9] P. Wonner, A. Dreger, L. Vogel, E. Engelage, S. M. Huber, *Angew. Chem. Int. Ed.* **2019**, *58*, 16923.

[S10] M. J. Frisch, G. W. Trucks, H. B. Schlegel, G. E. Scuseria, M. A. Robb, J. R. Cheeseman, G. Scalmani, V. Barone, G. A. Petersson, H. Nakatsuji et al., *Gaussian 16 Revision B01*, Gaussian, Inc, Wallingford, CT, USA, **2009**.

[S11] a) S. Grimme, J. Antony, S. Ehrlich, H. Krieg, *J. Chem. Phys.* **2010**, *132*, 154104; b) Y. Zhao, D. G. Truhlar, *Theor. Chem. Acc.* **2008**, *120*, 215.

[S12] a) F. Weigend, R. Ahlrichs, *Phys. Chem. Chem. Phys.* **2005**, *7*, 3297; b) D. Rappoport, F. Furche, *J. Chem. Phys.* **2010**, *133*, 134105.

[S13] K. A. Peterson, D. Figgen, E. Goll, H. Stoll, M. Dolg, *J. Chem. Phys.* **2003**, *119*, 11113.

[S14] S. Grimme, *Chem. Eur. J.* **2012**, *18*, 9955.
